# Supplementary material for: Higher Dose Oral Fluconazole for the Treatment of AIDS-related Cryptococcal Meningitis (HIFLAC)—report of A5225, a multicentre, phase I/II, two-stage, dose-finding, safety, tolerability and efficacy randomised, amphotericin B-controlled trial of the AIDS Clinical Trials Group
Source: PLoS One. 2023 Feb 13;18(2):e0281580. doi: 10.1371/journal.pone.0281580 (PMC9925064; doi:10.1371/journal.pone.0281580)
Supplement: S1 File — (DOC) [file pone.0281580.s002.doc]

**A5225/HiFLAC**

**A Phase I/II Dose-Finding Study of High-Dose Fluconazole Treatment in AIDS-Associated Cryptococcal Meningitis**

**A Multicenter Trial of the AIDS Clinical Trials Group (ACTG)**

**Sponsored by:**

**The National Institute of Allergy and Infectious Diseases**

**Industry Support Provided by:**

**Pfizer**

**IND # 105,128**

**The ACTG Co-Infections and Malignancies Subcommittee of the**

**End-organ Disease/Inflammation**

**Transformative Science Group: Charles van der Horst, MD, Chair**

**Protocol Co-Chairs: Umesh Lalloo, MD**

**Robert Larsen, MD**

Protocol Vice Chair: Judith A. Aberg, MD

DAIDS Clinical Representative: Trinh Ly, MD

**Clinical Trials Specialist: Evelyn Hogg, BA**

**DRAFT Version 2.01**

**October 25, 2013**

TABLE OF CONTENTS

Page

SITES PARTICIPATING IN THE STUDY [5](#__RefHeading___Toc324518529)

PROTOCOL TEAM ROSTER [6](#__RefHeading___Toc324518530)

STUDY MANAGEMENT [10](#__RefHeading___Toc324518531)

GLOSSARY OF STUDY-SPECIFIC TERMS [13](#__RefHeading___Toc324518532)

SCHEMA [14](#__RefHeading___Toc324518533)

1.0 HYPOTHESES AND STUDY OBJECTIVES [24](#__RefHeading___Toc324518534)

1.1 Hypotheses [24](#__RefHeading___Toc324518535)

1.2 Primary Objectives [24](#__RefHeading___Toc324518536)

1.3 Secondary Objectives [24](#__RefHeading___Toc324518537)

1.4 Exploratory Objectives [25](#__RefHeading___Toc324518538)

2.0 INTRODUCTION [25](#__RefHeading___Toc324518539)

2.1 Background [25](#__RefHeading___Toc324518540)

2.2 Rationale [38](#__RefHeading___Toc324518541)

3.0 STUDY DESIGN [44](#__RefHeading___Toc324518542)

4.0 SELECTION AND ENROLLMENT OF PARTICIPANTS [46](#__RefHeading___Toc324518543)

4.1 Inclusion Criteria – Step 1 [46](#__RefHeading___Toc324518544)

4.2 Exclusion Criteria - Step 1 [48](#__RefHeading___Toc324518545)

4.3 Inclusion Criteria – Step 2 [49](#__RefHeading___Toc324518546)

4.4 Exclusion Criteria – Step 2 [50](#__RefHeading___Toc324518547)

4.5 Inclusion Criteria - Step 3 [50](#__RefHeading___Toc324518548)

4.6 Exclusion Criteria – Step 3 [50](#__RefHeading___Toc324518549)

4.7 Inclusion Criterion – Step 4 [50](#__RefHeading___Toc324518550)

4.8 Exclusion Criterion – Step 4 [50](#__RefHeading___Toc324518551)

4.9 Study Registration and Enrollment Procedures [50](#__RefHeading___Toc324518552)

4.10 Coenrollment Guidelines [51](#__RefHeading___Toc324518553)

5.0 STUDY TREATMENT [51](#__RefHeading___Toc324518554)

5.1 Regimens, Administration, and Duration [51](#__RefHeading___Toc324518555)

5.2 Product Formulation and Preparation [56](#__RefHeading___Toc324518556)

5.3 Pharmacy: Product Supply, Distribution, and Accountability [56](#__RefHeading___Toc324518557)

5.4 Concomitant Medications [56](#__RefHeading___Toc324518558)

5.5 Adherence Assessment [57](#__RefHeading___Toc324518559)

6.0 CLINICAL AND LABORATORY EVALUATIONS [58](#__RefHeading___Toc324518560)

6.1 Schedule of Events [58](#__RefHeading___Toc324518561)

6.2 Timing of Evaluations [63](#__RefHeading___Toc324518562)

6.3 Special Instructions and Definitions of Evaluations [66](#__RefHeading___Toc324518563)

7.0 CLINICAL MANAGEMENT ISSUES [74](#__RefHeading___Toc324518564)

7.1 General Toxicities [75](#__RefHeading___Toc324518565)

7.2 Fluconazole-Induced Prolongation in QT Interval [75](#__RefHeading___Toc324518566)

7.3 Fluconazole-Induced Hepatotoxicity [76](#__RefHeading___Toc324518567)

7.4 Fluconazole Dose Modifications [77](#__RefHeading___Toc324518568)

7.5 Fluconazole-Induced Rash [78](#__RefHeading___Toc324518569)

7.6 Adrenal Insufficiency (AI) [78](#__RefHeading___Toc324518570)

7.7 Pregnancy [79](#__RefHeading___Toc324518571)

8.0 CRITERIA FOR PREMATURE TREATMENT OR STUDY DISCONTINUATION [79](#__RefHeading___Toc324518572)

8.1 Treatment Discontinuation [79](#__RefHeading___Toc324518573)

8.2 Study Discontinuation [80](#__RefHeading___Toc324518574)

9.0 STATISTICAL CONSIDERATIONS [80](#__RefHeading___Toc324518575)

9.1 General Design Issues [80](#__RefHeading___Toc324518576)

9.2 Outcome Measures [81](#__RefHeading___Toc324518577)

9.3 Sample Size and Criteria for Stages 1 and 2 [82](#__RefHeading___Toc324518578)

9.4 Monitoring [87](#__RefHeading___Toc324518579)

9.5 Analysis [88](#__RefHeading___Toc324518580)

10.0 PHARMACOLOGY PLAN [88](#__RefHeading___Toc324518581)

10.1 Pharmacology Objectives [89](#__RefHeading___Toc324518582)

10.2 Pharmacology Study Design [90](#__RefHeading___Toc324518583)

10.3 Primary and Secondary Data Analysis [92](#__RefHeading___Toc324518584)

10.4 Adequacy of Sample Size [92](#__RefHeading___Toc324518585)

10.5 Anticipated Outcomes [92](#__RefHeading___Toc324518586)

11.0 DATA COLLECTION AND MONITORING AND ADVERSE EVENT REPORTING [93](#__RefHeading___Toc324518587)

11.1 Records to Be Kept [93](#__RefHeading___Toc324518588)

11.2 Role of Data Management [93](#__RefHeading___Toc324518589)

11.3 Clinical Site Monitoring and Record Availability [93](#__RefHeading___Toc324518590)

11.4 Expedited Adverse Event (EAE) Reporting [93](#__RefHeading___Toc324518591)

12.0 HUMAN PARTICIPANTS [94](#__RefHeading___Toc324518592)

12.1 Institutional Review Board (IRB) Review and Informed Consent [94](#__RefHeading___Toc324518593)

12.2 Participant Confidentiality [95](#__RefHeading___Toc324518594)

12.3 Study Discontinuation [95](#__RefHeading___Toc324518595)

13.0 PUBLICATION OF RESEARCH FINDINGS [95](#__RefHeading___Toc324518596)

14.0 BIOHAZARD CONTAINMENT [95](#__RefHeading___Toc324518597)

15.0 REFERENCES [96](#__RefHeading___Toc324518598)

APPENDIX I: A5225 PARTICIPANT INFORMATION

APPENDIX II: SAMPLE CONCISE INFORMED CONSENT

APPENDIX III: SAMPLE INFORMED CONSENT FOR WOMEN WHO BECOME PREGNANT WHILE ON STUDY

# SITES PARTICIPATING IN THE STUDY

A5225/HiFLAC is a multicenter study open to all ACTG US and non-US clinical research sites (CRSs) that receive approval for their site implementation plan (SIP) (see the Current Version tab on the A5225 protocol specific web page [PSWP] on the ACTG website).

# PROTOCOL TEAM ROSTER

Co-Chairs

Umesh G. Lalloo, MD, FRCP

Nelson R. Mandela School of Medicine

Private Bag 7

Congella

Durban 4013

SOUTH AFRICA

Phone: 27 31 260 4637

FAX: 27 31260 4420

E-mail: [lalloo@nu.ac.za](mailto:lalloo@nu.ac.za)

Robert A. Larsen, MD

USC School of Medicine

2020 Zonal Avenue

Room 632

Los Angeles, CA 90033

Phone: 323-226-2450

FAX: 323-226-2775

E-Mail: [rlarsen@usc.edu](mailto:rlarsen@usc.edu)

Vice Chair

Judith A. Aberg, MD

AIDS Clinical Trials Unit

New York University

Bellevue C and D Building, Room 558

550 First Avenue

New York, NY 10016-6481

Phone: 212-263-6565

FAX: 212-263-8264

E-mail: [judith.aberg@med.nyu.edu](mailto:judith.aberg@med.nyu.edu)

DAIDS Clinical Representative

Trinh Thuy Ly, MD

NIAID/DAIDS/NIH

Complications and Co-Infections Research Branch

6700B Rockledge Drive, Room 4127

Bethesda, MD 20892-7620

Phone: 301-402-2297

FAX: 301-480-4456

E-mail: [tly@niaid.nih.gov](mailto:tly@niaid.nih.gov)

Clinical Trials Specialist

Evelyn Hogg, BA

Social & Scientific Systems, Inc.

ACTG Operations Center

8757 Georgia Avenue, 12^th^ Floor

Silver Spring, MD 20910-3714

Phone: 301-628-3337

FAX: 301-628-3302

E-mail: [**ehogg@s-3.com**](mailto:ehogg@s-3.com)

Statisticians

Janet Andersen, ScD

Statistical & Data Analysis Center

Harvard School of Public Health

FXB Building, Room 621

651 Huntington Avenue

Boston, MA 02115-6017

Phone: 617-432-2814

FAX: 617-432-2843

E-Mail: [**andersen@sdac.harvard.edu**](mailto:andersen@sdac.harvard.edu)

Kimberly Hollabaugh, MS

Statistical & Data Analysis Center

Harvard School of Public Health

FXB Building, Room 549

651 Huntington Avenue

Boston, MA 02115-6017

Phone: 617-432-1352

FAX: 617-432-3163

E-Mail: [hollabau@sdac.harvard.edu](mailto:hollabau@sdac.harvard.edu)

Lauren Komarow, MS

Statistical & Data Analysis Center

Harvard School of Public Health

FXB Building, Room 514

651 Huntington Avenue

Boston, MA 02115-6017

Phone: 617-432-3233

FAX: 617-432-3163

E-mail: [lkomarow@sdac.harvard.edu](mailto:lkomarow@sdac.harvard.edu)

Data Manager

Jennifer Janik, MS

Frontier Science & Technology Research Foundation

IBT Section

4033 Maple Road

Amherst, NY 14226-1056

Phone: 716-834-0900, ext. 7287

FAX: 716-834-8432

E-Mail: [janik.jennifer@fstrf.org](mailto:janik.jennifer@fstrf.org)

DAIDS Pharmacist

Lynette Purdue, PharmD
NIAID/DAIDS/NIH
Pharmaceutical Affairs Branch
6700B Rockledge Drive,  Room 4107
Bethesda, MD 20892-7620
Phone: 301-435-3744
FAX: 301-402-1506
E-mail: [LPurdue@niaid.nih.gov](mailto:LPurdue@niaid.nih.gov)

Neurologist

David B. Clifford, MD

Dept. of Neurology & Neurological Surgery

Washington University School of Medicine

P.O. Box 8111

660 South Euclid Avenue

St. Louis, MO 63110-1010

Phone: 314-747-8423

FAX: 314-747-8427

E-mail: [cliffordd@neuro.wustl.edu](mailto:cliffordd@neuro.wustl.edu)

Pharmacologist

Amanda Corbett, PharmD

University of North Carolina at Chapel Hill

School of Pharmacy

CB# 7360, 3317 Kerr Hall

Chapel Hill, NC 27599

Phone: 919-843-2280

FAX: 919-962-0644

E-mail: [ahcorbet@email.unc.edu](mailto:ahcorbet@email.unc.edu)

Investigators

Halima Dawood, MD

Infectious Diseases

Department of Medicine

Greys Hospital

Townbush Road

Pietermaritzburg, 3201

SOUTH AFRICA

Phone: 27 33 897 3378

FAX: 27 33 897 3755

Email: [dawoodh@ukzn.ac.za](mailto:dawoodh@ukzn.ac.za)

J. Allen McCutchan, MD

University of California San Diego

UCSD Antiviral Research Center

150 West Washington Street

Suite 100

San Diego, CA 92103-6325

Phone: 619-543-8080

FAX: 619-298-0177

E-mail: [amccutchan@ucsd.edu](mailto:amccutchan@ucsd.edu)

Site Investigators

*Site 11101*

*Prudence Ive, FCP (Medicine)*

*University of Witwatersrand*

*CHRU, Helen Joseph Hospital*

*Perth Road*

*Westdene*

*Johannesburg, Gauteng 2042*

*SOUTH AFRICA*

*Phone: 27 11 27-68800*

*FAX: 27 11 48-22130*

*E-mail:* [*Pive@witshealth.co.za*](mailto:Pive@witshealth.co.za)

Site Investigators (Con’t)

*Site 11302*

Aldo J. Lucchetti, MD

Asociacion Civil Impacta - San Miguel Jirón Putumayo 177
San Miguel
Lima 32
PERU

Phone: [511-242-3072](tel:511 242 3072)
FAX: [511-242-2467](tel:051 1 2422467)

E-mail: [alucchetti@impactaperu.org](mailto:alucchetti@impactaperu.org)

*Site 11501*

Patcharaphan Sugandhavesa MD

Research Institute for Health Sciences

110 Intavaroros Road

Chiang Mai 50200

THAILAND

Phone: [665-394-5055, x469](tel:(665) 394-5055x469)
FAX: [665-389-4727](tel:(665) 389-4727)

E-mail: [patcharaphan@rihes-cmu.org](mailto:patcharaphan@rihes-cmu.org)

*Site 12401*

Michael K. Ssemmanda, MB ChB

Joint Clinical Research Centre

Plot 893 Ring Road, Butikiro House

Mengo

PO Box 10005

Kampala

UGANDA

Phone: [256-414-34-2521](tel:(011) 256-414342521)
FAX: [256-414-34-2632](tel:(011) 256-414342632)

E-mail: [mkssemmanda@yahoo.co.uk](mailto:mkssemmanda@yahoo.co.uk)

*Site 12501*

Lazarus Makori Momanyi, MB ChB

Walter Reid Project – Kenya Medical Research Institute

Hospital Road
PO Box 1357
Kericho, Rift Valley 254
KENYA

Phone: [254-522-1064](tel:(011) 254-5221064)

E-mail: [lmakori@wrp-kch.org](mailto:lmakori@wrp-kch.org)

Site Investigators (Con’t)

*Site 12601*

David K. Lagat, MB ChB, MMED

AMPATH at MOI University Teaching Hospital

Eldoret CRS
PO Box 3
Nandi Road
Eldoret, Rift Valley 30100
KENYA

Phone: [072-395-8189](tel:(072) 395-8189)
FAX: [072-220-9795](tel:(072) 220-9795)

E-mail: [1drlagat@gmail.com](mailto:1drlagat@gmail.com)

*Site 12901*

Venance P. Maro, MD, MMED

Kilimanjaro Christian Medical Centre

Sokoine Road
PO Box 3010

Moshi

TANZANIA

Phone: [075-458-1444](tel:(011) 075-4581444)

E-mail: [venmaro@yahoo.co.uk](mailto:venmaro@yahoo.co.uk)

*Site 30313*

James G. Hakim, MD

Department of Medicine

AIDS Research Unit

University of Zimbabwe

Six Tunsgate Road, Northwood

P.O. Box A178

Avondale

Harare

ZIMBABWE

Phone: 26 34 79 1631

FAX: 26 34 25 1017

E-mail: [jhakim@mweb.co.zw](mailto:jhakim@mweb.co.zw)

Site Investigators (Con’t)

*Site 31441*

Vidya Mave, MD, TM, MPH

B.J. Medical College & Sassoon General Hospitals
Clinical Trials Unit
Jai Prakash Narayan Road
Pune, Maharastra 411001
INDIA

Phone: [91-20-260-52419](tel:011-91-20-260-52419)
FAX:  [91-20-261-25432](tel:011-91-20-261-25432)

E-mail: [vidyamave@gmail.com](mailto:vidyamave@gmail.com)

Field Representative

Hannah Edmondson, RN, BSN

Keck School of Medicine
University of Southern California

5P21/ Rand Schrader Clinic
1300 N Mission Road, Room 349
Los Angeles CA 90033

Phone: 323-343-8282

FAX: 323-226-2083

E-Mail: [edmondso@usc.edu](mailto:edmondso@usc.edu)

Laboratory Technologist

Carmen M. Irizarry, MT (ASCP)

Puerto Rico-AIDS CRS

UPR Medical Science Campur

Pathology

Suite 606-A

P.O. Box 365067

San Juan, PR 00936-5067

Phone: 787-758-5815

FAX: 787-758-5815

E-mail: [rcmlab053@rcm.upr.edu](mailto:rcmlab053@rcm.upr.edu)

CSS Representative

Amina Shali

Clinical Trials Unit
P.O. Box 2066
Eldoret 30100
KENYA

Phone: 254 720 858 553

E-Mail: [aminashali@yahoo.co.uk](mailto:aminashali@yahoo.co.uk)

Laboratory Data Coordinator

*Travis Behm, BS*

*Frontier Science and Technology Research Foundation*

*4033 Maple Road*

*Amherst, NY 14228*

*Phone: 716-834-0900, ext. 7377*

*FAX: 716-833-0655*

*E-Mail:* [*tbehm@fstrf.org*](mailto:tbehm@fstrf.org)

International Program Specialist

Christina Blanchard Horan, PhD, MA, CCRP

ACTG Operations Center

Social & Scientific Systems, Inc.

8757 Georgia Avenue, 12^th^ Floor

Silver Spring, MD 20910-3714

Phone: 301-628-3339

FAX: 301-628-3302

E-Mail: [cblanchardhoran@s-3.com](mailto:cblanchardhoran@s-3.com)

# STUDY MANAGEMENT

This section provides important instructions about how sites should attempt to have questions related to A5225/HiFLAC answered. Following these instructions will help ensure that you receive an answer as quickly as possible. Please read through all sections carefully before sending an e-mail message.

General Questions

All questions concerning this protocol should be sent via e-mail to [**actg.corea5225@fstrf.org**](mailto:actg.corea5225@fstrf.org). Sites must not send study-related inquiries to individual members of the team. Sites should include the protocol identifier (A5225/HiFLAC) in the subject line of all study-related e-mail messages. The appropriate core team member will respond via e-mail. A response should generally be received within 24 hours (Monday-Friday).

Protocol E-Mail Group

Sites that are interested in participating in this study should immediately contact the Computer Support Group at the Data Management Center via e-mail ([actg.user.support@fstrf.org](mailto:actg.user.support@fstrf.org)) to have the relevant personnel at the site added to the actg.protA5225 e-mail group as soon as possible. Inclusion in the protocol e-mail group will ensure that site staff receive important information about the study throughout its implementation.

Clinical Medical Management

For questions concerning clinical medical management, including entry criteria, toxicity management, concomitant medications, progression of symptoms, and coenrollment, contact the core team ([**actg.corea5225@fstrf.org**](mailto:actg.corea5225@fstrf.org)).

- Include the protocol number, patient identification number (PID), and a brief relevant history.

Pharmacologic, Neurological, and Functional Assessments

For questions specifically related to pharmacologic or neurological and functional assessments, contact the protocol pharmacologist (Amanda Corbett) or neurologist (David Clifford), respectively, through the core team logon ([actg.corea5225@fstrf.org](mailto:actg.corea5225@fstrf.org)).

Nonclinical Questions

For nonclinical questions about transfers, the case report forms (CRF), CRF schedule of events, randomization or registration, transfers, delinquencies, and other data management issues, contact the data manager.

- For transfers, reference the Patient Transfer from Site to Site SOP 119, and contact Jennifer Janik directly ([janik@fstrf.org](mailto:janik@fstrf.org)).
- For other questions, send an e-mail message the study’s data manager (Jennifer Janik) either directly or through the core team logon ([actg.corea5225@fstrf.org](mailto:actg.corea5225@fstrf.org)).
- Include the protocol number and PID in the subject line.

Randomization Questions

For randomization questions or problems, or study identification (SID) lists, contact the SDAC/DMC programmers.

- Call the SDAC/DMC Randomization Desk at 716-898-7301.

Or

- E-mail [rando.support@fstrf.org](mailto:rando.support@fstrf.org).

Non-US CRSs may need to perform manual enrollment in the event of local internet, network, or telephone problems.

Computer and Screen Problems

Contact the SDAC/DMC programmers.

- Send an e-mail message to [actg.support@fstrf.org](mailto:actg.support@fstrf.org)

Or

- Call 716-834-0900, ext. 7302.

Protocol Document Questions

For protocol document questions, contact the Clinical Trials Specialist.

- Send an e-mail message to Evelyn Hogg ([ehogg@s-3.com](mailto:ehogg@s-3.com)) and cc the core team ([actg.corea5225@fstrf.org](mailto:actg.corea5225@fstrf.org)).

Copies of the Protocol

To request copies of the protocol:

- A single hard copy: Send an e-mail message to [**NCCOps@s-3.com**](mailto:NCCOps@s-3.com) (ATTN: Diane Delgado).
- Electronic copies can be downloaded from the Members area of the ACTG Web site ([https://www.actgnetwork.org](https://www.actgnetwork.org/)).

Study Product Information

To request a copy of study product package insert:

- Send an e-mail message to the DAIDS Regulatory Support Center (RSC) at [RIC@tech-res.com](mailto:RIC@tech-res.com)

Or

- Call 301-897-1708.

Protocol Registration

- Send an e-mail message to [Protocol@tech-res.com](mailto:Protocol@tech-res.com)

or

- Call 301-897-1707

Study Drug Questions

For questions or problems regarding study drug, dose, supplies, records, and returns, contact Lynette Purdue, Protocol Pharmacist.

- Call 301-496-8213.

Or

- Send an e-mail message to: [lpurdue@niaid.nih.gov](mailto:lpurdue@niaid.nih.gov).

IND (Investigational New Drug) Number or questions concerning IND

- Send an e-mail message to [regulatory@tech-res.com](mailto:regulatory@tech-res.com)

Or

- Call 301-897-1706.

Ordering study drug

Contact the Clinical Research Products Management Center (CRPMC).

- Call 301-294-0741.

Expedited Adverse Event (EAE) Reporting/Questions

Contact DAIDS through the RSC Safety Office:

- Send an e-mail to [DAIDSRSCSafetyOffice@tech-res.com](mailto:DAIDSRSCSafetyOffice@tech-res.com)

Or

- Call 800-537-9979 or 301-897-1709

Or

- Fax 800-275-7619 or 301-897-1710.

Phone Calls

Sites are responsible for documenting any phone calls made to protocol team members. Send an email to [actg.corea5225@fstrg.org](mailto:actg.corea5225@fstrg.org).

Protocol-Specific Web Page

Additional information concerning study management of ACTG studies can be found on the ACTG Web page. Information about this study can be found on its protocol-specific web page (PSWP) ([*https://www.actgnetwork.org*](https://www.actgnetwork.org/protocols)).

# GLOSSARY OF STUDY-SPECIFIC TERMS

##### 5FC 5-fluorocytosine

ampho B amphotericin B deoxycholate (in A5225/HiFLAC, the abbreviated name is being used for convenience and is not intended to represent an official name for the drug)

ampho B-based regimen in this study, a regimen that is either ampho B alone or ampho B in combination with 5-fluorocytosine **or fluconazole, according to local standard of care**

CCTG California Collaborative Treatment Group

CM cryptococcal meningitis

CNS central nervous system

CSF cerebrospinal fluid

DLT dose-limiting toxicity: interruption of an induction dose of study-provided fluconazole (i.e., 1200 mg, 1600 mg, or 2000 mg daily) for at least 3 consecutive days as the result of a fluconazole-related toxicity, where the discontinuation was initiated prior to day 14

ECG electrocardiogram

EFV efavirenz

hERG human ether-a-go-go-related gene

HiFLAC High-Dose Fluconazole for Treatment in AIDS-Associated Cryptococcal Meningitis, acronym for A5225

IRIS immune reconstitution inflammatory syndrome

LFCrAg lateral flow cryptococcal antigen (assay)

LP lumbar puncture

MOPS manual of operations

MTD maximum tolerated dose, defined for A5225/HiFLAC as the highest dose of fluconazole that can be safely used [i.e., a dose that results in a ≤ 25% rate of fluconazole dose-limiting toxicity (DLT)].

PML progressive multifocal leukoencephalitis

SIP site implementation plan

SMC study monitoring committee

SoC standard of care

SoE schedule of events (protocol section 6.1)

TEN toxic epidermal necrolysis

TLT treatment-limiting toxicity: a toxicity that results in discontinuation of ampho B-based treatment prior to day 14 and prior to receipt of a total of 8.4 mg/kg ampho B

# SCHEMA

A5225/HiFLAC

A Phase I/II Dose-Finding Study of High-Dose Fluconazole Treatment in AIDS-Associated Cryptococcal Meningitis

DESIGN Overview

A5225/HiFLAC is a phase I/II dose escalation and validation study of the safety, tolerability, and therapeutic effect of an induction-consolidation strategy of high-dose fluconazole alone **or with oral flucytosine (5FC)** for the treatment of cryptococcal meningitis (CM) in HIV-infected participants. As noted in section 2.2, comparison of this study’s results with historical data may be problematic. For this reason, a cohort treated with amphotericin B deoxycholate (which will be referred to as ‘ampho B’ in this study) alone or in combination with 5-fluorocytosine **or fluconazole, according to local standard of care** (**any** of which will be referred to as ‘an ampho B-based regimen’ in this document) will be included in this study.

The study will proceed in two stages (see A5225/HiFLAC Stages 1 & 2 Flow Chart). In Stage 1, Dose Escalation, up to three induction doses of fluconazole **alone** will be tested in sequentially enrolled cohorts. Stage 2, Dose Validation, will not open until the maximum tolerated dose (MTD) of fluconazole has been identified in Stage 1. In Stage 2, induction doses of fluconazole that are found to be safe in Stage 1 will be tested in simultaneously enrolled cohorts. In each stage, participants will be randomized at entry into Step 1. Over the course of the study, participants will register to subsequent steps (Steps 2-4) based on their initial randomization and/or their response to treatment. The study steps are:

- Step 1: Induction therapy with either high dose fluconazole or ampho B
- Step 2: Induction following early ampho B intolerance (only for participants randomized to ampho B treatment in Step 1) (fluconazole at 400-800 mg daily)
- Step 3: Consolidation therapy (fluconazole 400 mg daily)
- Step 4: Maintenance therapy (fluconazole 200 mg daily)

Stage 1 (Dose Escalation)

Stage 1 will include up to three sequentially enrolled cohort sets, each testing a different induction dose of fluconazole and gathering data about ampho B-based treatment (see A5225/HiFLAC Stage 1 Flow Charts #1-3). Each cohort will enroll up to 24 participants randomized to fluconazole and up to 8 participants randomized to an ampho B-based regimen. At any time during the study, enrollment may be halted for safety reasons.

- Cohort Set 1: fluconazole 1200 mg daily or ampho B-based regimen. Once the safety data for the last participant enrolled in Cohort 1 has been reported, reviewed, and found to be acceptable, Cohort 2 may be opened. Cohort 1 was opened to accrual on **x/x/x, and closed to accrual on x/x/2011**.
- Cohort Set 2: fluconazole 1600 mg daily or ampho B-based regimen. Once the safety data for the last participant enrolled in Cohort 2 has been reported, reviewed, and found to be acceptable, Cohort 3 may be opened. **Cohort 2 was opened to accrual on 10/7/2011 and closed to accrual on 6/x/2012.**
- Cohort Set 3: fluconazole 2000 mg daily or ampho B-based regimen. **Cohort 3 was opened to accrual on 7/x/2012 and closed to accrual on 8/x/2013.**

Fluconazole

Participants randomized to receive fluconazole will enter Step 1 and receive the assigned induction dose, adjusted for weight, for a minimum of 4 weeks and a maximum of 10 weeks.

In addition to the cerebrospinal fluid (CSF) samples collected at screening or entry, CSF samples will be collected at week 2 and every 2 weeks through week 10 until a negative culture is obtained. If a negative culture is obtained prior to week 10 (i.e., negative culture results are available at or before week 8 from a sample collected at least 2 weeks previously), the participant will enter Step 3 and will receive fluconazole 400 mg daily [or 600 mg daily if receiving rifampin (RIF)] until week 10. It is possible that a participant will not enter Step 3.

At week 10, all participants will enter Step 4 and receive fluconazole 200 mg daily through week 24. Note that participants randomized to fluconazole will not enter Step 2, which is described below.

Ampho B

Participants randomized to receive an ampho B-based regimen in Step 1 will receive amphotericin B deoxycholate at a dose of 0.7-1.0 mg/kg daily as part of the local standard of care (SoC) for approximately 2 weeks. At each site, the daily ampho B dose will be the same for each cohort.

In addition to the CSF samples collected for culture at screening or entry, CSF samples will be collected for culture at week 2 and again every 2 weeks through week 10 until a negative culture is obtained.

Participants who experience a treatment limiting toxicity (TLT), defined as a toxicity to ampho B treatment that results in discontinuation of that treatment prior to day 14 and prior to receipt of a total of 8.4 mg/kg ampho B, may either remain in Step 1 and receive an alternative treatment, such as liposomal ampho B, if available, or may enter Step 2 and receive study-provided fluconazole 800 mg or the maximum locally approved daily dose.

Participants who complete Step 1 will enter Step 3 after approximately 2 weeks.

For participants in Step 2, if negative CSF culture results are obtained prior to week 10 (i.e., negative culture results are available at or before week 8 from a sample collected at least 2 weeks previously), the participant will enter Step 3 and will receive fluconazole 400 mg daily (or 600 mg daily if receiving RIF) until week 10. It is possible that participants in Step 2 will not enter Step 3.

At week 10, all participants will enter Step 4 and receive fluconazole 200 mg daily through week 24.

Stage 2 (Dose Validation)

In Stage 1, when either the MTD of fluconazole is reached or the week 10 visit for the final participant randomized to the 2000 mg/day cohort is completed, all data through week 10 on all participants will be reviewed; the protocol will be amended before Stage 2 (Dose Validation) of the study opens. In Stage 2, participants will be randomized to receive an induction treatment of either an ampho B-based regimen or one of several doses of fluconazole **or fluconazole plus 5FC** at or below the MTD identified in Stage 1 (see A5225/HiFLAC Stage 2 Flow Chart).

Stage 1 and Stage 2 Evaluations

Participants will be monitored for safety and toxicity throughout the study period.

The primary efficacy outcome measure is the change in quantitative CSF culture for Cryptococcus at week 2. At screening or entry and again at week 2, CSF samples for culture will be collected from all participants. Additional CSF samples will be collected and cultured from participants on study treatment at weeks 4, 6, 8, and 10, as necessary until a negative culture is obtained. Treatment failure for any participant is defined as a positive culture from a CSF sample collected at week 10 and that is read by or at week 12.

Pharmacokinetic (PK) samples will be obtained at specified time points from all participants enrolled in fluconazole induction cohorts (see section 10.0 for sampling information).

DURATION 24 weeks

SAMPLE SIZE Up to 192 participants (up to 96 in Stage 1 and up to 96 in Stage 2).

POPULATION HIV-infected males and females ≥16 years of age presenting with their first episode of CM.

REGIMEN

Table 1: Stage 1 Regimens

| Cohort | N | Induction Step (Step1)  Daily Dose | Induction Following Ampho B Intolerance Step  (Step 2) | Consolidation Step (Step 3)  Daily Fluconazole Dose | Maintenance Step (Step 4) Daily  Fluconazole Dose |
| --- | --- | --- | --- | --- | --- |
| 1A^1^ | 24 | Fluconazole  1200 mg | N/A | 400 mg  Or 600 mg with RIF | 200 mg |
| 1B^1^ | 8 | Ampho B-based regimen;  0.7-1.0 mg/kg ampho B | Fluconazole  400-800 mg daily | 400 mg  Or 600 mg with RIF | 200 mg |
| 2A | 24 | Fluconazole  1600 mg | N/A | 400 mg  Or 600 mg with RIF | 200 mg |
| 2B | 8 | Ampho B-based regimen;  0.7-1.0 mg/kg ampho B | Fluconazole  800 mg daily or maximum locally approved dose | 400 mg  Or 600 mg with RIF | 200 mg |
| 3A | 24 | Fluconazole  2000 mg | N/A | 400 mg  Or 600 mg with RIF | 200 mg |
| 3B | 8 | Ampho B-based regimen;  0.7-1.0 mg/kg ampho B | Fluconazole  800 mg daily or maximum locally approved dose | 400 mg  Or 600 mg with RIF | 200 mg |

^1^ Enrollment to **Stage 1 ended on x/x/2013**.

Fluconazole dose adjustments based on weight must be implemented at the first dose; after entry, fluconazole dose adjustments in response to toxicities should be implemented on a case-by-case basis (see section 7.4).

Table 2: Stage 2 Regimens

| Cohort | N | Induction Step (Step1)  Daily Dose | Induction Following Ampho B Intolerance Step (Step 2)  Daily Dose | Consolidation Step (Step 3)  Daily Fluconazole Dose | Maintenance Step (Step 4) Daily  Fluconazole Dose |
| --- | --- | --- | --- | --- | --- |
| 4 | 24 | Ampho B-based regimen;  0.7-1.0 mg/kg ampho B | Fluconazole 800 mg daily or maximum locally approved dose | 400 mg  Or 600 mg with RIF | 200 mg |
| 5 | 24 | Fluconazole **1600 mg** | N/A | 400 mg  Or 600 mg with RIF | 200 mg |
| 6 | 24 | Fluconazole **2000 mg** | N/A | 400 mg  Or 600 mg with RIF | 200 mg |
| 7 | 24 | Fluconazole **2000 mg plus Flucytosine xx mg** | N/A | 400 mg  Or 600 mg with RIF | 200 mg |

Fluconazole dose adjustments based on weight must be implemented at the first dose; after entry, fluconazole dose adjustments in response to toxicities should be implemented on a case-by-case basis (see section 7.4). **Note that these dose adjustments are recommended only for study-provided fluconazole.**

A5225 Stages 1 and 2 Overview

Flow Chart

Study opens (Stage 1)

Cohorts 1A and 1B open; 32 participants are randomized 3:1 to an induction step of either fluconazole (at 1200 mg/day) or ampho B-based regimen.

**Cohort 1 was closed after data from the first 29 participants were reviewed.**

Cohorts 2A and 2B **opened to accrual on Friday, October 7, 2011.** **Up to** 32 participants **will be** randomized 3:1 to an induction step of either fluconazole (at 1600 mg/day) or ampho B-based regimen.

.

**Data through week 2 from all participants in Cohort 2 will be reviewed before a decision about opening Cohort 3 is made. If safe to continue, Cohrt 3 will be opened.**

**Up to** 32 participants **will be enrolled in** Cohorts 3A and 3B **and** randomized 3:1 to an induction step of either fluconazole (at 2000 mg/day) or ampho B-based regimen.

.

**After data through week 10 from all participants in the last Stage 1 cohort are available, a r**eview of **all available** Stage 1 data **will occur**; MTD of fluconazole **will be** determined; relationship of fluconazole dose to efficacy **will be** examined; determination of doses to be tested in Stage 2 **will be** made.

Stage 2 **will open**; **approximately** 96 participants **will be** randomized to receive 1 of up to 3 fluconazole doses **or fluconazole plus 5FC** or to receive ampho B-based regimen in the induction step.

Protocol **will be** amended.

A5225/HiFLAC Stage 1 Flow Chart #1

Randomization

Step 3:

400 mg fluconazole

(or 600 mg w/ RIF)

Step 3:

400 fluconazole

(or 600 mg w/ RIF)

Step 2:

400-800 mg **daily**

fluconazole

Cohort 1

Step 1:

1200 mg fluconazole

4-10 wks

Step 4:

200 mg

fluconazole through wk 24

Step 1:

ampho B based regimen

(~ 2 wks)

Step 4:

200 mg

fluconazole through wk 24

if CSF-neg.

by wk 8

at wk 10

if CSF neg.

by wk 8

**Cohort 1 was closed after data from the first 29 participants were reviewed.**

if TLT before wk 2

at week 10

at week 10

at wk 10

at week 2

A5225/HiFLAC Stage 1 Flow Chart #2

Cohort 2

Randomization

**(Cohort 2 was opened to accrual on Friday, October 7, 2011.)**

Step 1:

1600 mg fluconazole

4-10 wks

Step 3:

400 mg fluconazole

(or 600 mg w/ RIF)

Step 4:

200 mg

fluconazole through wk 24

Step 1:

ampho B based regimen

(~ 2 wks)

Step 3:

400 mg fluconazole

(or 600 mg w/ RIF)

Step 4:

200 mg

fluconazole through wk 24

Step 2:

800 mg **daily**

fluconazole **or maximum locally approved dose**

Week 2

if TLT before wk 2

at wk 10

if CSF neg.

by wk 8

at week 10

at wk 10

if CSF neg.

by wk 8

at week 2

at week 10

If / when safe to continue, Cohort 3 may open.

A5225/HiFLAC Stage 1 Flow Chart #3

Cohort 3

Randomization

Step 1:

2000 mg fluconazole

4-10 wks

Step 3:

400 mg fluconazole

(or 600 mg w/RIF)

Step 4:

200 mg

fluconazole through wk 24

Step 1:

ampho B based reg.

(~ 2 wks)

Step 3:

400 mg fluconazole

(or 600 mg w/ RIF)

Step 4:

200 mg

fluconazole through wk 24

Step 2:

800 mg **daily**

fluconazole **or maximum locally approved dose**

if TLT before wk 2

at wk 10

if CSF neg.

by wk 8

at week 10

at wk 10

if CSF neg.

by wk 8

at week 2

at week 10

A5225/HiFLAC Stage 2 Flow Chart

at week 2

at week 10

Randomization

Step 1:

***1-3***

fluconazole cohorts

(4-10 wks)

Step 4:

200 mg fluconazole through wk 24

Step 3:

400 mg fluconazole (or 600 mg w/ RIF)

Step 1: ampho B based reg.

(~ 2 wks)

Step 2:

800 mg daily

fluconazole or maximum locally approved dose

Step 3:

400 mg fluconazole

(or 600 mg w/ RIF)

Step 4:

200 mg

Flucon-azole through wk 24

if TLT before wk 2

at wk 10

if CSF neg.

by wk 8

if CSF-neg by wk 8

at wk 10

at week 10

## 1.0 HYPOTHESES AND STUDY OBJECTIVES

### 1.1 Hypotheses

1.1.1 Fluconazole alone at a dose of 1200, 1600, and/or 2000 mg/day will be well tolerated and safe in participants presenting with HIV-associated CM.

1.1.2 Increasing doses of fluconazole will have increasing efficacy, as follows:

1.1.2.1 Increasing microbiological (fungicidal) efficacy as measured by the mean change between dosing groups in log_10_ concentration of cryptococci in CSF between baseline and week 2.

1.1.2.2 Increasing clinical efficacy as measured by survival at 10 weeks.

### 1.2 Primary Objectives

1.2.1 To assess the MTD of fluconazole by comparing the safety and tolerability of induction regimens of 1200, 1600, and 2000 mg/day.

1.2.2 To determine whether the safety and efficacy of high induction-dose fluconazole therapy support development of a phase II/III trial, and, if so, to select the most appropriate fluconazole dose regimen (based on safety and efficacy data from this study) to be evaluated in that trial.

### 1.3 Secondary Objectives

- - 1. To compare the clinical and fungicidal effects of different induction regimens of fluconazole during induction at weeks 2, 4, 6, 8, and 10.
    2. To assess functional status (prior to onset of current CM illness, at study entry, at week 10, and at premature treatment or study discontinuation) and to assess neurological status [at entry, weeks 2, 10, 24, and at progression of symptoms and premature treatment or study discontinuation].

1.3.3 To assess the time to death in each cohort and the combined rates of DLT and death.

1.3.4 To describe the effect of baseline clinical, neurological, and mycological characteristics on clinical and mycological success at week 10 and on time to CSF sterility.

- - 1. To assess the length of hospitalization between study entry and hospital discharge and for any subsequent readmission for the treatment of CM.
    2. To assess the incidence of progression of symptoms and its components among all participants receiving ART during the study.

1.3.7 To estimate the tolerability, toxicity, and efficacy of an ampho B-based regimen in the setting of non-U.S. sites for the purposes of planning additional studies.

### 1.4 Exploratory Objectives

1.4.1 To assess the relationship of antifungal drug susceptibility of cryptococcal isolates to fluconazole and microbiological and clinical outcomes.

1.4.2 To assess the pharmacology [pharmacokinetics (PK), pharmacodynamics (PD)], and toxicity of high-dose fluconazole and, if samples are available, relationships with concomitant RIF and/or efavirenz (EFV) (see section 10.1 for specific pharmacology objectives].

1.4.3 To evaluate a lateral flow cryptococcal antigen (LFCrAg) detection assay using urine, serum, plasma, and CSF samples from participants with confirmed CM.

## 2.0 INTRODUCTION

### 2.1 Background

Cryptococcal meningitis (CM) is the most common central nervous system (CNS) complication of AIDS worldwide and accounts for up to 1/3 of all deaths from AIDS in many parts of the developing world [Pitisuttithum, 2001; Mwaba, 2001; Ford, 2000; Gangaidzo, 1999]. With the dissemination of antiretroviral therapy (ART), which provides the possibility of long-term survival after diagnosis of CM, development of practical CM therapy has become a high priority. Treatment of CM is problematic in locations with limited resources: treatment with an ampho B-based regimen requires maintaining intravenous (IV) access, monitoring and treating acute infusion reactions, and monitoring of common, cumulative treatment-limiting toxicities (TLTs) such as renal dysfunction and electrolyte imbalance (particularly low K+ and Mg++). In addition, the price of ampho B is often prohibitive. Thus, costs of ampho B, its administration, and the requisite safety monitoring often preclude its use in resource-limited settings, leaving patients untreated for CM or treated with inadequately effective low-dose regimens of fluconazole only.

Because fluconazole is widely available, inexpensive, can be given orally, has a demonstrated safety profile over a broad range of doses, and has proven activity against *Cryptococcus neoformans*, the use of fluconazole to treat CM is quite appealing. Even in developed countries, the use of high-dose fluconazole as an alternative to ampho B-based regimens for severe cases of CM would also be desirable. Elimination of the requirement for the two weeks of daily IV infusions of ampho B that is currently recommended for induction therapy of CM would likely reduce the number and degree of TLTs. In addition, the use of high-dose fluconazole alone would prove cheaper and far more convenient.

At the most common fluconazole dose of 200-400 mg/day, however, overall clinical success is expected in only 34 to 42% of patients [Larsen, 1990; Saag, 1992]. When fluconazole at 400 mg/day is combined with flucytosine, overall success is improved [Larsen, 1994] and is comparable to that of ampho B followed by fluconazole [van der Horst, 1997]. The median cost of ampho B is $6.73 (U.S.) per 50-mg vial; fluconazole is $0.20 per 200-mg capsule and $0.82 per 200-mg vial [World Health Organization (WHO) drug pricing information 2004]. Thus, fluconazole at 2000 mg/day (the highest dose proposed in this study) is comparable in price to ampho B, even if some doses must be provided by IV infusion.

2.1.1 Fluconazole

Fluconazole is a synthetic triazole antifungal agent. It is a highly selective inhibitor of fungal cytochrome P450-dependent sterol C-14 alpha-demethylation. The subsequent loss of normal sterols correlates with the accumulation of 14 alpha-methyl sterols in fungi and may be responsible for the fungistatic activity of fluconazole.

The pharmacology of fluconazole has been well studied only at doses of 50-400 mg per day. It is well absorbed orally (~ 90%), with peak plasma concentrations reached within 1-3 hours after dosing, and is not affected by food. Peak plasma concentrations at steady state on daily dosing are usually about twice those at trough. Fluconazole is minimally protein bound, widely distributed in body water with an apparent volume of 0.7 L/kg, and 80% is excreted unchanged in the urine with a half-life of 25-30 hours. About 11% is excreted in the urine as the glucuronide and N-oxide metabolites, which are both inactive and probably come from metabolism in the liver by cytochrome P450 3A4 enzymes. Renal insufficiency markedly prolongs the excretion of the drug.

Steady state is reached in plasma in 5-10 days on daily dosing, with steady state plasma concentrations of 4.6 and 9 mg/L after 200 and 400 mg doses, respectively. Cerebrospinal fluid (CSF) levels are 50-90% of those in serum [Foulds, 1988]. Over the range of 100–400 mg orally, the variation of fluconazole maximum plasma concentration (C_max_) and area under the concentration–time curve (AUC) are proportional to the administered dose. The elimination half-life and the time of C_max_ also remain constant in this dosing range. This indicates the linearity of the pharmacokinetics (PK) of fluconazole for oral doses of 50-400 mg [Tett, 1995; data on file with Pfizer].

Adrenal insufficiency (AI) could be an issue in A5225 participants for three reasons: 1) AI is considered an uncommon complication of destruction of the glands by opportunistic infections (especially cytomegalovirus infection), neoplasms, or other conditions complicating advanced AIDS. Massive damage is required to induce AI since adequate function can be maintained by only 10% of the glands; 2) AI associated with critical illness is caused by both failure of corticosteroid production and tissue corticosteroid resistance and is characterized by exaggerated and protracted pro-inflammatory responses; 3) AI may be induced by high dose fluconazole possibly by toxicity to the p-450 systems of adrenal cells, similar to that recognized for ketoconazole, but induced by ketoconazole at concentrations 100-fold less than those required for fluconazole in a rat model.

Two small studies have prospectively evaluated the prevalence of AI in HIV patients. In 25 well (n=9) or critically ill (n=16) patients, 5 (19%) had AI by criteria of stress cortisol < 18 [Prasanthai 2007]. Fourteen of 28 (50%) critically ill patients were diagnosed with AI in another study by these same criteria [Marik 2002].

Four of the 854 (prevalence of 0.47% or 1/200) patients who were seen over 9 years in Taiwan were found to have AI, three by criteria of low cortisol and/or high ACTH levels or inadequate responses to ACTH stimulation. The fourth patient was on 800 mg of fluconazole for 68 days and was suspected, but not proven, to have AI, based on symptoms and other non-specific laboratory values which resolved after discontinuation of fluconazole. This low rate is consistent with the large reserve of the gland [[Huang](http://www.ncbi.nlm.nih.gov/sites/entrez?Db=pubmed&Cmd=Search&Term="Huang YW"%5BAuthor%5D&itool=EntrezSystem2.PEntrez.Pubmed.Pubmed_ResultsPanel.Pubmed_DiscoveryPanel.Pubmed_RVAbstractPlus) 2004].

To date, QT interval prolongation has been demonstrated with azole antifungal agents, including ketoconazole, itraconazole and fluconazole [Khazan 2002, Roden 2004, Gandhi 2003, Tholakanahalli 2001, Pham 2006, Esch 2008, Gupta 2007, Kannankeril 2007]. Post-marketing surveillance reports have documented rare cases of QT prolongation and torsade de pointes in patients receiving fluconazole, generally in seriously ill patients with multiple confounding risk factors [Diflucan 2011 package insert, Wassmann 1999, Dorsey 2000]. Azoles may cause prolongation of the QT interval either directly or by inhibiting the hepatic metabolism of other QT-prolonging agents. Ketoconazole is known to block the human ether-a-go-go-related gene (hERG) product, and thereby inhibit the potassium Ikr current in the heart [Takemasa 2008]. Whether fluconazole also inhibits hERG is currently unknown. However, all azoles inhibit cytochrome 3A4 to some extent, although fluconazole is a relatively weak inhibitor of this enzyme. The ICH E14 Guidance Document for Industry helps in assessing clinical evaluation of QT/QTc interval prolongation and proarrhythmic potential for non-antiarrhythmic drugs [International Conference on Harmonisation, 2005].

Toxicity of fluconazole is infrequent at low doses, and that observed at high doses in a phase II clinical trial conducted in the U.S. by the California Collaborative Treatment Group (CCTG) is discussed below. Patterns of abnormal aspartate aminotransferase (AST), alanine aminotransferase (ALT), alkaline phosphatase, gamma-glutamyl transferase (GGT), and bilirubin values did not suggest that fluconazole treatment was associated with an increased risk for hepatotoxicity, although a marked elevation of some of theses enzymes occurred in approximately 1% of patients.

The CCTG trial demonstrated improved clinical outcomes with increasing doses of fluconazole [Milefchik, 2008]. The composite 10-week overall response rates (success defined as alive and CSF culture negative) for fluconazole with and without flucytosine are shown in Figure 1. Of note, fluconazole alone for 10 weeks at the highest doses (1600 mg and 2000 mg/day) had clinical success rates over 60%, comparable to the overall success seen when ampho B is used for 2 weeks followed by fluconazole at 400 mg daily. As the CCTG trial was small and principally designed to assess the safety of higher dose fluconazole with and without flucytosine, a larger phase II trial is needed to confirm these findings in multiple settings and to assess the potential of an all oral treatment regimen to supplant the standard ampho B-based regimen.

Higher doses of fluconazole have been safely employed in the treatment of CM [Haubrich 1994], histoplasmosis [McKinsey 1996], candidemia [Rex 2003], and coccidioidomycosis [Galgiani 1993]. When higher dose fluconazole (up to 2000 mg daily) has been used alone, few serious toxic side effects have been observed (granulocytopenia 4%, and increased liver enzyme test 2%; see Table 2-1).

Table 2-1: Summary of Toxicity Events, CCTG Trial

| Toxicity | Fluconazole alone  N (%) | Fluconazole plus Flucytosine  N (%) |
| --- | --- | --- |
| Sweating | 6 (12%) | 6 (15%) |
| Tachycardia | 7 (14%) | 7 (18%) |
| Abdominal pain | 8 (16%) | 5 (12%) |
| Constipation | 8 (16%) | 6 (15%) |
| Diarrhea | 10 (20%) | 7 (18%) |
| Nausea | 29 (59%) | 23 (57%) |
| Vomiting | 27 (55%) | 24 (60%) |
| Weight loss | 9 (18%) | 8 (20%) |
| Anemia | 7 (14%) | 1 (3%) |
| Granulocytopenia (< 500) | 2 (4%) | 7 (18%) |
| Thrombocytopenia (<50K) | 0 | 3 (7%) |
| Hepatic enzyme increase (1.5x) | 1 (2%) | 3 (7%) |
| Alopecia | 6 (12%) | 4 (10%) |
| Dry skin | 9 (18%) | 13 (32%) |
| Increased skin pigmentation | 1 (2%) | 1 (3%) |
| Decreased hearing | 3 (6%) | 5 (12%) |

When higher dose fluconazole has been combined with flucytosine, the expected hematological side effects (granulocytopenia 18% and thrombocytopenia 7%) were easily detected by regular monitoring. In the CCTG trial, doses of fluconazole at 800, 1200, 1600, and 2000 mg with and without flucytosine were evaluated. Four dosing cohorts were employed, with participants randomly allocated between treatment cells. The baseline demographics of that study population are presented in Table 2-2.

Cohort 1: fluconazole @ 800 mg with/without flucytosine and 1200 mg of fluconazole alone.

Cohort 2: fluconazole @ 1200 mg with/without flucytosine and 1600 mg fluconazole alone.

Cohort 3: fluconazole @ 1600 mg with or without flucytosine.

Cohort 4: fluconazole @ 2000 mg with or without flucytosine.

A trial of fluconazole at 10 mg/kg/day (equivalent to about 800 mg) for candidemia noted elevations in liver enzymes in 14 of 30 patients, but clear attribution to fluconazole toxicity in this setting is not possible [Graninger 1993].

Adverse events related to fluconazole at 800 mg daily were reported by 48% of patients in a multicenter, randomized, open-label study to determine the efficacy and safety of two different daily doses of fluconazole (400 and 800 mg) in the treatment of non-life-threatening blastomycosis. One patient of 20 who received 800 mg daily presented a moderate elevation of liver transaminase level [Pappas 1997].

Another trial of fluconazole was conducted in cancer patients with presumed or proven mold infection. Groups of patients received fluconazole at four dosages (800, 1200, 1600, or 2000 mg/day). Adverse events, plasma levels, and clinical response were examined. Eight of 39 evaluable patients had elevated liver function test results that were either possibly or probably related to fluconazole [Anaissie 1995].

In addition to these studies, a few published reports in the past 20 years have shown severe fluconazole-induced hepatotoxicity despite treatment of cocciciodomycosis and blastomycosis at doses of 800 mg/day [[Crerar-Gilbert](http://www.ncbi.nlm.nih.gov/sites/entrez?Db=pubmed&Cmd=Search&Term="Crerar-Gilbert A"%5BAuthor%5D&itool=EntrezSystem2.PEntrez.Pubmed.Pubmed_ResultsPanel.Pubmed_DiscoveryPanel.Pubmed_RVAbstractPlus)  1999; [Jacobson 1994.]](http://www.ncbi.nlm.nih.gov/sites/entrez?Db=pubmed&Cmd=Search&Term="Jacobson MA"%5BAuthor%5D&itool=EntrezSystem2.PEntrez.Pubmed.Pubmed_ResultsPanel.Pubmed_DiscoveryPanel.Pubmed_RVAbstractPlus) For example, an 81-year old patient with renal impairment developed fulminant hepatic necrosis within 10 days of fluconazole 400 mg daily when his fluconazole serum level was 982 ug/mL (normal range 6-15 ug/mL) and without exposure to other potentially hepatotoxic drugs. These levels are clearly 10-fold higher than those expected and result from the effects of either renal failure or potentially from unrecognized dosing errors [Bronstein 1997].

Table 2-2: Demographic Information, CCTG Trial

|  | Fluconazole Alone | | | | Fluconazole plus Flucytosine | | | |
| --- | --- | --- | --- | --- | --- | --- | --- | --- |
| Dose | 800 | 1200 | 1600 | 2000 | 800 | 1200 | 1600 | 2000 |
| Volunteers | 9 | 16 | 16 | 8 | 8 | 8 | 16 | 8 |
| Male | 100% | 94% | 94% | 100% | 100% | 100% | 100% | 100% |
| Age | 35 | 40 | 37 | 36 | 38 | 34 | 37 | 37 |
| CD4 Cells | 8 | 36 | 33 | 33 | 25 | 29 | 11 | 47 |
| CSF Ag. | 1:1024 | 1:128 | 1:64 | 1:32 | 1:512 | 1:64 | 1:512 | 1:256 |
| Min. Ag. | 1:2 | Neg | Neg | Undil | 1:2 | Neg | 1:2 | 1:2 |
| Max. Ag. | 1:8192 | 1:65536 | 1:8192 | 1:4096 | 1:131072 | 1:32768 | 1:32768 | 1:8192 |
| 1^st^ O.I. | 60% | 31% | 44% | 87% | 37% | 63% | 44% | 25% |

Overall, fluconazole was well tolerated up to doses of 2000 mg daily (Table 2-1). The most commonly reported adverse events (AEs) were stomach pain, nausea, and vomiting. One participant withdrew consent after three episodes of vomiting. Two participants developed cranial venous thrombosis of uncertain association with the higher fluconazole doses (800 and 1200 mg) without concurrent flucytosine use. One participant taking 2000 mg of fluconazole alone bled to death from an interaction with warfarin. Hair loss and darkening of skin color were observed in a limited number of participants at doses of 1200 mg and higher. Liver function abnormalities were uncommon, not serious, and spontaneously resolved during treatment.

Fluconazole monotherapy for CM at doses of 800-1000 mg daily has been reported to be effective in a small study of 14 patients treated in Genoa, Italy [Menichetti 1996]. The overall response rate was 67% and only two patients died. Sterilization of CSF occurred at a median of 33 days and time to sterilization was strongly influenced by in vitro sensitivity testing to fluconazole [16 days if minimum inhibitory concentration (MIC) was < 4 mg/L and 56 days for > 4 mg/L]. Serum and CSF levels of fluconazole at steady state were 41 ± 26 and 36 ± 21 mcg/mL, respectively, indicating excellent CSF penetration of drug at these relatively high concentrations. No dose-limiting toxic side effects were reported, but both hematological and hepatic toxicity were noted.

In a recently published clinical study in Uganda, fungicidal activity was greater over two weeks in 30 participants treated with fluconazole at 1200 mg daily for 2 weeks than in 30 participants treated initially with fluconazole at 800 mg daily for 2 weeks [Longley 2008]. After two weeks, participants were followed on 400 mg per day for the next 8 weeks. There were no serious fluconazole dose-related toxicities in either dosing cohort and similar numbers survived in two cohorts.

Summary of Data from Cohort 1

The study’s first cohort (Cohort 1, 1200 mg daily fluconaozole or ampho B-based regimen) was opened to accrual on February 19, 2010. The ACTG A5225 Study Monitoring Committee (SMC) met by conference call on September 28, 2011, to review the A5225 Monitoring Plan and the SMC Report. After carefully reviewing the accrual, safety and efficacy data, the SMC concluded that no significant efficacy or safety concerns were identified. The SMC provided the following recommendations to the team:

1. Because of the low rate of dose-limiting toxicity (DLT) observed with study of 20 participants enrolled in Cohort 1 to date, it is very unlikely that an unacceptable DLT rate would be observed after completion of planned enrollment (5 additional participants) of Cohort 1. The SMC therefore recommends that Cohort 1 should be closed and enrollment of Cohort 2 should begin.

2. An additional ECG should be performed on or near study day 28.

3. When deciding whether or not an event should be considered a DLT and how to manage toxicities, the team should adhere to a strict interpretation of toxicity grades as defined in the DAIDS table. Specifically, both a ≥0.06 sec increase of the QTc above baseline and a QTc ≥0.50 sec should both be considered Grade 3 adverse events since both are criteria for Grade 3 prolonged QTc in the DAIDS adverse event grading table.

4. The SMC noted that azithromycin, which has been reported as a cause of prolonged QTc and torsades de pointes, is not listed among the precautionary medications in the protocol Manual of Operations. Azithromycin should be added to this list and the team should review this list periodically to ensure that it is complete and up to date.

5. The next SMC review should occur when enrollment of Cohort 2 is completed or in one year, whichever comes first.

Cohort 1 was closed to accrual on October 7, 2011, with a total of 29 participants enrolled. Cohort 2 opened to enrollment on October 7, 2011.

Drug-Drug Interactions

Fluconazole has been associated with many clinically important drug interactions. These interactions are mainly due to fluconazole’s inhibitory effects on CYP450 3A4 enzyme and, to a lesser extent, inhibition of 2C8, 2C9, and 2C19. Therefore, medications that are metabolized by CYP450 enzymes may have enhanced concentrations when given concomitantly with fluconazole. Additionally, 11% of fluconazole is metabolized by glucuronidation and oxidation (by P450 enzymes) to inactive metabolites, thereby allowing for effects on fluconazole concentrations by other medications that affect these metabolizing enzymes. Pertinent drug interactions related to this protocol are discussed below.

Nevirapine (NVP) is widely employed in the treatment of HIV in low resource settings and has been used widely in persons who have recovered from CM. Data on the safety of coadministration of fluconazole 200 mg daily with NVP are possibly conflicting as documented in two studies.

The safety and pharmacology of fluconazole 200 mg given with NVP 400 mg once daily was studied in an open-label, single-arm trial examining the PK parameters of fluconazole alone and in combination with NVP in 24 patients on a stable 3-nucleoside ART regimen [Manosuthi, 2007]. The majority of individuals were black (76%) and female (68% in the PK study, 71% in the safety study). Clearance of NVP was halved when fluconazole was added, resulting in an approximate doubling of NVP C_min_, C_max_, and AUC compared with historical data from Boehringer Ingelheim's studies. The high peak NVP levels (C_max_ = 12.9ng/mL; range = 3.0-17.9) seen led to a surprisingly high incidence (25%) of liver toxicity, compared with historical data (2.5%). Nearly all (95%) of the NVP-related AEs occurred after full dosing (400mg daily) of NVP was instituted (day 39 of the study). During this phase, 25% (CI 7-43%) of patients developed serious hepatotoxicity, including 2 cases of clinical hepatitis and 6 cases of transient Grade 4 transaminase elevation. Three cases of rash were seen, 2 (8.3%) macular-papular, and 1 (4.2%) vesicular.

In a retrospective review of liver function by biochemical tests, 69 Thai patients (average weight 59 kg) taking NVP-based ART regimens and 200 mg of fluconazole daily were compared with 392 patients taking 400 mg fluconazole weekly and 225 taking no fluconazole with their ART [Geel, 2004]. No differences in biochemical hepatitis or ability to take the regimens were found.

Based on these data, coadministration of these two drugs is discouraged in A5225/HiFLAC. Furthermore, coadministration of NVP and fluconazole at fluconazole doses > 400 mg daily is prohibited in A5225/HiFLAC. While the decision regarding coadministration of NVP and fluconazole at fluconazole doses of ≤ 400 mg fluconazole daily is left to the discretion of the treating clinician, we strongly urge the institution of weekly monitoring of hepatic enzymes [Manosuthi 2007].

Limited data on the coadministration of fluconazole and EFV have demonstrated a small increase in EFV exposures with no change in fluconazole concentrations. Beginning at week 4, EFV may be used concomitantly with fluconazole without regard to fluconazole dose.

When zidovudine (ZDV) is administered at 200 mg every 8 hours concomitantly with 400 mg fluconazole daily, C_max_ and AUC of ZDV were increased 84.2%, and 74.4%, respectively [Sahai 1994]. Therefore, caution should be used when coadministering these two drugs.

Limited data on the drug interaction between RIF and fluconazole demonstrate enhanced metabolism of concurrently administered fluconazole, thereby reducing the AUC of fluconazole by 23% [Apseloff 1991]. Therefore, the use of RIF is prohibited for A5225/HiFLAC study participants receiving 1200 mg daily fluconazole. RIF may be initiated after these participants have initiated the consolidation dose of 600 mg fluconazole daily (rather than 400 mg daily) or the maintenance dose of 200 mg daily fluconazole. Coadministration of fluconazole with RIF is permitted in the higher induction dose cohorts (1600 mg and 2000 mg daily), however, because the impact of any drug-drug interaction (i.e., the extent to which RIF will reduce fluconazole concentrations) is expected to be lessened at these doses. Medications that are prohibited from coadministration with fluconazole are listed in the A5225/HiFLAC Manual of Operations (MOPS) as are those that may be administered with caution.

Additional information can be obtained from the latest fluconazole package insert.

Considerations Regarding Participant Follow Up

Missing data elements, particularly those required to assess clinical outcomes, potentially confound interpretation of results. In a double-blind multicenter trial, patients with a first episode of AIDS-associated CM were randomly assigned to treatment with higher-dose ampho B (0.7 mg/kg per day) with or without flucytosine (100 mg/kg per day) for 2 weeks, followed by 8 weeks of treatment with itraconazole (400 mg per day) or fluconazole (400 mg per day). Treatment was considered successful if CSF cultures were negative at 2 and 10 weeks or if the patient was clinically stable at 2 weeks and asymptomatic at 10 weeks [van der Horst 1997]. An example of distortions produced by missing data is found in the analysis of this trial. A missing CSF culture was deemed positive and the patient was assigned as failing study therapy (Table 2-3). This assumption may have significantly overestimated the incidence of failure. Thus, losses to follow up and failure to collect critical data have probably inflated incidence of failure in past studies and may obscure clinically meaningful differences in treatment outcomes in comparative studies. Therefore, we will attempt to ensure that sites are adequately prepared to conduct this trial and that necessary training is provided.

Table 2-3: Clinical Failure [van der Horst]

|  | Week 2 | | Week 10 | |
| --- | --- | --- | --- | --- |
| Treatment | AmB+5FC | AmB alone | fluconazole | itraconazole |
| Subjects | N= 202 | N= 179 | N= 151 | N= 155 |
| Success (CSF negative) | 122 (60%) | 91 (51%) | 109 (72%) | 93 (60%) |
| Failure | 80 (40%) | 88 (49%) | 42 (28%) | 62 (40%) |
| CSF culture positive | 47 (59%) | 55 (62%) | 3 (7%) | 8 (13%) |
| No CSF sample | 33 (41%) | 33 (38%) | 39 (93%) | 54 (87%) |

Before implementing A5225/HiFLAC, potential sites will be required to document their ability to initiate the study, enroll adequate numbers of participants, conduct clinical investigations according to good clinical practice, follow the protocol outline as specified, and complete the study procedures [lumbar punctures (LP) and CSF cultures]. During the conduct of the study, sites will be asked to periodically provide the team with documentation of their practices in response to issue-specific inquiries. Submission of a revised A5225/HiFLAC SIP may be required. Collaboration among ACTG international sites in this phase I/II clinical trial should prepare them for the larger phase III trial that will probably follow (see below).

2.1.2 Amphotericin B Deoxycholate (Ampho B)-Based Regimens

Amphotericin B Deoxycholate

Amphotericin B deoxycholate (ampho B) is a macrocyclic, polyene, antifungal produced from a strain of *Streptomyces nodosus*. It is a broad-spectrum antifungal agent that binds to ergosterol in the fungal cell membrane forming large channels through which cellular contents leak, leading to cell death. Toxicity results from the binding of ampho B molecules to cholesterol in the host’s cell membrane. The renal epithelial cells are particularly susceptible to this AE.

The kinetics of ampho B is complicated and not fully elucidated. Ampho B appears to be metabolized in the liver and its metabolites excreted in urine (< 5% of the dose is excreted unchanged in the urine). However, liver disease does not appear to affect its serum level. It is not absorbed by the gastrointestinal tract and is not removed by dialysis.

The toxicities of ampho B include both acute infusion-related and more chronic cumulative toxicities. Acute toxicities include fever, shaking chills, anorexia, nausea, vomiting, chest pain and dyspnea, tachypnea, wheezing, hypotension, and headache. These reactions are common 1 to 3 hours after starting an intravenous infusion and are usually more severe with the first few doses of ampho B.

Cumulative toxicities include renal insufficiency, electrolyte abnormalities, especially hypokalemia and hypomagnesemia, and anemia. Prevention of acute infusion-associated toxicity may be ameliorated with meperidine, non-steroidal anti-inflammatory drugs (NSAIDs), hydrocortisone, and diphenyhydramine. Nephrotoxicity, which may occur in approximately 80% of subjects, is associated with renal tubular damage, renal tubular acidosis, hypokalemia, hypomagnesemia, and possible increased intrarenal vascular resistance. Other reported side effects include anorexia, headache, diarrhea, and suppression of bone marrow, which can lead to anemia.

Table 2-4 below is based on Study 94-0-002, a randomized double-blind study of empiric antifungal therapy for subjects with persistent fever and neutropenia [Walsh, 1999]. There were 592 adults [295 treated with liposomal ampho B (LamB) and 297 treated with ampho B] and 95 juveniles (48 treated with LamB and 47 treated with ampho B) in this study, the largest ever to assess efficacy and toxicity of conventional ampho B. These probably represent the best available toxicity data for ampho B to date.

In 94-0-002, ampho B was reasonably well tolerated. The incidence of common AEs (incidence of 10% or greater) occurring with ampho B, regardless of relationship to study drug, is shown in the following table:

Table 2-4. Study 94-0-002 Common Adverse Events

| Adverse Event by Body System | Ampho B  N=344% |
| --- | --- |
| Abdominal pain Asthenia Back pain Blood product Transfusion reaction Chills Infection Pain Sepsis | 21.8 10.8  7.3  18.6 75.9  9.3 12.8 11.3 |
| Chest pain Hypertension Hypotension Tachycardia | 11.6 16.3 21.5 20.9 |
| Diarrhea Gastrointestinal Hemorrhage Nausea Vomiting | 27.3 11.3 38.7 43.9 |
| Alkaline phosphatase increased ALT (SGPT) increased AST (SGOT) increased Bilirubinemia BUN increased Creatinine increased Edema Hyperglycemia Hypernatremia Hypervolemia Hypocalcemia Hypokalemia Hypomagnesemia Peripheral edema | 19.2 14.0 12.8 19.2 31.1 42.2 14.8 27.9 11.0 15.4 20.9 50.6 25.6 17.2 |
| Anxiety Confusion Headache Insomnia | 11.0 13.4 20.9 14.2 |
| Cough, increased Dyspnea Epistaxis Hypoxia Lung disorder Pleural effusion Rhinitis | 21.8 29.1 20.1 14.8 17.4  9.6 11.0 |
| Pruritus Rash Sweating | 10.2 24.4 10.8 |
| Hematuria | 14.0 |

**Ampho B plus flucytosine**

**The World Health Organization has made the following recommendations with respect to treatment of cryptococcal meningitis in persons with HIV infection [WHO Rapid Advice, December 2011]**

- **For the 2-week induction treatment phase, a regimen containing ampho B combined with flucytosine or fluconazole is the recommended option.**
- **In settings where ampho B is not available, regimens containing fluconazole combined with flucytosine, or high-dose fluconazole monotherapy are alternative options.**
- **For the 8-week consolidation treatment phase, a regimen containing oral fluconazole is the recommended option.**
- **For the maintenance treatment phase, a regimen containing oral fluconazole is the recommended option.**

**The recommendation for use of ampho B combined with fluconazole is based upon four clinical trials in persons with AIDS-associated cryptococcal meningitis [WHO Rapid Advice December 2011, Muzoora 2012, Loyse 2012, Pappas 2009, Brouwer 2004]. In general, the frequency and character of adverse events of combination of ampho B and fluconazole was similar to those who received ampho B alone. A mortality benefit was not demonstrated in either of the two trials in which there were direct comparisons between ampho B and ampho B plus fluconazole [Pappas 2009, Brouwer 2004]. Assessment of mycological efficacy (rate of change in CSF colony counts over time) tended to favor the combination of ampho B and fluconazole.**

| **Author** | **N=** | **Mortality** | | **Early Fungicidal Activity†** | **Comment** |
| --- | --- | --- | --- | --- | --- |
| **Brouwer AE.** | **16** | **AmB Alone**  **2/16 at 2 weeks (12%)**  **3/16 at 10 weeks (19%)** | **AmB+Flu 400 mg**  **5/16 at 2 weeks (31%)**  **7/16 at 10 weeks (43%)** | **AmB = -0.31 log_10_/d**  **AmB+Flu = -0.39 log_10_/d** | **Rate of decline in CSF colony counts AmB+Flu>AmB** |
| **Muzoora CK.** | **30** |  | **5 days AmB+Flu 800 mg**  **7/30 at 2 weeks (23%)**  **8/30 at 10 weeks (28%)** | **AmB+Flu = -0.30 log_10_/d** | **Single arm study with short course AmB (5 days)** |
| **Loyse A.** | **~23** |  | **AmB+Flu 800 mg**  **3/22 at 2 weeks (14%)**  **7/21 at 10 weeks (33%)** | **AmB+Flu 800 mg = -0.38 log_10_/d** | **Random allocation to 4 treatment arms. None assigned AmB alone.** |
|  |  |  | **AmB+Flu 1200 mg**  **4/23 at 2 weeks (17%)**  **6/22 at 10 weeks (27%)** | **AmB+Flu 1200 mg =-0.35 1og_10_/d** |  |
| **Pappas, PA.** | **~48** | **AmB Alone**  **10/47 at 10 weeks (22%)** | **AmB+Flu 400 mg**  **8/48 at 10 weeks (17%)**  **AmB+Flu 800 mg**  **8/45 at 10 weeks (18%)** | **Not Done** | **Random allocation to 3 treatment arms.** |

**† The early fungicidal activity is a measure of the rate of decline in colony counts of *C. neoformans* in the CSF. It is represented as a change in log_10_ colony forming units/day over the course of 14 days**.

In a clinical study performed in Peru, among 47 patients with AIDS and CM treated with amphotericin B 0.7 mg /kg/day without flucytosine for 2 or 3 weeks followed by fluconazole 400 mg daily for 7 or 8 weeks, the mortality rate was 13% in the first 2 weeks and 6% in the next 8 weeks. Early deaths were caused by CM in three out of six patients. In 34% of patients, a modification (dose reduction, temporal suspension or definitive withdrawal) of the ampho B regimen was necessary due to adverse events [Dammert 2008].

5-Fluorocytosine

5-Fluorocytosine (flucytosine or 5-FC) is an oral pyramidine-analogue, antifungal drug that is used in conjunction with ampho B for CM in many regions of the world. Flucytosine penetrates fungal cells, where it is deaminated to fluorouracil by the fungal enzyme cytosine deaminase. Mammalian cells do not convert flucytosine to fluorouracil. Acting as an antimetabolite, fluorouracil competes with uracil, interfering with pyrimidine metabolism and eventually disrupting both RNA and protein synthesis. Flucytosine may also be converted to fluorodeoxyuridylic acid, which inhibits the enzyme thymidylate synthase and disrupts DNA synthesis. Although flucytosine is metabolized to 5-fluorouracil, flucytosine itself does not possess antineoplastic activity.

Resistance develops rapidly if flucytosine is used as a single agent, thus it is always used with another agent. The mechanism of resistance can be loss of the permease necessary for cytosine transport or decreased activity of uridine monophosphate pyrophosphorylase or cytosine deaminase.

Flucytosine toxicity involves the rapidly proliferating tissues such as the bone marrow and the lining of the gastrointestinal (GI) tract. Moderate hypoplasia of the bone marrow occurs, which causes anemia, aplastic anemia, leukopenia, thrombocytopenia, and, rarely, pancytopenia and agranulocytosis. Anemia patients may complain of unusual lethargy or weakness, while leukopenic patients may exhibit pharyngitis and a fever. Thrombocytopenic patients can experience unusual bleeding or bruising. The risk of developing bone marrow toxicity from flucytosine is increased with prolonged, high serum flucytosine concentrations (>100 ucg/ml), renal dysfunction, or concurrent ampho B therapy. Eosinophilia has also been reported.

Manifestations of flucytosine toxicity on the rapidly proliferating lining of the GI tract include: abdominal pain, anorexia, diarrhea, and nausea/vomiting. Nausea/vomiting also may be a result of a CNS mechanism since high concentrations of flucytosine are achieved in CSF. Other GI AEs reported with flucytosine include duodenal ulcer (GI peptic ulcer), hemorrhage (GI bleeding), ulcerative colitis, and xerostomia. Genitourinary AEs to flucytosine have included azotemia, creatinine and BUN elevation, crystalluria, and renal failure (unspecified).

Oral absorption of flucytosine ranges from 75-90%. Food decreases the rate of absorption, while the extent of absorption remains unchanged. Peak serum concentrations of 30-45 mcg/ml are reached within 6 hours after a 2 g oral dose in patients with normal renal function. After prolonged dosing, peaks are reached 1-2 hours post-dose. Peaks are higher, more prolonged, and reached more slowly in patients with renal impairment. Steady-state serum flucytosine concentrations should range from 50-100 mcg/ml to prevent development of resistant strains and toxicity.

Because flucytosine is a small molecule with limited protein binding, it is widely distributed throughout the body. Concentrations in liver, kidneys, spleen, heart, and lungs are equal to serum concentration, while CSF concentrations are 60-90% of that in the serum. The volume of distribution is 0.68 L/kg in patients with normal renal function, and is decreased to as little as 0.4 L/kg in patients with renal impairment. Protein binding is 2-4%.

Flucytosine is not metabolized. While fungal cells convert flucytosine to fluorouracil intracellularly, only trace amounts of fluorouracil can be detected in the serum. Elimination is primarily renal. Over 90% is excreted by glomerular filtration as unchanged drug. Elimination half-life is a function of creatinine clearance; in patients with normal renal function, it varies from 2.5-6 hours, and can be as long as 11-60 hours if creatinine clearance is less than 2 ml/minute. Drug interactions with 5FC are unusual.

Ampho B and flucytosine may be synergistic against *Cryptococcus neoformans*. This combination may allow the total daily dose of ampho B to be lowered. While lower total doses of ampho B can decrease the risk of nephrotoxicity from this agent, ampho B-induced renal dysfunction can increase flucytosine's serum concentrations and, possibly, its myelosuppressive properties. Flucytosine is eliminated essentially unchanged in the urine. It is important to adjust the dose of flucytosine if renal impairment occurs.

In adults, dosing is 50-150 mg/kg/day orally (PO) in divided doses every 6 hours. For severe infections, the usual dosage is 100-150 mg/kg/day PO in divided doses every 6 hours. When used in combination with conventional amphotericin B in HIV-infected patients, some clinicians suggest an initial dosage of 75-100 mg/kg/day PO in divided doses every 6 hours. Dosage can then be adjusted based on flucytosine serum concentration and/or renal function.

In a completed clinical study in South Africa, among 64 patients treated with ampho B at either 0.7 or 1.0 mg/kg daily combined with flucytosine at 100 mg/kg daily for 14 days followed by fluconazole at 400 mg daily for 8 weeks, 16 (25%) died by 10 weeks. Serious ampho B and flucytosine drug-related toxicity was uncommon (3 subjects, 5%). About half of the deaths were attributable to CM and half to competing morbidities [Bicanic, Wood, et al. 2008].

Additional information may be obtained from the latest amphotericin B deoxycholate and flucytosine package inserts.

### 2.2 Rationale

Study Design

The ultimate objective of this study is to determine whether an oral regimen (e.g., fluconazole alone) is acceptably potent and well tolerated for the treatment of HIV-associated CM. A phase III clinical trial to compare the selected dose of fluconazole with a standard ampho B-based regimen would be of interest to providers where ampho B is available and should follow this study. In settings where use of an ampho B-based regimen is not feasible, fluconazole is often used at suboptimal doses. Establishing whether doses of fluconazole of 1200 mg or greater are tolerable and more effective than lower doses would be immediately applicable to patient care in these settings.

For application to areas where ampho B-based regimens are feasible, a direct comparison of ampho B-based regimens with the optimal doses of fluconazole in a larger phase III study is needed. In preparation for such a study, the goal for A5225/HiFLAC is to determine the MTD of fluconazole and to provide pilot data on its efficacy. Thus, we propose that HIV-infected participants with CM be assigned to one of up to 3 induction doses of fluconazole alone: 1200, 1600, or 2000 mg/day. These regimens will be tested sequentially so that safety will be assessed at each level before the decision is made to expose new participants to the next higher dose.

The goal in Stage 1 is to determine fluconazole’s MTD: the highest dose that can be safely used [defined as a dose that results in a ≤25% rate of fluconazole dose-limiting toxicities (DLTs)] and provide preliminary information on efficacy. Determination of MTD will identify one or more high doses of fluconazole that are safe to use as induction regimens for CM treatment. During Stage 2, the identified fluconazole dose(s) will be further tested and compared with amphotericin B for efficacy. This comparison aims to identify the lowest dose with the highest efficacy. At the end of this phase I/II clinical trial, we will be well prepared to develop the phase III study mentioned above and to obtain additional AE reports in a larger study population.

A5225/HiFLAC will use the induction-consolidation approach now recommended for ampho B-based regimens. When a participant’s CM is controlled, as indicated by a negative CSF culture, fluconazole doses will be reduced to 400 mg daily to complete a 10-week course of therapy. This regimen is justified because prior experience has shown that 97% of participants whose CSF culture became negative after 2 weeks of treatment and who were then given 400 mg fluconazole daily remained negative when sampled again at 10 weeks [van der Horst, 1997]. After 10 weeks, fluconazole at 200 mg daily will be initiated and should be continued through study end. The 200 mg dose should continue until sustained immune reconstitution, defined as a CD4 count of > 200 cells/mm^3^ or a CD4/CD8 cell ratio > 0.2 for 6 months, has occurred. Therefore, maintenance treatment after study end is expected and recommended although it will not be provided by the study. Prior to registering to the protocol, sites will be required to describe how they plan to provide appropriate post-study treatment and follow-up for study participants.

Comparisons of Data with Historic Data

Since A5225/HiFLAC is the first CM trial conducted by the ACTG at sites outside the US, the participant and disease characteristics may differ from those observed in prior ACTG studies, making a comparison of this study data to the historic data problematic. This issue can be partially addressed by looking at the known disease risk factors (baseline CSF cryptococcal antigen, CD4 T-lymphocyte counts, serum albumin) associated with outcome and assessing the comparability of the populations [Robinson 1999]. Unmeasured (or at least unreported) characteristics such as disease severity (HIV and cryptococcal disease), concurrent infections (e.g., pulmonary and extrapulmonary tuberculosis (TB), hepatitis B, and/or hepatitis C) are more likely to be prevalent in an international cohort and could reduce the clinical efficacy in this study. Such biases could lead to an underestimation of the efficacy of fluconazole relative to the historical control population. Moreover, it is unlikely that the culture sterility rate at 2 weeks would be as good with fluconazole compared to an ampho B-based regimen, and so high-dose fluconazole will be used for at least 4 weeks in all participants.

Justification for Ampho B-Based Induction Cohorts

A5225/HiFLAC will be conducted predominantly in sites outside the U.S., where the predominant standard of care (SoC) treatment for CM is an ampho-B based regimen, with ampho B administered IV for 14 days at between 0.7 and 1.0 mg/kg/day, followed by fluconazole. Such treatment is associated with high rates of AEs and has not been well studied in all settings. Therefore, ampho B-based regimen cohorts will be included in this study to provide pilot data for the design of the planned phase III comparison trial described above.

Sites participating in this study will be those that have access to an ampho B-based induction treatment for their CM patients.

At the discretion of the site investigator and based on local standard of care (SoC) CM treatment, participants who experience a TLT to ampho B within the first 2 weeks and prior to receipt of a total of 8.4 mg/kg ampho B may receive alternative treatment such as liposomal amphotericin B, where available, or may enter Step 2 and be provided with fluconazole at daily dose of 800 mg or the maximum locally approved dose.

Measures of Microbiological Outcome

Quantitative CSF cultures have not been used in previous ACTG-sponsored trials, but have been used to assess treatment success with ampho B and in the assessment of microbial responses to combination antifungal therapy [Pitisuttithum 2001; Brouwer 2004]]. The quantitative culturing technique is simple and reliable [99% confidence interval (CI) to 0.5 log in animal studies] [Larsen 2004]. The results of these quantitative cultures will provide important supplementary information about the potential effectiveness of the selected dose of fluconazole.

Dose Adjustments for Toxicity

Because many AEs of fluconazole are probably dose dependent, we will allow dose reductions by site investigators to manage side effects during the induction and consolidation steps (see section 7.0). Dose reduction may be preferable to discontinuation of fluconazole for three reasons: 1) treatment of CM is mandatory, 2) lower fluconazole doses may be adequate for many participants, and 3) alternative regimens may not be available or may be contraindicated. Such reduction may be temporary while the cause of the toxicity is ascertained, or may be continued through the step in which it is initiated.

Dose adjustment guidelines for ampho B side effects are described in section 5 of the A5225/HiFLAC MOPS. Step 2 has been included in the protocol for participants who discontinue ampho B because of ampho B intolerance. Each site will be allowed to determine the appropriate Step 2 dose of fluconazole, either 800 mg daily or the maximum locally approved dose.

ART Initiation and Potential Drug-Drug Interactions

Evidence-based guidelines are not available for optimal timing of ART initiation in the setting of acute CM. Site clinicians may initiate ART when they feel it is appropriate to do so. However, because fluconazole substantially increases NVP levels and may increase hepatotoxicity [Geel 2004], NVP-based ART may be used only when participants are taking fluconazole at a dose of ≤ 400 mg. Because NVP-based ART is commonly employed in resource-limited settings, site clinicians are advised to seek alternative antiretroviral drugs (ARVs) for participants on higher doses of fluconazole.

There is the potential for two-way interactions between fluconazole and non-nucleoside reverse transcriptase inhibitors (NNRTIs) and drugs belonging to the class of protease inhibitors (PIs). As mentioned previously, fluconazole is an inhibitor of CYP450 3A4 and, to a lesser extent, 2C8, 2C9, and 2C19 [Niwa 2005]. Therefore, levels of medications that are substrates of CYP450 enzymes may be raised in the presence of fluconazole. These include the NNRTIs NVP and EFV, and the PIs nelfinavir, amprenavir, fosamprenavir, lopinavir, and atazanavir.

A small study presented at the 16th Conference on Retroviruses and Opportunistic Infections (CROI 2009) suggests that starting NVP-based regimens during fluconazole-based treatment of CM may be dangerous [Makadzange 2010]. Early (< 72 hrs) versus late (> 10 wks) initiation of ART with stavudine, lamivudine and NVP) were compared in patients treated for CM in a study in Harare, Zimbabwe. Overall mortality rates were high (62%) and greater in the early (23/26 = 82%) than in the late (8/28 = 37%) ART-treated patients. Death occurred much earlier in the early-treated patients (median 5 vs. 35 weeks, HR = 2.4, p<.03). This striking elevation in mortality in those started immediately on NVP-based ART appears to contradict conclusions of another similar study in RLSs (ACTG A5164) in which a subgroup of CM patients, like the majority of patients in the study who had Pneumocystis pneumonia, survived for longer when ART was started early. The mechanisms for these early deaths are unclear and while they could be due to immune reconstitution inflammatory syndrome (IRIS) reactions to CM or other coexisting OIs, a more plausible explanation is a NVP–fluconazole interactions leading to either greater NVP toxicity from higher levels or most likely, decreased fluconazole efficacy from lower levels.

Because 11% of fluconazole is metabolized by glucuronidation and oxidation (by P450 enzymes), the concomitant use of medications that affect these enzymes may modestly affect fluconazole concentrations [Debruyene 1993]. The results of specific drug interaction studies published to date follow. None of these studies concluded that the PI dose should be adjusted when co-administered with fluconazole. Saquinavir (SQV) concentrations are increased 50-56% with the co-administration of fluconazole 200 mg daily [Koks 2001]. The coadministration of fluconazole 200 mg daily and ritonavir (RTV) 200 mg every 6 hours, resulted in an average 12-14% increase in RTV concentrations and a less than 20% increase in fluconazole concentrations [Cato 1997]. Finally, the co-administration of indinavir (IDV) 1000 mg every 8 hours and fluconazole 400 mg once daily resulted in a 19-24% decrease in IDV AUC, with no effects on other PK parameters and no significant changes in fluconazole concentrations [De Wit 1998]. Data on the co-administration of SQV or IDV with RTV are not available; therefore, the effect of these combinations is not known. However, due to the 20% increase in fluconazole when given with RTV, increases in fluconazole concentrations are likely to result when PIs are combined with RTV [Cato 1997].

Additional considerations of potential induction or inhibition include the following: amprenavir and fosamprenavir may decrease fluconazole concentrations through induction of fluconazole metabolism [Lexiva prescribing information November 2005], while RTV given concomitantly or not with nelfinavir, SQV, IDV, or atazanavir, and lopinavir/RTV have the potential to increase fluconazole concentrations through inhibition of fluconazole metabolism [Ernest 2005].

Pharmacology Studies

PK evaluations are included in this study to compare concentrations of fluconazole in plasma and CSF. We anticipate that the PK (in both plasma and CSF) of fluconazole will increase linearly with dose as has been observed at lower doses. However, the PK may be altered by saturation of absorption and/or drug clearance. Based on the data from the CCTG study, we anticipate that higher induction doses may sterilize cryptococci in the CSF sooner or more frequently, related to higher levels of drug in the CSF. Direct measurement of plasma and CSF fluconazole levels will help us assess this hypothesis.

Additionally, there is potential for fluconazole to increase drug concentrations of RIF. Likewise, RIF is likely to decrease fluconazole concentrations. Ideally, the concomitant use of these drugs would be avoided. However, in resource-limited settings, other options are not always available. The impact of this drug-drug interaction at high fluconazole doses (1600 and 2000 mg daily) is expected to be lessened but the extent of overall exposures is not known and will therefore be evaluated in each of these cohorts.

Furthermore, restricted data on the coadministration of fluconazole and EFV have demonstrated a small increase in EFV plasma concentrations which is not assumed to be clinically significant and no dose adjustment currently is recommended. There are no data related to interactions between EFV and fluconazole at doses of >400mg/day. Trough samples will be collected during the induction step from all participants in high-dose fluconazole cohorts; evaluation of these samples may provide information on the effect of co-administration of fluconazole and EFV where EFV is initiated during this step.

Assessment of the Lateral Flow Cryptococcal Antigen (LFCrAg) Assay

*C. neoformans* produces a unique polysaccharide-lipid antigen [Goodman 1971, Doering 2009]. Detection of this agent by latex agglutination is a rapid diagnostic test which can facilitate early diagnosis. A novel lateral flow cryptococcal antigen (LFCrAg) assay is now commercially available for use in serum. This new assay, LFCrAg, has also proven useful in plasma, CSF, and urine, but is not currently approved for such use in the USA [Jarvis, Harrison et al. 2011, Jarvis, Percival et al. 2011, Lindsley 2011].

The advantages of the LFCrAg assay are:

- It is a very simple assay.
- It is rapid, taking only 10-15 minutes to define the presence of cryptococcal antigen in the sample being tested.
- It is considerably less expensive than the latex agglutination assay and appears to have equivalent sensitivity and specificity.
- Reagents can be shipped and stored at room temperature.
- The test appears to have expanded utility to include testing of plasma and urine, in addition to CSF and serum.
- The test may provide an equivalent general measure of severity of illness when a semi-quantitative measure of antigen content (ie, titration) is performed.

Correlations of the LFCrAg assay and semi-quantitative measures of antigen content will be evaluated using baseline samples of serum, plasma, urine, and CSF and compared to results obtained by the latex agglutination assay. Associations of the baseline quantitative CSF culture and the CSF semi-quantitative measures of antigen content will also be evaluated. Additional associations of follow-up quantitative CSF cultures and CSF semi-quantitative measures of antigen content will be assessed by both latex agglutination and the LFACrAg assay.

A qualitative measure for the presence of urinary antigen will also be performed using baseline urine samples and, if applicable, urine samples collected at time of disease progression.

Exclusion of HIV-Negative Participants

Patients who are HIV-negative respond differently to CM treatment and have a lower rate of relapse than do HIV-positive patients. While we anticipate that few CM-positive participants will be HIV-negative, we will exclude the rare HIV-negative participant to ensure that administration of high-dose fluconazole alone is assessed in HIV-positive participants only in this phase I/II study.

Pregnancy and Use of Hormonal Contraceptives

Because fetal abnormalities have been reported with fluconazole, prevention of pregnancy in sexually active participants is imperative.There are several published case reports of a rare, distinct pattern of birth defects in infants whose mothers were treated with high dose fluconazole (400-800 mg/day) during most or all of the first trimester.

Data on expected effects at higher doses of fluconazole are not available. It is possible that hormonal contraceptives coadministered with high dose fluconazole could result in drug concentrations similar to those associated with increased risks of thrombosis and thromboembolism. Because CM is associated with cerebrovascular infarction, use of oral hormonal contraceptives in this study is prohibited while a participant is taking a daily dose of fluconazole >200 mg.

## 3.0 STUDY DESIGN

Overview

A5225/HiFLAC is a randomized, open-label phase I/II dose escalation and validation study of the safety, tolerability, and therapeutic effect of an induction-consolidation strategy of high-dose fluconazole alone for the treatment of CM in HIV-infected participants. Comparison of this study’s results with historical data may be problematic and estimates of the performance of this drug for treatment of CM in resource-limited settings are very limited. For these reasons, a cohort treated with an ampho B-based regimen will be included in this study.

Stages, Steps, and Cohorts

Stages

The study will proceed in two stages. In Stage 1, Dose Escalation, up to three induction doses of fluconazole will be tested in sequentially enrolled cohorts. In Stage 2, Dose Validation, induction doses of fluconazole that were found to be safe in Stage 1 will be tested in simultaneously enrolled cohorts **along side 1 cohort that will receive high dose fluconazole plus 5FC**. In both Stage 1 and Stage 2, randomization to a cohort in which an ampho B-based regimen is administered in the induction phase will be included.

Steps

Within each stage, there will be multiple treatment steps for each cohort. For participants randomized to receive fluconazole only **or fluconazole plus 5FC**, the steps will be induction (Step 1), consolidation (Step 3), and maintenance (Step 4). For participants randomized to receive an ampho B-based induction regimen, the possible treatment steps will be induction (Step 1), induction following ampho B intolerance (Step 2), consolidation (Step 3), and maintenance (Step 4). Treatment within each step is described in section 5.1.

Cohorts

At study entry, participants will be randomized to one of at least 2 and at most 4 cohorts. The number of cohorts that are enrolling at any one time will depend on which stage of the study is open. Descriptions of the possible cohorts are presented in section 5.1

Stage 1 (Dose Escalation) Cohorts

Within Stage 1, a single fluconazole cohort and a single ampho B-based cohort will be open to enrollment at any one time. Participants will be randomized at Step 1 entry to receive a high dose of fluconazole for 4-10 weeks, or to receive the SoC dose of an ampho B-based regimen for approximately 2 weeks followed by fluconazole. The composition of the ampho B-based regimen will be determined by local SoC, but will include 0.7-1.0 mg/kg daily ampho B. The length of ampho B treatment will be determined in part by participants’ tolerance to the regimen. The total amount of ampho B that is administered to a participant in Step 1 is expected to be at least 8.4 mg/kg.

Opening of fluconazole cohorts at increasingly higher induction doses of fluconazole (ie, escalation of the fluconazole induction dose) will occur only after all available safety data, including, at a minimum, all data through day 14 for the last participant enrolled in the current cohort, have been reported and reviewed. Doses will not be escalated within any cohort. Dose escalation of fluconazole will stop at 2000 mg daily or earlier if the MTD is identified.

When either the MTD of fluconazole is reached or the week 10 visit for the final participant who entered the 2000 mg/day cohort is completed, all data through week 10 on all participants will be reviewed before Stage 2 of the study will be opened (see below).

Stage 2 (Dose Validation)

In Stage 2 (Dose Validation), participants will be randomized to receive an induction treatment of either an ampho B-based regimen or one of several doses of fluconazole at or below the MTD identified in Stage 1 **or to a dose of fluconazole that is at or below the MTD plus 5FC**. Before Stage 2 is opened, the protocol will be amended so that the specific dose or doses of fluconazole that will be tested in Stage 2 can be identified.

Stage 1 and Stage 2 Evaluations

Participants will be monitored for safety and toxicity throughout the study period.

The primary efficacy outcome measure is the change in quantitative CSF culture for cryptococcus. During screening or at entry, CSF samples for culture will be collected from all participants. At week 2, CSF samples will again be collected from all participants and will be cultured. Additional CSF samples will be collected and cultured at weeks 4, 6, 8, and 10, until a negative culture following 2 weeks of incubation is obtained. Treatment failure for any participant is defined as a week 10 CSF culture (which is read by or at week 12) that is positive for *C. neoformans*.

If a week 10 CSF culture is positive (by or at week 12), participants may discontinue study treatment and be treated according to local standard practice, at the discretion of the site investigator. Participants who discontinue study treatment prematurely should be encouraged to remain on study, off study treatment and should be encouraged to return for the week 24 visit.

PK samples will be obtained at specified time points from all participants enrolled in fluconazole induction cohorts (see section 10.0 for sampling information). Plasma, urine, serum, and CSF samples will be collected from all participants and stored for retrospective evaluation of novel diagnostic methodologies (collection schedule is presented in section 6.1).

## 4.0 SELECTION AND ENROLLMENT OF PARTICIPANTS

NOTE: Inclusion and exclusion criteria listed below apply to both Stage 1 and Stage 2 of the study.

### 4.1 Inclusion Criteria – Step 1

- - 1. Cryptococcal meningitis documented either by a positive CSF cryptococcal culture, a positive CSF India ink preparation, or a positive CSF cryptococcal antigen latex agglutination test within 7 days prior to entry.

**NOTE: Patients may be enrolled into the study with a cryptococcal meningitis (CM) infection that is documented solely by a test result from a referring laboratory (ie, not the site’s DAIDS-approved laboratory). However, CM must be confirmed by Day 5 at a laboratory that is participating in EQA (eg, the site laboratory that has received approval from the ACTG to conduct A5225). Treatment must not be delayed while awaiting confirmation of the screening test result.**

- - 1. CSF collection for quantitative cryptococcal culture within 72 hours prior to study entry or planned to be performed at study entry.

4.1.3 HIV-1 infection, documented by any licensed rapid HIV test or HIV enzyme or chemiluminescence immunoassay (E/CIA) test kit at any time prior to study entry and confirmed by or within 10 days after study entry by a licensed Western blot or a second antibody test by a method other than the initial rapid HIV and/or E/CIA, or by HIV-1 antigen, plasma HIV-1 RNA viral load.

NOTE: The term ‘licensed’ refers to an FDA-approved kit which is required for all IND studies. For non-US sites, ‘licensed’ refers to a kit that has been certified or licensed by an oversight body within that country and that has been validated internally. However, non-US sites are encouraged to use FDA-approved methods for IND studies.

The World Health Organization and the US Centers for Disease Control and Prevention guidelines mandate that confirmation of the initial test result must use a test that is different from the one used for the initial assessment. A reactive initial rapid test should be confirmed by either another type of rapid assay or an E/CIA that is based on a different antigen preparation and/or different test principle (eg, indirect versus competitive), or a Western blot or a plasma HIV-1 RNA viral load.

- - 1. Men and women age ≥ 16 years.

4.1.5 Ability to take oral medications.

NOTE: Administration of fluconazole tablets via nasogastric tube is permitted.

4.1.6 For patients with a co-morbid complication of HIV, including opportunistic infections, **ability to monitor treatment efficacy (CSF sampling) and toxicity management of study drugs**, as judged by the site investigator.

NOTE: **CSF sampling may not be possible or safe in the setting of CNS mass lesions. Such participants should be entered and continued in study only in consultation with neurosurgery/neuroradiology after assessment of the safety of CSF sampling.**

4.1.7 For female participants of reproductive potential (defined as girls who have reached menarche or women who have not been post-menopausal for at least 24 consecutive months, ie, who have had menses within the preceding 24 months, or have not undergone surgical sterilization [eg, hysterectomy, or bilateral oophorectomy or salpingotomy]) a negative serum or urine pregnancy test result must be obtained within 2 days prior to study entry.

4.1.8 All participants must agree not to participate in the conception process (eg, active attempt to become pregnant or to impregnate, sperm donation, in vitro fertilization).

4.1.9 If participating in sexual activity that could lead to pregnancy, female study participants must agree to the simultaneous use of two forms of contraceptive (from the list below) during such sexual activity, and male study participants must agree to use a condom during such sexual activity. This requirement continues while the study participant is on study treatment and for 6 weeks after fluconazole has been discontinued.

- Condoms (male or female) with or without a spermicidal agent
- Diaphragm or cervical cap with spermicide
- IUD (intrauterine device)
- Injectable DepoProvera
- Tubal ligation

NOTE: Use of oral hormone-based contraceptives is prohibited while a study participant is taking any study-provided fluconazole.

4.1.10 Study participants who are not of reproductive potential (defined as women who have been post-menopausal for at least 24 consecutive months, or women who have undergone surgical sterilization [eg, hysterectomy, or bilateral oophorectomy or salpingectomy], or men who have documented azoospermia), are eligible without the requirement to use contraceptives.

NOTE: Lack of reproductive potential requires written documentation or oral communication from a clinician or clinician’s staff documented in source documents.

4.1.11 Willingness and ability to adhere to dose schedules and mandatory procedures.

4.1.12 Measured or calculated creatinine clearance of ≥50 mL/min within 3 days prior to study entry.

A calculator is available at the Data Management Center at [*https://www.fstrf.org/ACTG/*](https://www.fstrf.org/ACTG/) or at [*http://www.fstrf.org/ACTG/index.html*](http://www.fstrf.org/ACTG/index.html).

4.1.13 The following laboratory values within 3 days prior to study entry:

- aspartate aminotransferase (AST), alanine aminotransferase (ALT), and alkaline phosphatase ≤ 5 X the upper limit of normal (ULN)
- total bilirubin ≤ 2.5 X ULN
- absolute neutrophil count (ANC) ≥ 750/mm^3^
- platelet count ≥ 50,000/mm^3^
- hemoglobin ≥ 7.0 g/dL

4.1.14 Ability and willingness of the participant or legal guardian/representative to give informed consent.

4.1.15 Availability at the site of at least 2 weeks of its standard of care ampho B-based regimen.

### 4.2 Exclusion Criteria - Step 1

4.2.1 Expected survival of ≤ 2 weeks, in the opinion of the site investigator and, if available, the primary care provider.

4.2.2 For patients with a co-morbid complication of HIV, anticipated difficulty, in the opinion of the site investigator, in judging response to study treatment as a result of the co-morbid complication or the drugs used to treat it.

4.2.3 Breastfeeding.

4.2.4 A prior episode of CM, either as indicated by patient or as noted in patient medical records.

4.2.5 Use of any of the following drugs, within the time periods described:

- warfarin within 3 days prior to study entry
- terfenadine or cisapride within 24 hours prior to study entry
- other drugs known to interact with fluconazole, the co-administration of which may lead to life-threatening side effects, within 10 days prior to study entry (see section 3 of the current A5225/HiFLAC MOPS)
- drugs that may decrease the effect of fluconazole, or whose co-administration may increase their own toxicity or decrease their own efficacy, within 24 hours prior to study entry (see section 3 of the current A5225/HiFLAC MOPS)
- phenytoin, carbamazepine, cyclosporin A, tacrolimus, sirolimus, astemizole, or long-acting barbiturates within 7 days prior to study entry
- more than 72 cumulative hours of systemic treatment **thought to be effective in the management of cryptococcal meningitis** with **fluconazole at >400 mg/day or itraconazole > 200 mg/day or amphotericin B >0.3 mg/kg/day or liposomal amphotericin B >0.3 mg/kg.day or flucytosine >25 mg/kg/day** for any reason within 14 days prior to study entry.

4.2.**6** Known allergy, sensitivity to, or intolerance of fluconazole or other imidazole or triazole compounds, or to ampho B or other components of the standard of care ampho-B based regimen.

4.2.**7** History of clinically significant cardiac disease, in opinion of site investigator, including symptoms of ischemia, coronary artery disease, congestive heart failure, or arrhythmia.

4.2.**8** ECG (electrocardiogram) with QTc interval greater than 450 msec within 7 days prior to study entry.

4.2.**9** History of CNS disorder (excluding mood disorders) or concurrent CNS disorder(s) that, in the opinion of the investigator, would interfere with assessment of efficacy (e.g., ability to perform CSF sampling) such as lymphoma, neurocysticercosis, or toxoplasmosis.

4.2.**10** Receipt of investigational drug therapy within 30 days prior to study entry without prior approval of the A5225/HiFLAC core team.

4.2.1**1** Active drug or alcohol use, dependence, or other conditions that in the opinion of the site investigator would jeopardize the safety of a participant in the study or would render the person unable to comply with the study plan.

### 4.3 Inclusion Criteria – Step 2

4.3.1 Randomization to an ampho B-based regimen in Step 1.

4.3.2 Receipt of at least one dose of ampho B-based regimen in Step 1.

4.3.3 Premature discontinuation of ampho B in response to the occurrence of any treatment-limiting toxicity, as described in section 5 of the A5225/HiFLAC MOPS.

### 4.4 Exclusion Criteria – Step 2

4.4.1 Receipt of fluconazole monotherapy in Step 1.

4.4.2 Receipt of ≥8.4 mg/kg ampho B.

4.4.3 At or beyond Day 17 in Step 1.

### 4.5 Inclusion Criteria - Step 3

4.5.1 For participants in Step 1 who are currently receiving study-provided fluconazole, with no plans to discontinue study treatment, a negative CSF culture after 2 weeks incubation from a sample obtained at or before week 6 (days 35-49).

4.5.2 For participants in Step 1 who are currently receiving an ampho B-based regimen or alternative treatment, completion of approximately 2 weeks of treatment.

NOTE: The total amount of ampho B received by a participant is expected to be approximately 8.4 mg/kg.

4.5.3 For participants in Step 2 who are currently receiving study-provided fluconazole, with no plans to discontinue study treatment, negative CSF culture after 2 weeks incubation from a sample obtained at or before week 6 (day 35-49).

### 4.6 Exclusion Criteria – Step 3

4.6.1 On study treatment beyond week 10 (day 77) in Step 1 or Step 2.

4.6.2 Currently off study treatment.

### 4.7 Inclusion Criterion – Step 4

4.7.1 On study treatment at week 10 (day 63-77), with no plans to discontinue study treatment.

### 4.8 Exclusion Criterion – Step 4

4.8.1 Currently off study treatment.

### 4.9 Study Registration and Enrollment Procedures

- - 1. All sites intending to register to conduct this study must first complete and submit the SIP found under the Current Version tab on the A5225/HiFLAC PSWP. Each SIP will be reviewed by the protocol team. Sites should wait to be advised of the outcome of this review before submitting their registration materials.
    2. Prior to implementation of this protocol and subsequent amendments, each site must have the protocol and consent form approved by its local institutional review board (IRB) or ethics committee (EC). Protocol documents must be registered with and approved by the DAIDS Regulatory Support Center (RSC) Protocol Registration Office. Protocol registration must occur before the site can enroll any participants into the study.
    3. Details of the research study will be carefully discussed with potential study participants. The participant (or parent or legal guardian) will be asked to read and sign the approved consent form. If the participant and legal guardian are illiterate, the process for consenting illiterate participants, as defined by the local IRB/EC, should be followed.
    4. For participants from whom informed consent has been obtained, an ACTG Screening Checklist must be entered through the Data Management Center (DMC) Subject Enrollment System.
    5. Participants who meet eligibility criteria for A5225/HiFLAC will be randomized to an open cohort according to standard ACTG DMC procedures.
    6. An ACTG Screening Failure Results form must be completed and keyed into the database for participants from whom informed consent is obtained but who do not enroll into A5225/HiFLAC.
    7. Participants who meet eligibility criteria for Steps 2, 3, and 4 must be registered through the DMC and receive a new SID number.

### 4.10 Coenrollment Guidelines

Sites in the US are strongly encouraged to coenroll participants in A5128 (“Plan for Obtaining Informed Consent to Use Stored Human Biological Materials (HBM) for Currently Unspecified Analyses”). Sites outside the US are strongly encouraged to coenroll participants into A5243 (“Plan for Obtaining Saliva Samples at Non-U.S. Clinical Research Sites for Currently Unspecified Genetic Analyses”). Coenrollment in A5128 or A5243 does not require permission from the protocol chairs. Approval for coenrollment into any other study must be obtained from the chairs of A5225/HiFLAC and of the study in question.

## 5.0 STUDY TREATMENT

### 5.1 Regimens, Administration, and Duration

A5225/HiFLAC study treatment is defined as study-provided oral fluconazole or as an amphotericin B deoxycholate-based regimen that could include 5-fluorocytosine **or fluconazole**, according to local standard of care.

Amphotericin B deoxycholate will be administered intravenously; 5-fluorocytosine, when administered, will be administered orally. Neither amphotericin B deoxycholate nor 5-fluorocytosine **or fluconazole for use with amphotericin B deoxycholate** will be provided by the study.

Stage 1**, which has been completed, was** a dose escalation study of daily fluconazole from 1200 mg up to 2000 mg. Dose escalation may end before the 2000 mg dose if the MTD has been identified. There will be up to 4 dosing steps for each participant. The study will include a cohort in which participants will be treated with an amphotericin B deoxycholate-based induction regimen.

Stage 2 is a dose validation study of fluconazole doses tested in Stage 1. When either the MTD of fluconazole is reached or the week 10 visit for the final participant randomized to the 2000 mg/day cohort is completed, all data on all participants, including week 10 data for the final randomized participant, will be reviewed. Before Stage 2 is opened, the protocol will be amended; the specific dose or doses of fluconazole at or below the MTD that is or are to be tested in Stage 2 will be identified.

In Stage 2, participants will be randomized to receive an induction treatment of either an amphotericin B deoxycholate-based regimen or one of up to three doses of fluconazole.

At entry to Step 1 in Stage 1, participants will be randomized to receive a single oral high-dose induction regimen of fluconazole or an amphotericin B deoxycholate-based induction regimen. At entry to Step 1 in Stage 2, participants will be randomized to receive either one of up to 3 high doses of fluconazole or to receive an amphotericin B deoxycholate-based regimen. The fluconazole doses listed below are for those participants who enter the study weighing ≥ 60kg. Doses for participants weighing < 60 kg at entry must be modified in the induction step only. The modified regimens for these participants are presented in section 7.4.2, under Fluconazole Dose Modifications.

5.1.1 Regimens

5.1.1.1 Stage 1 Regimens

Step 1 (Induction)

Within each fluconazole cohort in Stage 1, a different single high dose of fluconazole will be administered in the induction step (Step 1). The decision to open each subsequent cohort (ie, cohorts 2 and 3) will be made after a review of all safety data through week 2 for all participants in the current cohort. Throughout Stage 1, amphotericin B deoxycholate will be administered in Step 1 at a dose of 0.7-1.0 mg/kg daily as part of the local standard of care amphotericin B deoxycholate-based regimen for CM treatment. Where available, alternative treatment to ampho B, such as liposomal amphotericin B, may be used in Step 1 through week 2 for participants who become intolerant of ampho B.

Step 2 (Induction Following Amphotericin B Deoxycholate Intolerance)

Participants in amphotericin B deoxycholate-based induction cohorts who experience a TLT may enroll in the induction following amphotericin B deoxycholate intolerance step (Step 2) and will receive 400-800 mg daily fluconazole. Each site will be expected to identify the daily fluconazole dose that they will administer to their participants.

Step 3 (Consolidation)

Participants who are taking fluconazole in Step 1 or Step 2 may enroll in the consolidation step (Step 3) if they have a negative CSF culture documented by week 8. It is possible that some participants will enter Step 4 without enrolling in Step 3.

Participants who received approximately 2 weeks of ampho B-based regimen in Step 1 may enroll in the consolidation step (Step 3) at week 2, as described in section 4.5. A negative CSF culture result is not required for these participants.

Participants in Step 3 will receive fluconazole at a dose of 400 mg daily until week 10. Participants in Step 3 who are taking rifampin, however, will receive fluconazole at a dose of 600 mg daily.

Step 4 (Maintenance)

At week 10, all participants will either enroll in the maintenance step (Step 4) and receive fluconazole at a dose of 200 mg daily through study end or will discontinue study treatment.

Table 5-1: Stage 1 Regimens

| Cohort | N | Induction Step (Step1)  Daily Dose | Induction Following Amphotericin B Deoxycholate Intolerance Step (Step 2)  Daily Dose | Consolidation Step (Step 3)  Daily Fluconazole Dose | Maintenance Step (Step 4) Daily  Fluconazole Dose |
| --- | --- | --- | --- | --- | --- |
| 1A^1^ | 24 | Fluconazole 1200 mg | N/A | 400 mg  Or 600 mg with rifampin | 200 mg |
| 1B^1^ | 8 | Amphotericin B deoxycholate-based regimen; 0.7-1.0 mg/kg amphotericin B deoxycholate | Fluconazole 400-800 mg | 400 mg  Or 600 mg with rifampin | 200 mg |
| 2A | 24 | Fluconazole 1600 mg | N/A | 400 mg  Or 600 mg with rifampin | 200 mg |
| 2B | 8 | Amphotericin B deoxycholate-based regimen; 0.7-1.0 mg/kg amphotericin B deoxycholate | Fluconazole 800 mg or maximum locally approved dose | 400 mg  Or 600 mg with rifampin | 200 mg |
| 3A | 24 | Fluconazole 2000 mg | N/A | 400 mg  Or 600 mg with rifampin | 200 mg |
| 3B | 8 | Amphotericin B deoxycholate-based regimen; 0.7-1.0 mg/kg amphotericin B deoxycholate | Fluconazole 800 mg or maximum locally approved dose | 400 mg  Or 600 mg with rifampin | 200 mg |

^1^ Cohort 1 was closed to accrual on October 7, 2011.

Following review of all data through week 10 in Stage 1, a determination will be made regarding which induction doses of fluconazole will be tested in Stage 2.

- - - 1. Stage 2 Regimens

In Stage 2, participants will enter Step 1 and be randomized to one of up to three high-dose fluconazole induction cohorts or to an amphotericin B deoxycholate-based regimen cohort. The protocol will be amended prior to Stage 2 opening so that the specific doses of fluconazole that will be tested in Stage 2 can be identified. Unlike in Stage 1, multiple fluconazole cohorts may be open to enrollment simultaneously during Stage 2. Enrollment to Steps 2, 3, and 4 will proceed as described for Stage 1.

Table 5-2: Potential Stage 2 Regimens

| Cohort | N | Induction Step (Step1)  Daily Dose | Induction Following Amphotericin B Deoxycholate Intolerance Step (Step 2)  Daily Dose | Consolidation Step (Step 3)  Daily Fluconazole Dose | Maintenance Step (Step 4) Daily  Fluconazole Dose |
| --- | --- | --- | --- | --- | --- |
| 4 | 24^1^ | Amphotericin B deoxycholate-based regimen; 0.7-1.0 mg/kg amphotericin B deoxycholate | Fluconazole 800 mg or maximum locally approved dose | 400 mg  Or 600 mg with rifampin | 200 mg |
| 5 | 24 | Fluconazole TBD | N/A | 400 mg  Or 600 mg with rifampin | 200 mg |
| 6 | 24 | Fluconazole TBD | N/A | 400 mg  Or 600 mg with rifampin | 200 mg |
| 7 | 24 | Fluconazole TBD | N/A | 400 mg  Or 600 mg with rifampin | 200 mg |

^1^ Between Stages 1 and 2, a total of 48 participants will receive an amphotericin B deoxycholate-based regimen; if fewer than 24 participants receive an amphotericin B deoxycholate-based regimen in Stage 1, the number receiving it in Stage 2 will be increased.

- - - 1. Management of Mycological Failure at Week 10

At week 10, participants with mycological failure (ie, whose most recent CSF culture is positive) may enroll in Step 4 or may, at the site investigator’s discretion, discontinue study treatment and instead be treated according to best standard local practice, administered by the site investigator. This may include, but is not limited to, switching to an alternative regimen with continued CSF monitoring every two weeks.

5.1.2 Fluconazole Dosage Adjustments

Dose modification of fluconazole is only permitted for renal impairment, intolerability, toxicity, or during the induction step for weight. Weight-based modifications must be implemented at the first dose. Specific guidelines are provided in section 7.4.

5.1.3 Administration

Fluconazole

If needed to improve tolerance in the induction step, fluconazole may be administered with or without food and may be given in 2-4 approximately equally divided doses during a day.

Amphotericin B Deoxycholate

Amphotericin B deoxycholate should be administered intravenously under close clinical observation by medically trained personnel. Sites should refer to the current package insert. For additional information on administration and clinical management, sites may consult the Amphotericin B Deoxycholate Administration section in the A5225/HiFLAC MOPS.

5.1.4 Duration

Fluconazole: up to 24 weeks per participant.

Amphotericin B deoxycholate: Approximately 2 weeks unless discontinuation is mandated due to toxicities. Treatment may be extended beyond Day 14 at the discretion of the site investigator. Reasons for extending amphotericin B deoxycholate administration include, but are not limited to, persisting positive CSF cultures, unchanged or worsened overall clinical status compared to baseline, and/or new neurologic deficits or physical findings suggesting progressive disease.

Sites are responsible for ensuring that appropriate CM treatment will be available as needed after study end or following premature treatment or study discontinuation.

### 5.2 Product Formulation and Preparation

Fluconazole: 200 mg tablets. Tablets should be stored below 86ºF (below 30ºC).

### 5.3 Pharmacy: Product Supply, Distribution, and Accountability

Fluconazole will be provided by Pfizer.

Neither amphotericin B deoxycholate nor 5-fluorocytosine **or fluconazole for use with amphotericin B deoxycholate** will be provided by the study.

5.3.1 Study Product Acquisition/Distribution

**Study-provided f**luconazole will be available through the National Institute of Allergy and Infectious Diseases (NIAID) Clinical Research Products Management Center (CRPMC). The site pharmacist can obtain the study product for this protocol by following the instructions in the manual, *Pharmacy Guidelines and Instructions for DAIDS Clinical Trials Networks,* in the section Study Product Management Responsibilities.

5.3.2 Study Product Accountability

The site pharmacist is required to maintain complete records of all study products received from the NIAID CRPMC and subsequently dispensed. All unused study product must be returned to the NIAID CRPMC after the study is completed or terminated. The procedures to be followed are provided in the manual, *Pharmacy Guidelines and Instructions for DAIDS Clinical Trials Networks,* in the section Study Product Management Responsibilities. The non-US pharmacists must follow the instructions in *Pharmacy Guidelines and Instructions for DAIDS Clinical Trials Networks* for the destruction of study provided fluconazole. The site will follow local procedures and regulations for the disposal or destruction of the other locally provided study products.

The pharmacist or investigator is responsible for maintaining dispensing records for all components of any amphotericin B deoxycholate-based regimen that is administered in the induction phase.

### 5.4 Concomitant Medications

Information regarding recommended, prohibited, and precautionary concomitant medications is found on the A5225 PSWP. In order to avoid adverse events caused by drug interactions, whenever a concomitant medication or study treatment is initiated or a dose changed, investigators must review the concomitant medication's and study treatment’s most recent package inserts, investigator's brochure, or updated information from the DAIDS to obtain the most current information on drug interactions, contraindications, and precautions.

## 6.0 CLINICAL AND LABORATORY EVALUATIONS

### 6.1 Schedule of Events

6.1.1 Screening and Study Entry SoE: All Participants

| Evaluation | Screening | Study Entry/ Study Treatment Initiation (Week 0) |
| --- | --- | --- |
|
| Documentation of HIV Status | X | Must be confirmed by Day 10 |
| Documentation of CM Status | X | **Must be confirmed by Day 5** |
| Medical/Medication History | X | X |
| Length of Hospitalization |  | X |
| Concomitant Medications |  | X |
| ART (Initiation and Modifications) |  | Record if initiated |
| Study Treatment Initiation |  | X |
| Complete Physical Examination |  | X |
| Clinical Assessments | X |  |
| Height and Weight (see section 6.3.10) |  | X |
| Glasgow Coma Scale |  | X |
| ECG | X |  |
| Hematology and Chemistries (see section 6.3.13) | X | X |
| Stored Serum and Plasma |  | X |
| Stored Urine |  | X |
| Pregnancy Test | X |  |
| CD4+ Cell Count (for timing, see section 6.2.2) | X | |
| Plasma HIV-1 RNA (for timing, see section 6.2.2) | X | |
| Lumbar Puncture/Opening Pressure (for timing, see section 6.2.2) | X | |
| CSF Cryptococcal Antigen (for timing, see section 6.2.2) | X | |
| CSF Analyses: Glucose, Protein, & Cell Counts (for timing, see section 6.2.2) | X | |
| Stored CSF Sample (for timing, see section 6.2.2) | X | |
| Mandatory CSF Cultures (for timing, see section 6.2.2) | X | |
| CSF Cryptococcal Isolate Storage (for timing, see section 6.2.2) | X | |

6.1.2 SoE for Participants Randomized to Fluconazole in Step 1

| Evaluation | Step 1/Week 1 | | | Step1 | | Step 1 and/or 3 | | | Step 4 | | Other Possible Visits | |
| --- | --- | --- | --- | --- | --- | --- | --- | --- | --- | --- | --- | --- |
|  | Day (Window) | | | Week (Window) | | | | | | |  |  |
|  | D. 1 | D. 4  (3-5) | D. 7  (6-8) | 2  (D.  12-17) | 4  (D.  25-31) | 6  (D.  39-45) | 8  (D.  53-59) | 10  (D.  67-73) | | 24  (D.  161-175) | Progression of Symptoms | Premature Treatment or Study  D/C |
| Documentation of HIV Status | Must be confirmed by Day.10 | | | |  | | | | | | | |
| **Documentation of CM** | **Must be confirmed by Day 5** | | | |  | | | | | | | |
| Length of Hospitalization | Document time of discharge and any readmission for treatment of CM | | | | | | | | | | | |
| Concomitant Medications | X | X | X | X | X | X | X | | X | X | X | X |
| ART (Initiation and Modifications) | At each visit, record any ART initiation and any subsequent modifications. | | | | | | | | | | | |
| Study Treatment Modifications | X | X | X | X | X | X | X | | X | X | X | X |
| Complete Physical Examination |  |  |  | X |  |  |  | | X | X | X | X |
| Clinical Assessments | X | X | X |  | X | X | X | |  |  |  |  |
| Height and Weight (for timing, see section 6.3.10) |  |  |  | Record at each study-required PK collection visit | | | | |  |  |  | X |
| Glasgow Coma Scale | X | X | X | X | X | X | X | | X | X | X | X |
| ECG |  | X | X |  | X |  |  | |  |  |  |  |
| Hematology and Chemistries |  | X | X | X | X | X | X | | X |  | X | X |
| Pregnancy Test | Repeat as indicated | | | | | | | | | |  |  |
| CD4+ Cell Count | At time of ART initiation. | | | | | | | | X |  | X |  |
| Plasma HIV-1 RNA | At time of ART initiation. | | | | | | | | X |  | X |  |

6.1.2 SoE for Participants Randomized to Fluconazole in Step 1 (Cont’d)

| Evaluation | Step 1/Week 1 | | | Step1 | | Step 1 and/or 3 | | Step 4 | | Other Possible Visits | |
| --- | --- | --- | --- | --- | --- | --- | --- | --- | --- | --- | --- |
|  | Day (Window) | | | Week (Window) | | | | | |  |  |
|  | D. 1 | D. 4  (3-5) | D. 7  (6-8) | 2  (D.  12-17) | 4  (D.  25-31) | 6  (D.  39-45) | 8  (D.  53-59) | 10  (D.  67-73) | 24  (D.  161-175) | Progression of Symptoms | Premature Treatment or Study  D/C |
| Lumbar Puncture/Opening Pressure | Document whenever performed; record opening pressure. | | | X | Document whenever performed; record opening pressure. | | | | | X | If LP done, record opening pressure. |
| CSF Glucose, Protein, & Cell Counts |  |  |  | X | Perform on each study-required CSF sample | | | |  | X |  |
| Stored CSF Sample |  |  |  | X | Perform on each study-required CSF sample | | | |  | X |  |
| Mandatory CSF Cultures |  |  |  | X |  |  |  |  |  | X |  |
| CNS Imaging, if performed (see section 6.3.21) |  |  |  |  |  |  |  |  |  | X |  |
| Possible CSF Cultures |  |  |  |  | Perform until previous culture is found to be negative. | | | |  |  |  |
| CSF Cryptococcal Isolate Storage (see section 6.3.23) |  |  |  | Perform at each positive CSF culture. | | | | |  | X |  |
| PK Sampling for Participants Taking Fluconazole (for timing, see section 6.3.24) |  |  |  | X | Perform as directed while participant is taking high dose fluconazole | | | |  | X | See Section 10.2. |
| Stored Plasma |  |  |  |  | Collect at each study-required PK collection visit. | | | |  | X |  |
| Stored Serum |  |  |  |  | X |  | | |  | X |  |
| Stored Urine |  |  |  |  |  | | | |  | X |  |
| Adherence Assessment | X | X | X | X | x | x | x | x | X | X | X |

6.1.3 SoE for Participants Randomized to Ampho B in Step 1

| Evaluation | Step 1/Week 1 and/or Step 2 | | | Step 1 and/or 2 | | Step 2 and/or 3 | | | Step 4 | | Other Possible Visits | |
| --- | --- | --- | --- | --- | --- | --- | --- | --- | --- | --- | --- | --- |
|  | Day (Window) | | | Week (Window) | | | | | | |  |  |
|  | D. 1 | D. 4  (3-5) | D. 7  (6-8) | 2  (D.  12-17) | 4  (D.  25-31) | 6  (D.  39-45) | 8  (D.  53-59) | 10  (D.  67-73) | | 24  (D.  161-175) | Progression of Symptoms | Premature Treatment or Study  D/C |
| Documentation of HIV Status | Must be confirmed by Day 10 | | | |  | | | | | | | |
| **Documentation of CM** | **Must be confirmed by Day 5** | | | |  | | | | | | | |
| Length of Hospitalization | Document time of discharge and any readmission for treatment of CM | | | | | | | | | | | |
| Concomitant Medications | X | X | X | X | X | X | X | | X | X | X | X |
| ART (Initiation and Modifications) | At each visit, record any ART initiation and any subsequent modifications. | | | | | | | | | | | |
| Study Treatment Modifications | X | X | X | X | X | X | X | | X | X | X | X |
| Complete Physical Examination |  |  |  | X |  |  |  | | X | X | X | X |
| Clinical Assessments | X | X | X |  | X | X | X | |  |  |  |  |
| Height and Weight (see section 6.3.10) | X | X | Record prior to initiation of fluconazole. | | | | | | | | | X |
| Glasgow Coma Scale | X | X | X | X | X | X | X | | X | X | X | X |
| ECG |  | X | X |  | X |  |  | |  |  |  |  |
| Hematology & Chemistries | Twice weekly while on ampho B; at each study visit thereafter. | | | | | | | | | | | |
| Pregnancy Test | Repeat as indicated | | | | | | | | | |  |  |
| CD4+ Cell Count | At time of ART initiation. | | | | | | | | X |  | X |  |
| Plasma HIV-1 RNA | At time of ART initiation. | | | | | | | | X |  | X |  |

6.1.3 SoE for Participants Randomized to Ampho B in Step 1 (Cont’d)

| Evaluation | Step 1/Week 1 and/or Step 2 | | | Step 1 and/or 2 | | Step 2 and/or 3 | | Step 4 | | Other Possible Visits | |
| --- | --- | --- | --- | --- | --- | --- | --- | --- | --- | --- | --- |
|  | Day (Window) | | | Week (Window) | | | | | |  |  |
|  | D. 1 | D. 4  (3-5) | D. 7  (6-8) | 2  (D.  12-17) | 4  (D.  25-31) | 6  (D.  39-45) | 8  (D.  53-59) | 10  (D.  67-73) | 24  (D.  161-175) | Progression of Symptoms | Premature Treatment or Study  D/C |
| Lumbar Puncture/Opening Pressure | Document whenever performed; record opening pressure. | | | X | Document whenever performed; record opening pressure. | | | | | X | If LP done, record opening pressure. |
| CSF Glucose, Protein, & Cell Counts |  |  |  | X | Perform on each study-required CSF sample | | | |  | X |  |
| Stored CSF Sample |  |  |  | X | Perform on each study-required CSF sample | | | |  | X |  |
| Mandatory CSF Cultures |  |  |  | X |  |  |  |  |  | X |  |
| CNS Imaging, if performed (see section 6.3.21) |  |  |  |  |  |  |  |  |  | X |  |
| Possible CSF Cultures |  |  |  |  | Perform until previous culture is found to be negative. | | | |  |  |  |
| CSF Cryptococcal Isolate Storage (see section 6.3.23) |  |  |  | Perform at each positive CSF culture. | | | | |  | X |  |
| Stored Serum |  |  |  |  | X |  | | |  | X |  |
| Stored Plasma |  |  |  |  | | | | |  | X |  |
| Stored Urine |  |  |  |  | | | | |  | X |  |
| Adherence Assessment (to be collected once participant is taking fluconazole) |  | X | X | X | X | X | X | X | X | X | X |

### 6.2 Timing of Evaluations

6.2.1 Pre-Enrollment Evaluations

Screening

Screening evaluations to determine eligibility must be completed within 3 days prior to study entry unless otherwise specified. Entry may take place on the same day as screening if all required information is available and acceptable. In the event of a delay in entry into study beyond 24 hours, **800**mg FCZ daily for a maximum of 3 days or total dose of **2400**mg is permissible.

The ACTG Screening Failure Results Form must be completed for any patient who is screened for this study but who does not enroll into it.

6.2.2 On-Study Evaluations

Entry

On-study evaluations are performed only after a participant is enrolled into the study. However, the following evaluations do not need to be repeated at entry if performed within 72 hours prior to entry: CD4 cell count, plasma HIV-1 RNA, LP with opening pressure recorded, CSF cryptococcal antigen test, CSF cultures initiated (quantitative and qualitative), CSF analyses, and stored CSF sample. Storage of a cryptococcal isolate from CSF collected at screening or entry is sufficient.

Participants must begin study-assigned treatment within 72 hours after entry, however, initiation of study-assigned treatment within 24 hours after entry is strongly encouraged.

Post- Entry

The visit schedule through day 7 should be based on the first day of treatment (day 1 = first day after first dose of fluconazole or ampho B-based regimen), not solely on the date of enrollment. The visits listed for Days 4 through 7 should be completed ± 1 day of those time points as indicated in the SoE, section 6.1.2 and 6.1.3.

The week 2 visit should occur on day 14, but may be completed 12-17 days after initiation of study treatment.

The weeks 4-10 visits should occur on the week noted in the SoE, but may be completed ± 3 days as indicated in the SoE, sections 6.1.2 and 6.1.3. The week 24 visit may be completed ±7 days of that week, as indicated in sections 6.1.2 and 6.1.3. Note that for participants whose previous CSF culture was positive, at least 14 days must elapse between visits.

Step 1 (Induction)

Participants are expected to remain on Step 1 from 2 to 10 weeks depending on the treatment to which they are randomized and their response to that treatment. Participants randomized to ampho B will register to Step 3 after approximately 2 weeks of ampho B treatment. Some participants taking ampho B may register to Step 2 (see below) before entering Step 3.

Participants randomized to fluconazole will remain in Step 1 until week 10 or until a CSF culture is negative, whichever is earlier.

Step 2 (Induction Following Amphotericin B Deoxycholate Intolerance)

Participants in ampho B-based induction cohorts who experience a treatment-limiting toxicity may either remain in Step 1 and receive alternative treatment, if available, or may enroll in the induction following ampho B intolerance step (Step 2) and will receive 800 mg daily fluconazole or the maximum locally approved dose. Each site will be expected to identify the daily fluconazole dose that it will administer to its participants.

Step 3 (Consolidation)

Participants who are taking **study-provided** fluconazole in Step 1 or Step 2 may enroll in the consolidation step (Step 3) if they have a negative CSF culture documented by week 8. It is possible that some participants will enter Step 4 without enrolling in Step 3. Participants randomized to ampho B in Step 1 who do not enter Step 2, are expected to enter Step 3 after approximately 2 weeks, regardless of culture outcomes.

Participants in Step 3 will receive fluconazole at a dose of 400 mg daily until week 10. Participants in Step 3 who are taking rifampin, however, will receive fluconazole at a dose of 600 mg daily.

Step 4 (Maintenance)

At week 10, all participants currently on study treatment are expected to enroll in the maintenance step (Step 4) and receive fluconazole at a dose of 200 mg daily through study end.

6.2.3 Progression of Symptoms

Participants who experience a recurrence or significant worsening of CNS signs or symptoms (see section 6.3.9) should be evaluated according to sections 6.1.2 and 6.1.3 of the SoE of this protocol and managed according to section 10 of the A5225/HiFLAC MOPS. The evaluations listed in the sections noted above should be conducted if the recurring or progressing symptoms are severe and persistent enough to be outside the variations in clinical response to successful CM therapy.

6.2.4 Premature Treatment or Study Discontinuation

Confirmatory HIV Test/Qualifying CSF culture

If **any of the following is true in the given time frame, then** the participant will be discontinued from study treatment, followed for up to 2 weeks, and then discontinued from the study**:**

- the confirmatory HIV test is negative
- the qualifying CSF culture is negative after 2 weeks of incubation
- **a confirmatory CSF antigen titer is negative by Day 5 AND no positive result from the qualifying CSF culture is obtained through Day 4**
- a study entry quantitative culture was required but not performed

In such cases, the participant will be treated as deemed appropriate by his/her primary physician. These participants should complete the evaluations in the Premature Treatment or Study Discontinuation column in the SoE at the time of treatment discontinuation and again approximately 2 weeks later, for study discontinuation; no fluconazole trough PK sample should be collected at these participants’ study discontinuation visit.

Premature Treatment Discontinuation

All participants who prematurely discontinue study treatment (see section 8.1) at any time during the study should complete the Premature Treatment or Study Discontinuation evaluations (per the SoE) within one week after treatment discontinuation. With the exception of participants randomized to ampho B who prematurely discontinue ampho B in Step 1 and who are eligible to register to Step 2, participants who prematurely discontinue study treatment will remain on the step they are on at the time of treatment discontinuation (without registering to subsequent steps) and will continue to be followed on study, off treatment per the SoE, except as noted in sections 6.3.22 and 6.3.24.

Premature Study Discontinuation

Participants who discontinue the study prematurely (see section 8.2) should complete the Premature Treatment or Study Discontinuation evaluations (per the SOE) at the time of study discontinuation.

6.2.5 Evaluations for Study Participants Who Do Not Start Study Treatment

Participants who do not start study treatment within 72 hours after entry must be discontinued from the study. No additional follow up is required. All week 0 forms must be completed and keyed.

- - 1. Study Completion Evaluations

The week 24 evaluations will be completed as the participant’s final on-study visit.

### 6.3 Special Instructions and Definitions of Evaluations

All clinical and laboratory information required by this protocol is to be present in the source documents. Sites must refer to the Source Document Guidelines on the DAIDS Web site for information about what must be included in the source document: <http://www.niaid.nih.gov/labsandresources/resources/daidsclinrsrch/documents/sourcedocappndx.pdf>.

All stated evaluations are to be recorded on the case report forms (CRFs) and keyed into the database unless otherwise specified.

6.3.1 Documentation of HIV Status

HIV-1 infection will be documented as described in section 4.1.3.

If the confirmatory test of the HIV status reported at screening is negative, the participant must discontinue study treatment and be followed as described in section 6.2.4, above, to monitor any toxicity.

- - 1. Documentation of CM Status

CM must be documented either by a positive CSF cryptococcal culture **or** a **locally licensed** positive CSF India ink preparation, or a positive CSF cryptococcal antigen latex agglutination test within 7 days prior to entry. **A positive CSF India ink or cryptococcal antigen latex agglutination test received from a referring laboratory must be confirmed within 5 days after study entry by CSF antigen testing from an approved laboratory or with a positive entry CSF culture by Day 5.**

**If the confirmatory test of CM is negative, the participant must be discontinued from study treatment immediately, referred immediately to his/her health care provider for appropriate management, and be followed as described in section 6.2.4, above, to monitor for any toxicity.**

6.3.3 Medical/Medication History

Medical History

The medical history must include all AIDS-defining diagnoses using WHO criteria (see the A5225/HiFLAC MOPS or <http://www.who.int/hiv/pub/prev_care/en/arvrevision2003en.pdf>) for the participant’s lifetime and all diagnoses identified by the ACTG criteria for clinical events and other diagnoses ([http://www.fstrf.org](http://www.fstrf.org/)) for the 3 months prior to study entry. For current ACTG criteria, refer to the appendix identified in the study CRF. Any allergies to any medications and their formulations must be recorded on the CRF. Record diagnoses on the CRF and key within 48 hours.

Medication History

A medication history, limited to ARVs, opportunistic infection (OI) treatment and prophylaxis, and TB and antifungal therapy taken in a participant’s lifetime, must be present in CRFs, and must include actual or estimated start and stop dates. In addition, all medications taken within 14 days prior to study entry should be recorded on the CRF.

Current alternative therapies and traditional treatments are to be recorded as yes/no on the CRF. If answered yes, the alternative therapy or traditional treatment will be listed in the source documents only.

6.3.4 Length of Hospitalization

The date of hospital/clinic admission for the current episode of CM must be recorded at entry. Ongoing hospitalization and the date of discharge, if it falls at or before week 24, must also be recorded. In addition, subsequent re-admission to hospital for treatment of CM must be recorded, along with date of discharge, if prior to week 24.

6.3.5 Concomitant Medications

Study staff are expected to work closely with hospital staff during any participant’s hospitalization to ensure that prohibited concomitant medications are avoided if possible, and to ensure on a daily basis that all medications provided to a participant have been documented.

The Concomitant Medications section of the A5225 MOPS includes links to websites that can be consulted for information about known or possible drug-drug interactions.

Any OI treatment and/or prophylaxis, and TB and antifungal therapy other than ampho B or study-provided fluconazole that was started or stopped since the last visit must be recorded on the CRFs. For 5-fluorocytosine **or fluconazole that is administered with ampho B**, the dose must be recorded on the CRFs also.

Current alternative therapies and traditional treatments are to be recorded as yes/no on the CRF. If answered yes, the alternative therapy or traditional treatment will be listed in the source documents only.

6.3.6 ART (Initiation and Modification)

All modifications to ART must be recorded on the CRF. Modifications include initiation as well as any changes in dose or formulation, interruptions of 2 or more consecutive days, or discontinuations for any reason.

Although initiation of ART prior to week 4 is discouraged for participants taking high dose fluconazole, participants who initiate ART that does not contain NVP prior to Step 3 may remain on study. Participants who initiate NVP while taking high dose fluconazole (ie, at a dose higher than 400 mg daily) must be discontinued from study treatment.

6.3.7 Study Treatment Modifications

All modifications to ampho B-based regimens or to study-provided fluconazole, including initial doses, participant-initiated and/or protocol-mandated interruptions of 2 or more consecutive days, any modifications, and any permanent discontinuation of treatment will be recorded on the CRFs at each visit and keyed within 48 hours. Participant-initiated and protocol-mandated interruptions include both inadvertent and deliberate interruptions of the ampho B-based regimens or of study-provided fluconazole.

6.3.8 Complete Physical Examination

A complete physical examination is required as noted in the SoE.

In addition to all evaluations listed under Clinical Assessments below, a complete physical exam will include a neurological exam as well as axillary or oral temperature (Centigrade or Fahrenheit), pulse, blood pressure, respiratory rate, and examination of the following: head, eyes, ears, nose, and throat (HEENT), neck, chest, heart, abdomen, extremities, and skin. The neurological examination will include cranial nerve assessments with emphasis on cranial nerves II, III, IV, V, VI, and VIII, evaluation of the presence of meningeal irritation, a fundoscopic examination, a functional assessment using the A5225/HiFLAC study-specific neurology CRF, and an overall Karnofsky performance status assessment.

At entry, current functional assessment will be determined. Prior functional assessment will be reported for a time within approximately 6 weeks prior to the onset of the current illness. Information may be provided by a friend or family member if the participant cannot recall or otherwise provide the requested information.

6.3.9 Clinical Assessments

At the visits indicated in the SoE, clinical assessments should be conducted and should be driven by any previously identified or new signs or symptoms or diagnoses that the subject has experienced since the previous visit.

Vital Signs

Vital signs (temperature, pulse, respiratory rate, and blood pressure) must be recorded on the CRFs.

Signs and Symptoms

At entry, all signs and symptoms occurring within 3 days prior to entry must be recorded and keyed within 48 hours. In Stage 1, Steps 1 and 2, beginning on Day 1, all Grade ≥ 3 signs, symptoms, and toxicities, and those that lead to a change in treatment, regardless of grade, must be recorded and keyed within 48 hours. For Stage 1, Steps 3 and 4, and in all of Stage 2, regardless of step, all Grade ≥ 3 signs, symptoms, and toxicities, and those that lead to a change in treatment, regardless of grade, must be recorded.

In addition, in Stages 1 and 2, the identification of symptoms that lead to the evaluations described under section 6.2.3 must be brought to the attention of the study core team via e-mail within 2 weeks after the site’s becoming aware of these symptoms.

Sites must refer to the DAIDS Table for Grading the Severity of Adult and Pediatric Adverse Events, Version 1.0, dated December 2004, which can be found on the DAIDS RSC Web site: [http://rsc.tech-res.com/safetyandpharmacovigilance/.](http://rsc.tech-res.com/safetyandpharmacovigilance/.  ) .

Diagnoses

All diagnoses identified by the ACTG criteria for clinical events and other diseases must be recorded and keyed within 48 hours.

Classification of Progression of Symptoms

In addition, using data generated from evaluations conducted as described under 6.2.3, investigators will classify progression of symptoms as one of the following:

1) microbiological failure (i.e., positive CSF culture)

2) complication of CM (e.g., obstructive hydrocephalus or vascular complications such as venous or arterial thrombosis)

3) IRIS to CM causing increased inflammation after exposure to ART

4) a new CNS OI (e.g., toxoplasmosis, PML, CNS lymphoma)

5) possibly related to CM but mechanism indeterminate

6) other defined complication unrelated to CM.

6.3.10 Height and Weight

Height and weight must be measured and recorded at entry. If weight cannot be measured, an estimated weight, based on recent records or according to site investigator judgment, may be used (a notation that the weight has been estimated must be included in these cases). The participant’s actual weight must be measured directly as soon as possible; if actual weight is obtained within 4 days after entry, dose adjustments may be made based on this weight, as warranted.

The same height measurement will be used for all PK visits for participants who were at least 21 years old at study entry; for all others, height must be measured and recorded at each PK visit.

After entry, weight must be measured and recorded as indicated on the SoE.

6.3.11 Glasgow Coma Scale

Neurological assessments using the Glasgow Coma Scale (which can be found in section 7 of the A5225/HiFLAC MOPS) must be performed at every study visit beginning at study entry and must be recorded.

6.3.12 ECG

Resting ECG results, including heart rate and measured QT interval, must be recorded as indicated in the SoE, sections 6.1. The QT interval should ideally be taken from limb lead II. See section 7.2 for clinical management directions for participants with prolonged QT interval on study. Note that when recording QT interval at visits after entry, the site is responsible for documenting any change relative to the baseline value.

6.3.13 Hematology and Chemistries

At entry, all screening and entry laboratory values must be recorded and keyed within 48 hours. In Stage 1, Step 1, beginning on Day 1, and in Stage 1, Step 2, all Grade ≥ 2 laboratory toxicities, any laboratory toxicities that lead to a change in treatment, regardless of grade, and all estimated creatinine clearance values must be recorded and keyed within 48 hours. In Stage 1, Steps 3 and 4, and in all of Stage 2, regardless of step, all Grade ≥ 2 laboratory toxicities, any laboratory toxicity that leads to a change in treatment, and all estimated creatinine clearance values must be recorded.

Sites must refer to the DAIDS Table for Grading the Severity of Adult and Pediatric Adverse Events, Version 1.0, dated December 2004, which can be found on the DAIDS RSC Web site: [http://rsc.tech-res.com/safetyandpharmacovigilance/.](http://rsc.tech-res.com/safetyandpharmacovigilance/.  )

Hematology

Hemoglobin, hematocrit, white blood cell count (WBC), differential WBC, platelets and absolute neutrophil count (ANC).

Because of the frequent occurrence of ampho B toxicities, the tests listed above should be performed twice weekly while ampho B is administered.

Blood Chemistries

Glucose, electrolytes (sodium, potassium, chloride, bicarbonate), total bilirubin, AST (SGOT), ALT (SGPT), albumin, alkaline phosphatase, and creatinine.

Because of the frequent occurrence of toxic side effects, serum potassium, bicarbonate, creatinine, and serum magnesium level should be performed twice weekly while ampho B is administered.

Estimated Creatinine Clearance

Creatinine clearance should be estimated each time that a creatinine level is determined (unless creatinine clearance is measured directly).

To estimate creatinine clearance, use the following method of Cockcroft and Gault:

For men: {(140 - age in years) x (body weight in kg)} ÷ (serum creatinine in
mg/dL x 72).

For women: use the same calculation as for men, then multiply the result by 0.85.

A calculator is available at the Data Management Center Web site at <https://www.fstrf.org/ACTG/>.

6.3.14 Pregnancy Test

For women of reproductive potential: Serum or urine β-HCG (the upper limit for sensitivity of a urine test is 50 mIU/mL; tests that are more sensitive, i.e., with lower detectable limits, are acceptable) must be performed as indicated on the SoE. Beginning at entry, results of any pregnancy tests must be recorded on the CRF.

Women who become pregnant on study must discontinue study treatment immediately. These women may remain on study, off study treatment, after signing a pregnancy consent form, or may elect to go off study. Regardless of their decision, these women should seek CM treatment outside the study. See sections 6.2.4 and 7.6 for additional instructions. Pregnancy outcome data will be collected and recorded in the woman’s source documents and on the CRF even though this may occur after study end.

6.3.15 CD4+ Cell Counts

CD4+ cell counts (both absolute and subset percentage counts) should be performed at the same laboratory, if possible, throughout the study. Within the U.S., laboratories must be CLIA certified. Outside the U.S., laboratories must be ACTG approved. Because of the diurnal variation in CD4+ cell counts, samples for each participant should be obtained consistently in either the morning or the afternoon throughout the study.

CD4+ counts must be obtained from all participants at the visits indicated in the SoE. Note that if CD4 counts are obtained within 72 hours prior to study entry, a repeat of this determination is not required at study entry.

6.3.16 Plasma HIV-1 RNA

The HIV-1 RNA quantitations must be performed real time using any approved HIV RNA quantitation assay. Within the U.S., laboratories must be CLIA certified and have DAIDS approval. Outside the U.S., laboratories must be participating in the VQA and be DAIDS approved. HIV-1 RNA data will be reported on the CRF when they are not reported through the Laboratory Data Management System (LDMS).

HIV-1 RNA values must be obtained from all participants at the time points indicated in the SoE. Note that if a sample for HIV-1 RNA is obtained within 72 hours prior to study entry, a repeat of this determination is not required at study entry.

6.3.17 Lumbar Puncture/Opening Pressure

Throughout the study, all lumbar punctures (LPs), including those performed as part of clinical care (eg, for diagnosis, monitoring, or for the relief of intracranial pressure) should be recorded on the CRF, along with the opening pressure. Section 9 of the A5225/HiFLAC MOPS contains instructions for the preferred method for performing LPs on study.

6.3.18 CSF Cryptococcal Antigen

Detection of CSF cryptococcal antigen will be performed at screening or entry using a latex-Cryptococcus antigen detection system **and should be positive within after study entry or the participant is to be discontinued (see section 6.2.4). A negative CSF antigen titer by latex agglutination will not exclude the participant if the entry CSF culture is positive for *C. neoformans*.** A 2-fold titer will be reported.

6.3.19 CSF Glucose, Protein, & Cell Counts

Each time an LP is performed for collection of CSF, glucose and protein determinations and cell counts (indicating numbers of red blood cells and numbers of white blood cells only) must be performed real time on the CSF.

6.3.20 Stored CSF Sample

Each time an LP is performed for collection of CSF, a CSF sample must be stored.

6.3.21 Mandatory CSF Cultures

**To remain in the study, participants’ qualifying CSF culture must be positive by Day 4 if a confirmatory CSF cryptococcal antigen titer by latex agglutination is not positive by Day 5.**

During screening or at study entry and again at week 2, a CSF sample for culture must be obtained from all participants. Each time a CSF sample for culture is required, both a qualitative and a quantitative culture must be performed (see instructions in section 2 of the A5225/HiFLAC MOPS).

Additional CSF samples may be required based on participants’ response to study treatment. See section 6.3.22, below.

CSF collection and cultures must be performed at the Progression of Symptoms visit.

Wherever possible, CNS imaging should be performed at the same time that CSF samples are obtained for evaluation of progression of symptoms.

6.3.22 Possible CSF Cultures

Beginning at week 4 and continuing through week 10 (ie, at weeks 4, 6, 8, and 10), CSF must be collected and both qualitative and quantitative cultures must be performed for all participants whose previous quantitative culture was positive.

CSF collections should be performed on participants who prematurely discontinue study treatment only as clinically indicated; there are no subsequent study-required cultures for these participants.

6.3.23 CSF Cryptococcal Isolate Storage

CSF cryptococcal isolates must be obtained from all Cryptococcus-positive cultures. All CSF cryptococcal isolates should be stored as described in section 2 of the A5225/HiFLAC MOPS for future fluconazole susceptibility testing and sub-typing in a central laboratory.

6.3.24 PK Sampling (see section 10.0)

Section 10.2 contains specific collection and reporting requirements and should be consulted prior to any collection.

PK samples will be collected only from participants who receive **study-provided** fluconazole during Step 1. Participants who prematurely discontinue study-provided fluconazole prior to exiting Step 1 will not undergo PK evaluations at subsequent visits.

NOTE: Under the following conditions, the PK collection should be rescheduled to occur within 1 week.

- If the participant has missed any doses of fluconazole within the previous 3 days or on the day of collection
- If a participant taking EFV has missed any doses of EFV within the previous 3 days
- If a participant taking RIF daily has missed any doses of RIF within the previous 3 days or on the day of collection
- If a participant taking RIF less frequently than daily has missed any doses of RIF within the previous week

With the exception of the week 2 sample, the CSF collection, when an LP is clinically indicated, should also be rescheduled, when the collection of PK samples is postponed.

If it is not possible to reschedule the CSF collection to coincide with a rescheduled PK visit, a single PK sample should be collected within 1 hour of the CSF sample collection at the originally scheduled visit. If it is not possible to reschedule the PK visit, a single sample should be collected at the originally scheduled PK visit.

In addition, a single plasma sample for fluconazole levels will be collected at the time that Premature Treatment or Study Discontinuation evaluations are performed from all participants who prematurely discontinue the study or prematurely discontinue study treatment, except as noted in 6.2.4 for participants who are found to be HIV- and/or CM-negative.

6.3.25 Stored Plasma

An additional blood sample for plasma storage must be collected as indicated on the SoE and stored for possible future microbiologic, immunologic, and virologic evaluations. See section 2 of the A5225/HiFLAC MOPS and/or the A5225/HiFLAC LPC for collection and storage instructions.

6.3.26 Stored Serum

A serum sample will be collected **as indicated on the SOE** and stored for possible microbiologic evaluations.

6.3.27 Stored Urine

A urine sample will be collected at entry and at a Progression of Symptoms visit and stored for possible microbiologic evaluations.

6.3.28 Adherence Assessment

At each PK visit in which he/she takes part, each participant will be asked to report on adherence for that day and within the previous 3 days to all the drugs that he/she has been taking (ie, fluconazole, EFV, and/or RIF). See section 10.2 for reporting requirements.

In addition, at all other study visits, all participants will be asked to report adherence to study treatment within the previous 4 days.

## 7.0 CLINICAL MANAGEMENT ISSUES

A5225/HiFLAC sites must follow the DAIDS Table for Grading the Severity of Adult and Pediatric Adverse Events Version 1.0, December 2004, which can be found on the DAIDS DAIDS RSC Web site: <http://rsc.tech-res.com/safetyandpharmacovigilance/>, for grading toxicities related to study treatment.

Toxicity management at non-US CRSs may require reliance on clinical symptoms, clinician judgment, and available laboratory markers, since alternatives to study-provided treatment may be very limited and baseline levels may be different from those in other settings. It is understood that treating physicians will provide whatever available treatment is considered best to protect patient safety and well-being; compliance with study requirements must not compromise such treatment.

This section provides guidelines for management of toxicities related to **study-provided** fluconazole. Management of toxicities thought to be related to ampho B will be according to local standard of care. Guidelines for use of ampho B and management of the most common toxicities associated with it are included in section 5 of the A5225/HiFLAC MOPS and may be consulted, at the discretion of the site investigator.

Participants who permanently discontinue either fluconazole or **any** ampho B**-based regimen** because of an AE should be followed closely until resolution of the AE can be documented.

In the subsections below, ‘interruption’ is used to mean a temporary discontinuation, while ‘discontinuation’ is used to mean a permanent discontinuation. If study-provided fluconazole is interrupted for longer than 3 consecutive days, it must be discontinued and the participant must immediately be treated according to local standard of care (SoC).

The A5225/HiFLAC core team must be notified via e-mail ([actg.corea5225@fstrf.org](mailto:actg.corea5225@fstrf.org)) within 3 business days after premature discontinuation of fluconazole or ampho B in Step 1 or Step 2.

### 7.1 General Toxicities

The management of most fluconazole-related toxicities is summarized in this first section. Management of specific toxicities is addressed in the subsections that follow.

Grades 1 and 2: No change in treatment.

Grade 3 or 4: For a Grade 3 toxicity definitely, probably, or possibly related to fluconazole or for any Grade 4 toxicity, fluconazole should be reduced to 400 mg/day (or 600 mg/day if taking RIF) in Step1 and to 200 mg/day in Step 3. The reduced dose should be administered until toxicity resolves to a Grade ≤ 2 at which time fluconazole may either be escalated [e.g., to 400 mg/day (or 600 mg/day if taking RIF), 800 mg/day, and beyond, up to the assigned dose] or be resumed immediately at the assigned dose, at the discretion of the site investigator.

If the same Grade 3 or 4 toxicity recurs, fluconazole should be interrupted again and the A5225/HiFLAC core team should be contacted ([actg.corea5225@fstrf.org](mailto:actg.corea5225@fstrf.org)).

For a Grade 3 toxicity not related or probably not related to fluconazole, no interruption is necessary.

### Fluconazole-Induced Prolongation in QT Interval

The QT interval has an inverse relationship to heart rate; the measured QT intervals are generally corrected for heart rate in order to determine whether they are prolonged relative to baseline. The site is responsible for documenting any change relative to the baseline value.

Various correction formulae have been suggested, of which the Fridericia’s correction is most widely used. The correction factor used in this protocol is given below.

Fridericia’s correction: QTc = QT/RR^0.33^, or,

the QT interval divided by the cube root of the RR interval

Grade 1: Fluconazole should continue without interruption, at the discretion of the site investigator.

Grade 2: Fluconazole should continue without interruption, at the discretion of the site investigator. A repeat ECG should be considered 1 week later.

Grade 3: Fluconazole should be interrupted. If the Grade 3 QTc prolongation is considered to be most likely due to concomitant illness or medication, standard management, including discontinuation of the likely causative agent, should be undertaken. If the QTc prolongation resolves to Grade ≤ 1 within 3 days, the investigator should consider whether fluconazole can be re-started, as described in 7.1. If the QTc prolongation does not resolve to Grade ≤ 1 within 3 days, study-provided fluconazole should be discontinued and the participant referred to SoC.

Grade 4: Participants should permanently discontinue fluconazole.

### 7.3 Fluconazole-Induced Hepatotoxicity

Grade 1:     No interruption is necessary.  AST, ALT, alkaline phosphatase, and bilirubin evaluations must be repeated within 1 week.

Grade 2:     No interruption is necessary immediately, however, AST, ALT, alkaline phosphatase, and bilirubin evaluations must be repeated within 3 days. If the lab values are still Grade 2, fluconazole must be dose reduced as described in 7.1. If fluconazole is already being taken at the maintenance dose, it must be interrupted. The lab tests should be repeated within 3 days after dose reduction or interruption.

If the lab values do not decrease to Grade < 2 within 7 days following dose reduction, fluconazole must be discontinued and the participant should be referred to SoC.

If the lab values decrease to Grade < 2 within 7 days, then fluconazole may be restarted or dose-increased as described in section 7.1, at the discretion of the site investigator. AST, ALT, alkaline phosphatase, and bilirubin evaluations must be repeated within 1 week.

Grade 3:     If the toxicity is thought to be possibly, probably, or definitely related to fluconazole, then fluconazole must be interrupted. AST, ALT, alkaline phosphatase, and bilirubin evaluations must be repeated within 3 days.

If any of these lab values are still elevated 3 days following an interruption, fluconazole must be discontinued and the participant referred to SoC.

If the lab values decrease to Grade ≤ 2 within the timeframe noted above that allows for re-start, then fluconazole may be re-started as described in 7.1. AST, ALT, alkaline phosphatase, and bilirubin evaluations must be repeated within 1 week.

Grade 4:     If the toxicity is thought to be possibly, probably, or definitely related to fluconazole, then fluconazole must be interrupted. AST, ALT, alkaline phosphate, and bilirubin evaluations must be repeated within 2 days.

If any of these lab values are still Grade 4 3 days following an interruption, fluconazole must be discontinued and the participant referred to SoC.

If the lab values decrease to Grade ≤ 2 within the timeframe noted above that allows for re-start, fluconazole may be restarted as described in 7.1. AST, ALT, alkaline phosphate, and bilirubin evaluations must be repeated within 1 week.

Among participants with Grade ≥ 3 liver abnormalities in whom fluconazole-associated toxicity is highly unlikely in the opinion of the investigator, fluconazole may be continued without interruption. The investigator should document a definitive alternative etiology if the relationship of toxicity to fluconazole is determined to be highly unlikely.

NOTE: Participants with isolated Grade ≥ 3 hyperbilirubinemia resulting from use of indinavir or atazanavir do not need to have their fluconazole interrupted.

### 7.4 Fluconazole Dose Modifications

- - 1. Renal Impairment

The initial fluconazole dose will be given as specified, independent of the screening creatinine clearance. Subsequently, doses **of study-provided fluconazole** should be adjusted according to the table below for subjects with decreased creatinine clearance. **Dose adjustments to fluconazole that is given in combination with ampho B are also important; refer to the package insert for guidance.**

Table 7-1

| Creatinine Clearance  (Measured or Estimated) | Fluconazole Dose |
| --- | --- |
| ≥ 50 mL/min | current dose |
| 20 - 49 mL/min | 50% current dose |
| 0 – 19 mL/min | 25% current dose |

- - 1. Body Weight < 60 kg at Study Entry

The following adjustments should be made in the induction dose of fluconazole for participants whose weight at entry is < 60 kg. All adjusted doses of fluconazole should be rounded up to the nearest 200 mg, based on the participant’s actual weight. For example, if a participant in Cohort 1 weighs 42 kg, 42 kg X 20 mg/kg = 840 mg; dose should be rounded up to 1000 mg. Weight-based adjustments must NOT be made to any consolidation or maintenance doses of fluconazole.

Table 7-2

| Weight at Entry | Weight-Adjusted Daily Fluconazole Dose for Induction Step | | |
| --- | --- | --- | --- |
|  | For 1200 mg cohorts | For 1600 mg cohorts | For 2000 mg cohorts |
| < 60 kg | 20 mg/kg up to 1200 mg total | 26 mg/kg up to 1600 mg total | 33 mg/kg up to 2000 mg total |

### 7.5 Fluconazole-Induced Rash

Grade 1 or 2: Fluconazole should continue without interruption, at the discretion of the site investigator. Participants with a Grade 1 or 2 rash may be treated symptomatically with permitted antipyretic, antihistamine, and/or non-steroidal anti-inflammatory medications, but should be monitored closely by the site investigator.

Grade 3: Participants should interrupt fluconazole. If the Grade 3 rash is considered to be most likely due to concomitant illness or medication, standard management, including discontinuation of the likely causative agent, should be undertaken. If the rash resolves to Grade ≤ 1 within 3 days following an interruption, the investigator should consider whether re-starting fluconazole, as described in section 7.1, is appropriate.

Grade 4: In case of exfoliation, mucosal involvement, or target lesions (erythema multiforme), or any evidence of Stevens-Johnson syndrome or toxic epidermal necrolysis (TEN), participants should permanently discontinue fluconazole.

### 7.6 Adrenal Insufficiency (AI)

Fatigue, weakness, loss of appetite, nausea, and vomiting with orthostatic hypotension may be signs of secondary AI. It is unusual to have the low sodium and potassium levels that are frequently seen in primary AI. Causes of AI may be secondary to cryptococcal infection and typically resolve with treatment of the infection. However, AI may also be related to high doses of fluconazole. Participants who develop these symptoms while taking fluconazole may need further evaluation for secondary AI (eg, cortisol stimulation test, if available). Treatment with prednisone or a similar glucocorticosteroid may be indicated. Note that cortisol levels in samples collected early in the day tend to be less than 3 mcg/dL; ACTH levels are low in the setting of secondary AI.

### 7.7 Pregnancy

Women who become pregnant on study while receiving fluconazole must discontinue fluconazole immediately. The core team should be notified as soon as is reasonably possible.

If a woman has completed the study or chooses to discontinue from the study before the end of the pregnancy, then site staff should request permission to contact her regarding pregnancy outcomes at the end of pregnancy. If the information is obtained, pregnancy outcomes will be submitted on a CRF at the end of the pregnancy.

Pregnancies that occur on study in women who are taking ARVs will be reported prospectively to The Antiretroviral Pregnancy Registry. Intrapartum complications and/or pregnancy outcome will be recorded on the CRFs and also reported to The Antiretroviral Pregnancy Registry. More information is available at [www.apregistry.com](http://www.apregistry.com/). Phone: 800-258-4263; Fax: 800-800-1052. At sites outside the US, report pregnancies to The Antiretroviral Pregnancy Registry by Fax at: +44-1895-825-005, or by phone at: 910-256-0238.

## 8.0 CRITERIA FOR PREMATURE TREATMENT OR STUDY DISCONTINUATION

### 8.1 Treatment Discontinuation

- **Failure to have CM confirmed by Day 5 by testing performed at a DAIDS-approved laboratory**
- Fluconazole-related dose-limiting or ampho B-related treatment-limiting toxicity (see section 7.0, Clinical Management Issues or section 5.0, A5225/HiFLAC MOPS).
- Pregnancy or breast-feeding (for fluconazole).
- Interruption of oral fluconazole for any reason for more than 3 consecutive days.
- Requirement for prohibited concomitant medications (see current A5225/HiFLAC MOPS).
- Clinical reasons believed life threatening by the physician, even if not addressed in the clinical management section of the protocol.

### Study Discontinuation

- Negative screening CSF culture and/or confirmatory HIV status screening results

**A negative screening CSF culture is defined as**

- **a negative culture after 2 weeks of incubation even in the setting where the CSF antigen testing is positive by latex agglutination from a referring laboratory**

**or**

- **a negative culture after 5 days of incubation if there is no yeast found on culture and there is a negative CSF antigen test by the latex agglutination method by Day 5 from an approved laboratory.**.
- Failure to obtain a quantitative CSF culture at screening or entry.
- Failure to initiate study treatment within 72 hours after entry.
- Initiation of NVP prior to fluconazole dose reduction to ≤ 400 mg daily
- Request of the primary care provider if s/he thinks the study is no longer in the best interest of the participant.
- Failure by the participant to attend three consecutive clinic visits.
- Request by the participant to withdraw.
- Participant judged by the investigator to be at significant risk of failing to comply with the provisions of the protocol as to cause harm to self or seriously interfere with the validity of the study results.
- At the discretion of the Office for Human Research Protections (OHRP), Food and Drug Administration (FDA), NIAID, ACTG [including the Study Monitoring Committee (SMC)], local IRB/EC or other regulatory authority, industry supporter, or investigator.

## 9.0 STATISTICAL CONSIDERATIONS

### 9.1 General Design Issues

Stage 1 of A5225/HiFLAC is a phase I/II dose escalation study of the safety and tolerability of an inuction-consolidation strategy of high-dose fluconazole alone for the treatment of CM in HIV-infected participants. Dose escalation will occur between fluconazole cohorts but not within them. Escalation and determination of the MTD will be based on the rate of DLT, as defined in the Glossary of Study-Specific Terms. When safety has been evaluated in Stage 1, additional participants will be enrolled in Stage 2 to one or more doses at or below the MTD to provide additional information on safety and to identify the optimum (best safety and efficacy profile) dose of fluconazole. If more than one dose is to be evaluated, participants will be randomized between fluconazole dose levels in Stage 2. Decisions about escalation between fluconazole cohorts in Stage 1 will be made after the last enrollee in a cohort has been observed for at least 14 days or until it has been confirmed whether or not that participant has experienced DLT. The decision about which doses to study in Stage 2 will be made after all participants in cohort(s) with acceptable DLT have been followed for at least 10 weeks. The decision rules for Stage 1 and the definition of the MTD are detailed in sections below.

In parallel with the fluconazole escalation and efficacy portions of the study, participants will be randomized to standard treatment with an ampho B-based regimen. During Stage 1, the randomization will be 3:1 between fluconazole and an ampho B-based regimen, with 24 participants enrolled in each of the fluconazole cohorts tested. An additional participant will be enrolled to replace each participant excluded for: (a) not being infected with HIV-1; (b) not having a CM positive CSF culture at entry; or (c) refusal/withdrawal of consent prior to taking any study medication, if the participant's Stage 1 cohort is still open to enrollment. Thus, assuming all three fluconazole cohorts are tested, 24 participants will be treated with ampho B in Stage 1. In Stage 2, an additional 24 participants will be enrolled in each of the fluconazole candidate doses identified in Stage 1 based on their safety and efficacy profiles. The randomization ratio between fluconazole and ampho B-based regimens in Stage 2 will depend on how many doses will be evaluated, with the aim of achieving a total of 48 participants receiving ampho B between Stages 1 and 2.

Because of the need to pause between doses in Stage 1 and to pause long enough to confirm efficacy prior to initiating Stage 2, it is difficult to estimate the duration of accrual on this study. We anticipate that accrual may be completed approximately 1 year after all eligible sites are able to begin enrolling. However, pauses between doses in Stage 1 must be factored in.

### 9.2 Outcome Measures

9.2.1 Primary Outcomes

9.2.1.1 Discontinuation of **study-provided** fluconazole or ampho B, including precipitating and surrounding adverse events.

9.2.1.2 Qualitative and quantitative CSF culture results at entry, week 2, and when conducted thereafter.

9.2.1.3 Survival.

9.2.2 Secondary Outcomes

9.2.2.1 Results of the neurological examination (at study entry and at weeks 2, 10, 24, at progression of symptoms, and at the time of premature treatment or study discontinuation) and of functional status evaluation (prior to onset of current CM illness, at study entry, at week 10, and at time of premature treatment or study discontinuation).

9.2.2.2 Length of hospitalization and number and nature of hospital readmissions.

9.2.2.3 Recurrence/relapse of CM based on clinical presentation.

9.2.2.4 CNS IRIS.

9.2.2.5 Additional safety parameters including: Grade 3 and 4 adverse events; dose modifications; duration of temporary treatment interruptions; permanent discontinuation of either agent.

9.2.3 Exploratory Outcomes (to be described in separate analysis plans)

9.2.3.1 Pharmacology (see section 10.0).

9.2.3.2. Antifungal drug susceptibility of cryptococcal isolates.

9.2.3.3 Ability of LFCrAg assay to detect cryptococcal antigen in stored urine, serum, plasma, and CSF.

### 9.3 Sample Size and Criteria for Stages 1 and 2

9.3.1 Stage 1

The team feels that standard dose (400 mg/day) fluconazole would have about 5% DLT as defined in this study. To avoid the toxicity and difficulty of administration of ampho B, the team feels that ≤25% DLT would be acceptable. In the international setting and in participants with HIV and AIDS, the team also has concluded that there likely will be up to a 15% very early mortality rate in any cohort due to CM and/or HIV, not to the dose of fluconazole. Including participants who received very little fluconazole in safety evaluations to determine dose escalation could bias the evaluation either to be too high (if early deaths without toxicity are considered to be DLTs) or too low (if early deaths without toxicity are included in the denominator of the DLT rate). Thus, for primary safety evaluations, very early deaths (prior to Day 4) will not be included in the decision rule whether they experienced a DLT prior to early death or not [French, 2002; Okongo, 1998; Hakim, 2000; van der Horst, 1997; Brouwer, 2004; Pappas, 2004; Bicanic, Meintjes, et al, 2008; and Milefchik, 2008]. Should more than 6 very early deaths be seen in a cohort, enrollment to the cohort will by paused while the team evaluates the events carefully in conjunction with the SMC to consider if there should be study modifications. Secondary evaluations prior to escalation will include all subjects in a failure-time analysis of DLT with early deaths censored if DLT did not occur prior to death.

The dose escalation rule to be used in Stage 1 is:

- 0-3 DLTs; escalate to the next higher dose.
- 4-9 DLTs; do not escalate. This is the MTD.
- > 9 DLTs; the prior dose is the MTD.

Should > 9 DLTs be observed before a cohort is fully accrued, accrual to that cohort will be suspended pending review. The derivation of this rule is based on calculations for a wide range of potential evaluable sample sizes, escalation rules, and scenarios about the rates of DLT at the three fluconazole doses. The following table provides the study-wide operating characteristics of this rule for an example of DLT rates across the three doses if there are 20 or 22 subjects evaluable at each dose, where the “average” DLT was calculated as the weighted average of the underlying rates of DLT.

Table 9-1

| True DLT  1200,1600, 2000 | Prob 1200 Is  > MTD | Prob  1200 Is MTD | Prob  1600 Is MTD | Prob 2000 Is MTD | Average  DLT |
| --- | --- | --- | --- | --- | --- |
| N=20/dose |  |  |  |  |  |
| 0.05, 0.10, 0.20 | 0.000 | 0.016 | 0.139 | 0.845 | 0.184 |
| 0.10, 0.20, 0.30 | 0.000 | 0.142 | 0.542 | 0.316 | 0.217 |
| 0.20, 0.30, 0.40 | 0.010 | 0.625 | 0.338 | 0.026 | 0.237 |
| N=22/dose |  |  |  |  |  |
| 0.05, 0.10, 0.20 | 0.000 | 0.022 | 0.184 | 0.794 | 0.178 |
| 0.10, 0.20, 0.30 | 0.000 | 0.189 | 0.588 | 0.223 | 0.203 |
| 0.20, 0.30, 0.40 | 0.020 | 0.710 | 0.260 | 0.010 | 0.224 |

Thus, for example, if 22 participants are evaluable at each dose level and the underlying true DLT rates are 10, 20 and 30% at 1200, 1600, and 2000 mg doses, there is a 19% chance that 1200 mg will be found to be the MTD, a 59% chance that 1600 will be found to be the MTD, and a 22% chance that 2000 will be found to be the MTD in Stage 1. Stage 2 will provide additional information on safety that will refine these findings.

Assuming exclusion of up to 15% very early deaths from a cohort, accrual of 24 participants at each dose level will yield 20-22 evaluable participants at each fluconazole dose level. Application of the same escalation rule above regardless of the sample size within this range provides consistent escalation probabilities and adjustment to the rule to reflect the number of evaluable participants is not necessary. The dose rule also maintains an average DLT below 25% given the scenarios presented in Table 9-1 should a dose level have more (or fewer) evaluable participants than 20-22. Additionally, as noted previously, DLT will also be evaluated with all participants using a censored approach.

To provide additional detail on this component of the study, the following tables show the within-cohort probabilities of escalation, determination that a current dose is the MTD, or that the prior dose is the MTD for N=20 or 22 evaluable participants at a dose and a range of true underlying rates of DLT.

Table 9-2

|  | .05 | .10 | .20 | .30 | .40 | .50 |
| --- | --- | --- | --- | --- | --- | --- |
|  |  |  |  |  |  |  |
| N=20 |  |  |  |  |  |  |
| Prob(Escalate) | .984 | .867 | .411 | .107 | .016 | .001 |
| Prob(this MTD) | .016 | .133 | .579 | .780 | .580 | .250 |
| Prob(prior MTD) | .000 | .000 | .010 | .113 | .404 | .748 |
|  |  |  |  |  |  |  |
| N=22 |  |  |  |  |  |  |
| Prob(Escalate) | .978 | .828 | .332 | .068 | .008 | .000 |
| Prob(this MTD) | .022 | .172 | .662 | .840 | .617 | .261 |
| Prob(prior MTD) | .000 | .000 | .006 | .092 | .376 | .738 |
|  |  |  |  |  |  |  |

Simultaneous randomization to an ampho B-based regimen during Stage 1 will provide approximately 24 concurrently treated participants for preliminary information on underlying safety, mortality, and efficacy with this arm in an international multi-center setting. This will also ensure that participants in the combined ampho B data span the time of accrual to fluconazole, and reduce bias that might occur should all participants receiving ampho B be accrued only during Stage 2 when sites and participants are aware of the general outcome of Stage 1.

The rate and nature of deaths on Stage 1 will be carefully reviewed on an on-going basis. As noted below, should more than 6 very early deaths be seen in a fluconazole cohort, the team will evaluate the events carefully in conjunction with the SMC to consider if there should be study modifications. There is a 6% chance of a review being triggered if the true early mortality rate is 15% as anticipated, a 61% chance of review if the underlying rate is 30%, and a 90% chance of a review being triggered if the underlying rate is 40%.

Stage 1 can provide preliminary guidance on efficacy for the choice of cohorts to move to Stage 2. With 20-22 subjects with evaluable quantitative culture results over time per group, a difference in mean change in Cryptococcus (expressed as log_10_ CFU/ml CSF/day) of about 80% of the standard deviation of the measure can be detected with 90% power using a relaxed one-sided 0.10 alpha-level test. For example, if EFA (early fungicidal activity defined as the change from day 0 to day 14) is -0.30 with a standard deviation (s.d.) of 0.15 log_10_ CFU/ml CSF/day, an increase in the clearance to about -0.42 can be detected.

We recognize that the many neurological complications of CM may obscure drug-related ones. We will monitor participants for unusual neurological manifestations of CM that might be attributed to fluconazole. With 20 (22) evaluable participants, there is a 64% (68%) chance of seeing at least one episode of a completely unexpected unacceptable event that has a true underlying rate of occurrence of 5%. Should we see one or more DLTs associated with cardiac toxicity at a dose level, a targeted review will be triggered. This will happen with 88% probability if the underlying rate of cardiac toxicity is 10%. Should we see 2 or 3 DLTs in 20-22 participants at a dose level, and 2 of 2 or 2-3 of 3 are associated with hepatic toxicity, a targeted review will be performed before the dose can be escalated to the next level. As noted in 9.3.2, the nature and rates of all events will be evaluated prior to accepting any dose for further testing in Stage 2.

9.3.2 Stage 2

The decision about which doses will be chosen for testing in Stage 2 will be based on: Stage 1 toxicity including the rates of DLT and also the grades and types of toxicity, dose modifications, the duration of treatment interruptions, and the rate of permanent treatment and study discontinuations, and general consideration of efficacy including early CM mortality, CNS clearance (both the rate of clearance and the proportion who clear), and early information on the duration of remission. An additional 24 participants will be randomized to each dose of fluconazole carried into Stage 2. The Stage 2 sample size for ampho B-based regimens will depend on how many fluconazole doses were tested in Stage 1 with the intent of having a total of 48 participants treated with ampho B between the two Stages. The randomization ratio will depend on how many fluconazole doses are tested and on the target ampho B sample size in this stage.

The target sample size of a total of 48 participants on a dose of fluconazole between Stages 1 and 2, and 48 on an ampho B-based regimen is based on:

- Ensuring that the width of a 90% confidence interval (CI) for culture negativity (early discontinuation or death = fail) within a dose or within ampho B-based regimens no wider than 25%.
- Detecting a difference in EFA of about 0.10 log10 CFU/ml CSF/day (about 67% of the s.d.) between two fluconazole doses or between a dose of fluconazole and ampho B with 90% power and a 2-sided 0.05-level test.
- Detecting a 25% difference (e.g. 50% vs. 75%) in efficacy between two fluconazole doses or between a dose of fluconazole and ampho B with 90% power (one-sided alpha=.10 test).
- Having the added ability to detect unusual or unexpected events that occur at a low rate, especially in consideration of those who might not be evaluable for toxicity given very early death. For example, if an unexpected and unacceptable event occurs at a rate of 5%, there is only a 64% chance of seeing one or more rare events in 20 participants evaluable for safety and an 87% chance of seeing one or more rare events in 40 participants evaluable for safety combined over both Stages.

Note: Because of the pilot nature of this study and the considerations of both a wide range of safety information and early efficacy that will guide the doses to be studied in Stage 2, data from the two stages will be combined for evaluations of efficacy without adjustment for the two-stage design. Overall considerations of DLT will be adjusted for the two-stage design: as a guide to the operating characteristics of a Stage 1/Stage 2 analysis of safety, the upper bound of the CI around observed safety will be lower than the naïve interval because of the 2-stage. For example, if 44 participants are evaluable for safety and 8 events of a specific type are noted, the upper 90% naïve CI is .304, and the CI is the same with adjustment [Atkinson, 1985]. However, adjustment for Stage 1 will lower the upper (and lower) bound.

- - 1. Secondary Endpoints

Analyses of secondary endpoints and objectives will not be adjusted for the 2-stage design or for multiple testing. Some will evaluate data combined (eg, all participants receiving fluconazole vs. all receiving an ampho B-based regimen), and some will consider fluconazole dose and match with concurrently enrolled ampho B cases. The sample size for the drug susceptibility testing may depend on site capability to perform the test or to ship to a site with that capability. The following table presents the power to detect effect sizes for dichotomous (percent CM relapse; proportion with DLT or death; proportion with low drug susceptibility; components of the neurologic testing) and normally distributed secondary endpoints (eg, log_10_ viral loads and CD4 counts; components of the neurologic testing). The power shown here is for one-sided 0.05-level tests. The ‘sidedness’ of each question will be decided a priori and stated in the detailed A5225 Analysis Plan that will be developed before any analyses of secondary endpoint data are initiated.

Table 9-3

Power for between-group tests Group sizes

| Distribution | Effect size | 24-24 | 24-48 | 48-48 | 48-96 |
| --- | --- | --- | --- | --- | --- |
| Dichotomous | 20% vs. 40% | 34% | 47% | 61% | 73% |
|  | 20% vs. 50% | 60% | 75% | 90% | 97% |
|  |  |  |  |  |  |
| Normal | .4 x s.d. | 39% | 48% | 62% | 73% |
|  | .6 x s.d. | 66% | 77% | 90% | 96% |
|  | .8 x s.d. | 86% | 94% | 99% | <99% |

The precision with which the rate of IRIS can be estimated will depend on the proportion of participants who initiate ART and the time on study between ART initiation and when participants go off study. It should be remembered that this study is relatively short, and it is recommended that ART not be initiated until Step 3 or 4. The time from ART start to development of IRIS (i.e., any IRIS or specifically CNS IRIS) and the proportion with IRIS at pre-specified time points such as 8 or 12 weeks will be estimated by Kaplan Meier methods. The 95% CI on the proportion with IRIS at specified time points will be calculated using Greenwood’s formula.

### 9.4 Monitoring

- - 1. Stage 1 Monitoring

Stage 1 cohorts will be monitored by the core safety team, which will consult with an ACTG-appointed SMC for an unblinded review of each cohort’s safety data prior to escalation to the next cohort. This review will include data from at least 14 days after entry of the last participant on a cohort. The study may be referred to an SMC during Stage 1 as outlined below. The core team will consult with the SMC for an unblinded review of Stage 1 safety and efficacy to choose the fluconazole dose(s) to be evaluated in Stage 2. There will be regular reports of AEs by dose level to the DAIDS clinical representative following the standard reporting schedule for phase I/II studies, and regular reports on accrual and data quality to the entire team.

This study will require rapid assessment of safety and treatment discontinuations, so will use 48-hour form reporting for all AEs and dose modifications and discontinuations. Standard phase I toxicity reports will be distributed frequently to the core safety team that consists of the study chairs, DAIDS clinical representative, statisticians, data manager, and CTS. The core safety team will have periodic calls to review the data, with the frequency depending on the rate of enrollment into the study and the number and type of events reported. Any member of the core safety team may call for an expedited call. The core safety team will decide whether an event is to be considered a DLT. If the core safety team does not unanimously agree, then the event will provisionally be included in the count of DLTs for determining induction regimen escalation. If the number of provisional events prior to full accrual of a cohort impacts the decision about the safety of a particular fluconazole induction dose level, the decision on dose safety will be referred to the SMC for a final decision and enrollment will be suspended until the SMC decision is received. The core safety team might consult with the SMC at other times during a cohort’s enrollment and follow up and may suspend enrollment pending the outcome of such a review. Additionally, should more than 6 very early deaths be seen in a cohort (or a concerning proportion seen early in cohort accrual), or should we see 2 or 3 DLTs in 20-22 participants at a dose level, and 2 of 2 or 2-3 of 3 are associated with hepatic toxicity, the team will evaluate the events carefully in conjunction with the SMC to consider whether there should be study modifications. The SMC and core team will review safety data and the SMC will review preliminary efficacy data prior to opening a new Stage I cohort for accrual.

9.4.2 Stage 2 Monitoring

Safety in Stage 2 of this study will be monitored by the core safety team as above. Data on fluconazole doses will be pooled for core safety team monitoring. Data on ampho B safety will be presented separately. There will be regular reports of AEs (pooled over fluconazole doses) to the DAIDS clinical representative following the standard reporting schedule for phase I/II studies and regular reports on accrual and data quality to the entire team. This study will be reviewed by the SMC at least annually after entry of the first participant on Stage 2.

### 9.5 Analysis

9.5.1 Stage 1

Dose escalation is based on DLT and the decision rules as outlined above. As noted above, between-dose comparisons to select the fluconazole dose(s) to carry forward will largely be descriptive and not formally powered as all information will be factored into this decision.

9.5.2 Stage 2

In this pilot, we intend to combine the data over both Stages for the ampho B-based cohorts and for fluconazole doses that are tested in Stage 2. As noted above, only the confidence interval around the observed DLT rate in each dose will be adjusted for the dose escalation scheme. Analyses will employ appropriate methods such as Fisher’s exact test to compare dichotomous outcomes (e.g., Grades 3 and 4 events; CSF clearance before week 10) between doses or drugs, and t-tests or Wilcoxon tests to compare continuous covariates between two groups (or ANOVA or Kruskall-Wallis tests if three groups including ampho B). In the event all three fluconazole doses are evaluated in Stage 2, tests for trend such as ANCOVA, Cochran-Armitage and Jonckheere-Terpstra tests will be performed. Survival and other measures of time-to-event from study entry will be summarized with Kaplan-Meier estimates and, where appropriate, tested with log rank tests and possibly proportional hazards regression. The duration of the initial hospitalization will need to consider the duration of hospitalization preceding study entry. The focused neurological exam will be analyzed both on an item-by-item basis and also as a summary measure using methods such as mixed models analysis of variance or GEE that accommodate missing data and changing covariates.

The content and schedule of reports to be available to the core team, the SMC, and the full team will be detailed in the Study Monitoring Plan that will be developed prior to the opening of accrual to the study.

## 10.0 PHARMACOLOGY PLAN

**Pharmacology evaluations will be conducted only on samples collected from participants receiving study-provided fluconazole for the induction phase of treatment.** The pharmacology plan is described under the assumption that two of the three **Step 1** fluconazole **induction** doses tested in Stage 1 will be tested in Stage 2. Should all three doses be tested in Stage 2, appropriate modifications will be made to the hypotheses, and more modeling may be performed across the dose range. Should only one dose be tested in Stage 2, some analyses (eg, multicovariate modeling) will not be pursued. However, between-dose testing, albeit with lower power, could elucidate why only one dose had evidence of both tolerability and efficacy in Stage 1. Because of the possibility of drug-drug interactions with RIF and/or EFV, a number of objectives are restricted to being evaluated in samples from participants who did not receive these drugs. The objectives confined to those receiving RIF and/or EFV will only be pursued if sufficient numbers of participants receive these drugs and have the appropriate samples collected from them.

### 10.1 Pharmacology Objectives

10.1.1 Primary Objective

Compare concentrations of fluconazole in plasma and CSF at steady state for the doses tested in Stage 2. Samples from participants taking RIF or EFV at the week 2 PK collection will not be used in this comparison.

10.1.2 Secondary Objectives (for samples from participants who were not taking RIF or EFV at the week 2 PK collection)

10.1.2.1 Evaluate the relationship of fluconazole dose to fluconazole trough concentrations in plasma and CSF at steady state across the range of all induction doses studied.

10.1.2.2 Evaluate the relationship between fluconazole plasma and CSF concentrations in the doses tested in Stage 2 and change in cryptococcal concentration in CSF from baseline to week 2 and time to CSF sterility.

10.1.2.3 Evaluate the relationship between fluconazole plasma and CSF concentrations in the doses tested in Stage 2 and fluconazole toxicity or intolerance independent of dose as defined by Grade ≥ 3 toxicity, change in dose, or discontinuation of fluconazole.

10.1.2.4 Evaluate the relationship between fluconazole dose and plasma concentrations before and after dose reductions for toxicity or intolerance in the doses tested in Stage 2 (within-participant dose-to-plasma concentration comparisons).

10.1.2.5 Evaluate the relationship of fluconazole plasma and CSF concentrations with dose and demographic and physiological characteristics of participants including, but not limited to sex, weight, body mass index, and creatinine clearance.

10.1.2.6 Evaluate relationship of self-reported adherence to plasma and CSF trough fluconazole concentrations and to efficacy.

10.1.3 Secondary Objectives (for samples from participants who were taking RIF or EFV at the week 2 PK collection)

10.1.3.1 Evaluate plasma and CSF concentrations of fluconazole alone versus fluconazole when given concomitantly with RIF in the 1600 mg and 2000 mg daily doses, if sufficient numbers of samples are available.

10.1.3.2 Evaluate plasma and CSF concentrations of fluconazole alone at the 1600 mg and 2000 mg daily doses versus fluconazole given concomitantly with EFV, if sufficient numbers of samples are available.

### 10.2 Pharmacology Study Design

10.2.1 Single sample for fluconazole at week 2

Plasma: At week 2, all participants in fluconazole induction cohorts will have whole blood collected for drug concentrations of fluconazole. For participants taking fluconazole once daily, the collection should take place 15-33 hours after the last dose of fluconazole and prior to the next dose of fluconazole. For participants taking fluconazole twice daily, the collection should take place 8-16 hrs after the last dose of fluconazole and prior to the next dose of fluconazole. For participants taking fluconazole more frequently than twice a day, sites should attempt to make the collection at least 4 hours after the most recent dose. The plasma PK sample should be obtained within 1 hour (ie, before or after) of the CSF collection described below.

CSF: A CSF sample must be obtained from all participants described in the paragraph above.

The date and time of the administration of all fluconazole doses for the previous 3 days and the day of the study visit as well as the date and time of plasma and CSF sampling will be recorded on the CRF.

- - 1. Single sample for fluconazole in participants taking RIF or EFV

Plasma: Whole blood will be collected for fluconazole concentrations and concentrations of the concomitant drug from all participants in the 1600 mg and 2000 mg daily fluconazole cohorts who are taking RIF and/or EFV (with no concomitant NVP) during the induction step. This collection should take place at least 2 weeks after the initiation of co-administration of fluconazole with the concomitant drug. For participants taking fluconazole once daily, the collection should take place 15-33 hours after the last dose of fluconazole and prior to the next dose of fluconazole. For participants taking fluconazole twice daily, the collection should take place 8-16 hrs after the last dose of fluconazole and prior to the next dose of fluconazole. For participants taking fluconazole more frequently than twice a day, sites should attempt to make the collection at least 4 hours after the most recent dose. The plasma sample should be obtained within 1 hour (i.e., before or after) of the CSF when CSF is collected as described below.

CSF: In addition, a CSF sample will be obtained for drug concentrations (if an LP is performed for other clinical or study purposes) from all participants taking part in this PK collection.

The following will be recorded on the CRF:

- date and time of all plasma and CSF samplings
- date and time of the administration of all fluconazole doses for the previous 3 days and the day of the study visit
- date and time of day (or actual or approximate time, if available) of all EFV doses for the previous 3 days and the day of the study visit (for participants taking EFV);
- date and time of day (or actual or approximate time, if available) for all RIF doses in the previous 3 days and the day of the study visit (for participants taking RIF daily);
- date and time of day (or actual or approximate, if available) for the last RIF dose including the day of the study visit (for participants taking RIF less frequently than daily).
  - 1. Single sample for fluconazole before and after dose reduction due to intolerability/toxicity

Plasma: All participants in the 1600 mg and 2000 mg daily fluconazole cohorts who require dose reduction of fluconazole due to intolerability/toxicity will have whole blood collected for fluconazole concentration at the time of the assessment of intolerability and prior to dose reduction. This sample can be obtained at any time during the induction step after the previous dose of fluconazole.

An additional sample will be collected at least 2 weeks after the dose reduction and while the participant is still taking the reduced dose. This sample may be obtained during a scheduled study visit. If the dose has resumed to full-dose, no sample should be taken for PK.

For participants taking fluconazole once daily, the collection should take place 15-33 hours after the last dose; for participants taking fluconazole, more frequently, the collection should take place 8-16 hrs after the last dose. For participants taking fluconazole more frequently than twice a day, sites should attempt to make the collection at least 4 hours after the most recent dose. The PK sample should be obtained within 1 hour (i.e., before or after) of the CSF sample described below is obtained.

CSF: In addition, a CSF sample will be obtained for drug concentrations from any participants involved in these PK collections for whom an LP is being performed for other clinical or study purposes.

For each sample, the date and time of the administration of all fluconazole doses for the previous 3 days and the date and time of blood and CSF sampling will be recorded on the CRF.

10.2.4 Single sample at time of Premature Treatment or Study Discontinuation

A single plasma sample for fluconazole levels will be collected at the time that Premature Treatment or Study Discontinuation evaluations are performed, except as noted in 6.2.4 for participants who are found to be HIV- and/or CM-negative.

10.2.5 Single sample at time of Progression of Symptoms

A single plasma sample for fluconazole levels will be collected at the time that evaluations for Progression of Symptoms are performed.

### 10.3 Primary and Secondary Data Analysis

The primary analysis will be a two-group t-test comparing the natural-log transformed plasma and CSF concentrations between all evaluable participants taking the doses tested in Stage 2 (i.e., participants taking these doses in Stages 1 and 2).

Secondary analyses will use various generalized linear models (e.g., standard regression for assessing impact of body mass index (BMI), race, gender, etc. on the trough concentrations; logistic regression for assessing impact of trough concentrations on toxicity, etc).

### 10.4 Adequacy of Sample Size

We anticipate the availability of at least 1 plasma sample and at least 1 CSF specimen from approximately 40 Stage 1 and Stage 2 participants in each of the dose cohorts tested in Stage 2, allowing for sites unable to participate in this aspect of the study. We show power calculations allowing for 5 participants in each cohort that might be receiving RIF or EFV at the week 2 draw or who might not have sufficient CSF available for PK analysis. Assuming a 25% coefficient of variance (CV) within a dose level, samples from 35 participants would be adequate to detect a difference of approximately 20% of the lowest of the two means between the two dose concentrations with 90% power (alpha=0.05, two-sided).

For secondary analyses, with 70 observations available, we should be able to assess the impact of 3-4 factors in addition to dose level in a linear model.

### 10.5 Anticipated Outcomes

We anticipate that the concentrations of fluconazole at the high doses planned in this study will remain consistent with the linear patterns of increased concentrations in both plasma and CSF that have been observed at the lower doses. We believe from the CCTG study that high doses may sterilize cryptococci in the CSF sooner or more frequently and that this will be related to higher levels of drug in the CSF. In addition, we anticipate a potential two-way drug interaction between fluconazole and EFV as well as between fluconazole and RIF. To our knowledge, fluconazole at the doses used in this investigation have not been evaluated with EFV coadministration. Likewise, the combination of RIF with these doses of fluconazole has not been studied.

## 11.0 DATA COLLECTION AND MONITORING AND ADVERSE EVENT REPORTING

### 11.1 Records to Be Kept

Case report forms (CRFs) will be provided for each participant. Participants must not be identified by name on any CRFs. Participants will be identified by the patient identification number (PID) and study identification number (SID) provided by the ACTG Data Management Center upon enrollment.

### 11.2 Role of Data Management

11.2.1 Instructions concerning the recording of study data on CRFs will be provided by the ACTG Data Management Center. Each site is responsible for keying the data.

11.2.2 It is the responsibility of the ACTG Data Management Center to assure the quality of computerized data for each ACTG study. This role extends from protocol development to generation of the final study databases.

### 11.3 Clinical Site Monitoring and Record Availability

11.3.1 Site monitors under contract to the NIAID will visit participating clinical research sites to review the individual participant records, including consent forms, CRFs, supporting data, laboratory specimen records, and medical records (physicians’ progress notes, nurses’ notes, individuals’ hospital charts), to ensure protection of study participants, compliance with the protocol, and accuracy and completeness of records. The monitors also will inspect sites’ regulatory files to ensure that regulatory requirements are being followed and sites’ pharmacies to review product storage and management.

11.3.2 The investigator will make study documents (e.g., consent forms, drug distribution forms, CRFs) and pertinent hospital or clinic records readily available for inspection by the local IRB/EC, the site monitors, the FDA, the NIAID, the OHRP, or the industry supporter or the supporter’s designee for confirmation of the study data.

### 11.4 Expedited Adverse Event (EAE) Reporting

11.4.1 Adverse Event Reporting to DAIDS

Requirements, definitions and methods for expedited reporting of Adverse Events (AEs) are outlined in Version 2.0 of the DAIDS EAE Manual, which is available on the RSC website at <http://rsc.tech-res.com/safetyandpharmacovigilance/>.

The DAIDS Adverse Experience Reporting System (DAERS), an internet-based reporting system must be used for expedited AE reporting to DAIDS. In the event of system outages or technical difficulties, expedited AEs may be submitted via the DAIDS EAE Form. For questions about DAERS, please contact DAIDS-ES at [DAIDS-ESSupport@niaid.nih.gov](mailto:DAIDS-ESSupport@niaid.nih.gov). Site queries may also be sent from within the DAERS application itself.

Where DAERS has not been implemented, sites will submit expedited AEs by documenting the information on the current DAIDS EAE Form. This form is available on the RSC website: <http://rsc.tech-res.com/safetyandpharmacovigilance/>. For questions about EAE reporting, please contact the RSC ([DAIDSRSCSafetyOffice@tech-res.com](mailto:DAIDSRSCSafetyOffice@tech-res.com)).

11.4.2 Reporting Requirements for this Study

The SAE Reporting Category, as defined in Version 2.0 of the DAIDS EAE Manual will be used for this study. The study agent for which expedited reporting is required is study-provided fluconazole.

11.4.3 Grading Severity of Events

The most current Division of AIDS Table for Grading the Severity of Adult and Pediatric Adverse Events (DAIDS AE Grading Table) is used and is available on the RSC website at <http://rsc.tech-res.com/safetyandpharmacovigilance/>.

11.4.4 Expedited AE Reporting Period

The expedited AE reporting period for this study is the entire study duration for each participant.

After the protocol-defined AE reporting period, unless otherwise noted, only suspected unexpected serious adverse reactions (SUSARs) as defined in Version 2.0 of the EAE Manual will be reported to DAIDS if the study staff become aware of the events on a passive basis (from publicly available information).

## 12.0 HUMAN PARTICIPANTS

### 12.1 Institutional Review Board (IRB) Review and Informed Consent

This protocol and the informed consent documents (Appendices II and III) and any subsequent modifications will be reviewed and approved by the IRB/EC responsible for oversight of the study.

A signed consent form will be obtained from the participant (or parent, legal guardian, or person with power of attorney, or person who is otherwise recognized as a proxy for participants who cannot consent for themselves, such as those below the legal age of consent). The consent form will describe the purpose of the study, the procedures to be followed, and the risks and benefits of participation. A copy of the consent form and any selected attachments will be given to the participant, parent, or legal guardian, and this fact will be documented in the participant’s record.

### 12.2 Participant Confidentiality

All laboratory specimens, evaluation forms, reports, and other records that leave the site will be identified by coded number only to maintain participant confidentiality. All records will be kept locked. All computer entry and networking programs will be done with coded numbers only. Clinical information will not be released without written permission from the participant, except as necessary for monitoring by the IRB/EC, the ACTG’s SMC, the FDA, the NIAID, the OHRP, industry supporter, or other country-specific government agencies.

### 12.3 Study Discontinuation

The study may be discontinued at any time by the ACTG, IRB/EC, the NIAID, the FDA, OHRP, industry supporter, or other country-specific government agencies as part of their duties to ensure that research participants are protected.

## 13.0 PUBLICATION OF RESEARCH FINDINGS

Publication of the results of this trial will be governed by ACTG policies.

## 14.0 BIOHAZARD CONTAINMENT

As the transmission of HIV and other blood-borne pathogens can occur through contact with contaminated needles, blood, and blood products, appropriate blood and secretion precautions will be employed by all personnel in the drawing of blood and shipping and handling of all specimens for this study, as currently recommended by the Centers for Disease Control and Prevention and the National Institutes of Health.

All dangerous goods materials, including diagnostic specimens and infectious specimens must be transported using packaging mandated by CFR 42 Part 72. Sites should also refer to individual carrier guidelines, eg, Federal Express, Airborne Express, for specific instructions.

## 15.0 REFERENCES

Anaissie EJ, Kontoyiannis DP, Huis C. et al., Safety, plasma concentrations, and efficacy of high-dose fluconazole in invasive mold infections J Infect Dis 1995;172:599-602.

Apseloff G, Hilligoss DM, Gardner MJ, et al. Induction of fluconazole metabolism by rifampin: in vivo study in humans. J Clin Pharmacol 1991;31(4):358-61.

Atkinson EN, Brown BW. Confidence limits for probability of response in multi-stage phase II clinical trials. Biometrics 1985;41:741-44.

Bicanic T, Meintjes G, Wood R, et al. Fungal burden, clearance of infection, and outcome in cryptococcal meningitis in antiretroviral-naïve and -experienced patients treated with amphotericin B or fluconazole. Clin Infect Dis 2007 Jul 1;45(1):76-80.

Bicanic T, Wood R, Meintjes G, et al. High-dose amphotericin B with flucytosine for the treatment of cryptococcal meningitis in HIV-infected patients: a randomized trial.Clin Infect Dis 2008;47(1):123-30.

Bronstein JA, Gros P, Hernandez E, Larroque P, Molinie C. Fatal acute hepatic necrosis due to dose-dependent fluconazole hepatotoxicity. Clin Infect Dis 1997;25:1266-7.

Brouwer AE, Rajanuwong A, Chierakul W, et al. Combination antifungal therapies for HIV-associated cryptococcal meningitis: feasibility and power of quantitative CSF cultures to determine fungicidal activity. Lancet. 2004;363(9423):1764-7.

Cato A 3^rd^, Cao G, Hsu A, et al. Evaluation of the effect of fluconazole on the pharmacokinetics of ritonavir. Drug Metab Dispos 1997;25(9):1104-6.

[Crerar-Gilbert A](http://www.ncbi.nlm.nih.gov/sites/entrez?Db=pubmed&Cmd=Search&Term="Crerar-Gilbert A"%5BAuthor%5D&itool=EntrezSystem2.PEntrez.Pubmed.Pubmed_ResultsPanel.Pubmed_DiscoveryPanel.Pubmed_RVAbstractPlus), Boots R, Fraenkel D, MacDonald GA. Survival following fulminant hepatic failure from fluconazole induced hepatitis. Anaesth Intensive Care 1999 27:650-52.

Dammert P, Bustamante B, Ticona E, Llanos-Cuentas A, Huaroto L, Chávez VM, Campos PE. Treatment of cryptococcal meningitis in Peruvian AIDS patients using amphotericin B and fluconazole. J Infect 2008 Sep;57(3):260-5.

Debruyne D, Ryckelynck J-P. Clinical pharmacokinetics of fluconazole. Clin Pharmacokinet 1993;24(1):10-27.

De Wit S, Debier M, De Smet M, et al. Effect of fluconazole on indinavir pharmacokinetics in human immunodeficiency virus-infected patients. Antimicrob Agents Chemother 1998;42(2):223-7.

Diflucan [package insert]. New York, NY: Roerig, Division of Pfizer Inc.; 2011.

Doering TL. How sweet it is! Cell wall biogenesis and polysaccharide capsule formation in Cryptococcus neoformans. Ann Rev Micro 63:223-47, 2009.

Dorsey ST, Biblo LA. Prolonged QT interval and torsades de pointes caused by the combination of fluconazole and amitriptyline. Am J Emerg Med 2000,18:227-229.

Ernest SC II, Hall SD, Jones DR. Mechanism-based inactivation of CYP3A by HIV protease inhibitors. J Pharmacol Exp Ther 2005;312:583–591.

Esch JJ, Kantoch MJ. Torsades de Pointes ventricular tachycardia in a pediatric patient treated with fluconazole. Pediatr Cardiol 2008,29:210-213.

Foulds G, Brennan DR, Wajszczuk C. Fluconazole penetration into cerebrospinal fluid in humans. Antimicrob Agents Chemother 1988;28:648-53.

Ford BJ. AIDS and Africa. Biologist. 2000;47(4):224.

French N. et al. Cryptococcal infection in a cohort of HIV-1 infected Ugandan adults. AIDS 2002;16:1031-38.

Galgiani JN, Catanzaro A, Cloud GA, et al. Fluconazole therapy for coccidioidal meningitis. The NIAID-Mycoses Study Group. Ann Intern Med 1993;119(1):28-35.

Gandhi PJ, Menezes PA, Vu HT, Rivera AL, Ramaswamy K. Fluconazole- and levofloxacin-induced torsades de pointes in an intensive care unit patient. Am J Health Syst Pharm 2003,60:2479-2483.

Gangaidzo IT, Mielke J, Matenga JA. Ethical considerations in the care of the patient with HIV/AIDS. Central African J Med 1999;45(2):51-3.

Geel J, Pitt J, Orrell CJ, et al. The effect of fluconazole on nevirapine pharmacokinetics. XV International AIDS Conference, July 11–16, 2004; Bangkok, Thailand. Abstract WeOrB1239.

Goodman JS, Kaufman L, Koenig MG. Diagnosis of cryptococcal meningitis. Value of immunologic detection of cryptococcal antigen. N Engl J Med 285(8):434-6, 1971 Aug 19.

Graninger W, Presteril E, Schneeweiss B, et al. Treatment of Candida albicans fungaemia with fluconazole. [J Infect](javascript:AL_get(this, 'jour', 'J Infect.');) 1993,26:133-46.

Gupta A, Lawrence AT, Krishnan K, Kavinsky CJ, Trohman RG. Current concepts in the mechanisms and management of drug-induced QT prolongation and torsade de pointes. Am Heart J 2007,153:891-899.

Hakim JG. et al. Impact of HIV infection on meningitis in Harare: a prospective study of 406 predominantly adult patients. AIDS 2000;14:1401-7.

Haubrich RH, Haghighat D, Bozzette SA, et al. High-dose fluconazole for treatment of cryptococcal disease in patients with human immunodeficiency virus infection. J Infec Dis 1994;170:238-242.

[Huang YW](http://www.ncbi.nlm.nih.gov/sites/entrez?Db=pubmed&Cmd=Search&Term="Huang YW"%5BAuthor%5D&itool=EntrezSystem2.PEntrez.Pubmed.Pubmed_ResultsPanel.Pubmed_DiscoveryPanel.Pubmed_RVAbstractPlus), Chang CC, Sun HY, et al. Primary adrenal insufficiency in patients with acquired immunodeficiency syndrome: report of four cases. J Microbiol Immunol Infect 2004 Aug;37(4):250-3.

International Conference on Harmonisation; Guidance for Industry: E14 Clinical Evaluation of QT/QTc Interval Prolongation and Proarrhythmic Potential for Non-Antiarrhythmic Drugs. US Dept of Health and Human Services. Fed Regist 2005,70:61134-61135.

[Jacobson MA](http://www.ncbi.nlm.nih.gov/sites/entrez?Db=pubmed&Cmd=Search&Term="Jacobson MA"%5BAuthor%5D&itool=EntrezSystem2.PEntrez.Pubmed.Pubmed_ResultsPanel.Pubmed_DiscoveryPanel.Pubmed_RVAbstractPlus), Hanks DK, Ferrell LD. Fatal acute hepatic necrosis due to fluconazole. [Am J Med](javascript:AL_get(this, 'jour', 'Am J Med.');) 1994;96(2):188-90.

Jarvis JN, Harrison TS, Govender N, et al. Routine cryptococcal antigen screening for HIV-infected patients with low CD4+ T-lymphocyte counts—time to implement in South Africa? South African Medical J 101(4):232-4, 2011 Apr.

Jarvis JN, Percival A, Bauman S, et al. Evaluation of a novel point-of-care cryptococcal antigen test on serum, plasma, and urine from patients with HIV-associated cryptococcal meningitis. Clin Infect Dis 2011;53:1019-23.

Kannankeril PJ, Roden DM. Drug-induced long QT and torsade de pointes: recent advances. Curr Opin Cardiol 2007,22:39-43.

Khazan M, Mathis AS. Probable case of torsades de pointes induced by fluconazole. Pharmacotherapy 2002,22:1632-1637.

Koks CHW, Crommentuyn KML, Hoetelmans RMW, et al. The effect of fluconazole on ritonavir and saquinavir pharmacokinetics in HIV-1-infected individuals. B J Clin Pharmacol 2001;51(6):631.

Larsen RA, Bauer M, Thomas AM. Graybill JR. Amphotericin B combined with fluconazole a potent combination for treatment of cryptococcal meningitis. Antimicrob Agents Chemother 2004;48(3):985-91.

Larsen RA, Bozzette SA, Jones BE, et al. Fluconazole combined with flucytosine for treatment of cryptococcal meningitis in patients with AIDS. Clin Infect Dis 1994;19(4):741-5.

Larsen RA, Leal MA, Chan LS. Fluconazole compared with amphotericin B plus flucytosine for cryptococcal meningitis in AIDS. A randomized trial. Ann Intern Med 1990;113(3):183-7.

Lindsley MD, Mekha N, Baggett HC, et al. Evaluation of a newly developed lateral flow immunoassay for the diagnosis of cryptococcosis. Clin Infect Dis 53(4):321-5, 2011 Aug.

Longley N, Muzoora C, Taseera K, et al. Dose response effect of high-dose fluconazole for HIV-associated cryptococcal meningitis in southwestern Uganda. Clin Infect Dis 2008;47:1556-61.

**Loyse A, Wilson D, Meintjes G, et al.** [**Comparison of the early fungicidal activity of high-dose fluconazole, voriconazole, and flucytosine as second-line drugs given in combination with amphotericin B for the treatment of HIV-associated cryptococcal meningitis.**](http://ovidsp.tx.ovid.com.libproxy.usc.edu/sp-3.5.1a/ovidweb.cgi?&S=ANEDFPCNLIDDFBNJNCALCEJCEIPAAA00&Complete+Reference=S.sh.42|4|1) **Clin Infec Dis*.* 2012 Jan1; 54(1):121-8.**

Makadzange A, Ndhlovu C, Takarinda, et al. Early versus delayed initiation of antiretroviral therapy for concurrent HIV infection and cryptococcal meningitis in sub-Saharan Africa. Clin Infect Dis 2010 Jun 1;50(11):1532-8.

Manosuthi W, Athichathanabadi C, Uttayamakul S et al. Plasma nevirapine levels, adverse events and efficacy of antiretroviral therapy among HIV-infected patients concurrently receiving nevirapine-based antiretroviral therapy and fluconazole. BMC Infect Dis 2007;7:14.

Marik PE, Kiminyo K, Zaloga GP. Adrenal insufficiency in critically ill patients with human immunodeficiency virus. Crit Care Med 2002 Jun;30(6):1267-73.

McKinsey DS, Kauffman CA, Pappas PG, et al. and the NIAID Mycoses Study Group. Fluconazole therapy for histoplasmosis. Clin Infect Dis 1996;23:996-1001.

Menichetti F, Fiorio M, Tosti A, et al. High-dose fluconazole therapy for cryptococcal meningitis in patients with AIDS. Clin Infect Dis 1996;22:838-40.

Milefchik E, Leal MA, Haubrich R, et al. Fluconazole alone or combined with flucytosine for the treatment of AIDS-associated cryptococcal meningitis. Medical Mycology. 46(4):393-5, 2008 Jun.

**Muzoora CK, Kabanda T, Ortu G, et al.** [**Short course amphotericin B with high dose fluconazole for HIV-associated cryptococcal meningitis.**](http://ovidsp.tx.ovid.com.libproxy.usc.edu/sp-3.5.1a/ovidweb.cgi?&S=ANEDFPCNLIDDFBNJNCALCEJCEIPAAA00&Complete+Reference=S.sh.42|2|1) **J Infect. 2012 Jan; 64(1):76-81.**

Mwaba P, Mwansa J, Chintu C, et al. Clinical presentation, natural history, and cumulative death rates of 230 adults with primary cryptococcal meningitis in Zambian AIDS patients treated under local conditions. Postgraduate Med J 2001;77(914):769-73.

Niwa T, Shiraga T, Takagi A. Effect of antifungal drugs on cytochrome P450 (CYP) 2C9, CYP2C19, and CYP3A4 activities in human liver microsomes. Biol Pharm Bull 2005;28(9):1805-08.

Okongo M. et al. Causes of death in a rural, population based human immunodeficiency virus type -1 natural history corhort in Uganda. Int ernational J Epidemiology 1998;27:698-702.

Pappas PG, Bustamante B, Ticona E, et al. Recombinant interferon- gamma 1b as adjunctive therapy for AIDS-related acute cryptococcal meningitis. J Infect Dis 2004; 189(12):2185-91.

Pappas PG, Bradsher RW, Kauffman CA, et al. Treatment of blastomycosis with higher doses of fluconazole. Clin Infect Dis 1997, 25:200-5.

**Pappas PG, Chetchotisakd P, Larsen RA, et al.** [**A phase II randomized trial of amphotericin B alone or combined with fluconazole in the treatment of HIV-associated cryptococcal meningitis.**](http://ovidsp.tx.ovid.com.libproxy.usc.edu/sp-3.5.1a/ovidweb.cgi?&S=ANEDFPCNLIDDFBNJNCALCEJCEIPAAA00&Complete+Reference=S.sh.42|16|1) **Clin Infec Dis*.* 2009 Jun 15; 48(12):1775-83.**

Pham CP, de Feiter PW, van der Kuy PH, van Mook WN. Long QTc interval and torsade de pointes caused by fluconazole. Ann Pharmacother 2006,40:1456-1461.

Pitisuttithum P, Tansuphasawadikul S, Simpson AJ, Howe PA, White NJ. A prospective study of AIDS-associated cryptococcal meningitis in Thailand treated with high-dose amphotericin B. J Infect 2001;43(4):226-33.

[Prasanthai V](http://www.ncbi.nlm.nih.gov/sites/entrez?Db=pubmed&Cmd=Search&Term="Prasanthai V"%5BAuthor%5D&itool=EntrezSystem2.PEntrez.Pubmed.Pubmed_ResultsPanel.Pubmed_DiscoveryPanel.Pubmed_RVAbstractPlus), Sunthornyothin S, Phowthongkum P, Suankratay C. Prevalence of adrenal insufficiency in critically ill patients with AIDS. J Med Assoc Thai 2007 Sep;90(9):1768-74.

Rex JH, Pappas PG, Karchmer AW, et al. A randomized and blinded multicenter trial of high-dose fluconazole plus placebo vs. fluconazole plus amphotericin B as therapy of candidemia and its consequences in non-neutropenic subjects. Clin Infect Dis 2003;36:1221-28.

Robinson PA, Bauer M, Leal MA, et al. Early mycological treatment failure in AIDS-associated cryptococcal meningitis. Clin Infect Dis 1999;28(1):82-92.

Roden DM. Drug-induced prolongation of the QT interval. N Engl J Med 2004,350:1013-1022.

Saag MS, Powderly WG, Cloud GA, et al. Comparison of amphotericin B with fluconazole in the treatment of acute AIDS-associated cryptococcal meningitis. The NIAID Mycoses Study Group and the AIDS Clinical Trials Group. N Engl J Med. 1992;326(2):83-9.

Sahai J, Gallicano K, Pakuts A, Cameron DW. Effect of fluconazole on zidovudine pharmacokinetics in patients infected with human immunodeficiency virus. J Infect Dis 1994;169:1103-07.

Takemasa H, Nagatomo T, Abe H, Kawakami K, Igarashi T, Tsurugi T, et al. Coexistence of hERG current block and disruption of protein trafficking in ketoconazole-induced long QT syndrome. Br J Pharmacol 2008,153:439-447.

Tett S, Moore S, Ray J. Pharmacokinetics and bioavailability of fluconazole in two groups of males with human immunodeficiency syndrome (HIV) infection compared with those in a group of males without HIV infection. Antimicrob Agents Chemother 1995;39:1835-41.

Tholakanahalli VN, Potti A, Hanley JF, Merliss AD. Fluconazole-induced torsade de pointes. Ann Pharmacother 2001,35:432-434.

Van der Horst CM, Saag MS, Cloud GA, et al. Treatment of cryptococcal meningitis associated with the acquired immunodeficiency syndrome. The NIAID Mycoses Study Group and AIDS Clinical Trials Group. N Engl J Med 1997;337(1):15-21.

[Walsh TJ, Finberg RW, Arndt C, et al. Liposomal amphotericin B for empirical therapy in patients with persistent fever and neutropenia. N Engl J Med 1999;340(10):764-71.](http://www.ncbi.nlm.nih.gov/entrez/query.fcgi?cmd=Retrieve&db=PubMed&list_uids=10072411&dopt=Abstract&itool=iconabstr)

Wassmann S, Nickenig G, Bohm M. Long QT syndrome and torsade de pointes in a patient receiving fluconazole. Ann Intern Med 1999,131:797.

**WHO Rapid Advice Diagnosis, Prevention and Management of Cryptococcal Disease in HIV -infected Adults, Adolescents and Children. December 2011.**

APPENDIX I

A5225/HiFLAC

Participant Information

SECTION 1: A5225/HiFLAC Study Visits

The study staff can answer any questions you have about individual study visits or about the evaluations that will occur. The table below can be used as a quick reference for you, along with the explanations that follow.

A. Study Schedule

| Evaluation or procedure | Screening^1^ | Entry^2^ | Most  Other  Visits^3^ | Special visits^4^ | Early discontinuation^5^ |
| --- | --- | --- | --- | --- | --- |
| Consent & contact information collected | √ |  | | | |
| CM confirmed | √ |  | | | |
| HIV confirmed | √ | |  | | |
| Electrocardiogram | √ |  | At day 4, week 1, and week 4 |  |  |
| Lumbar puncture | You may have a lumbar puncture at either of these visits. | | At week 2; possibly at most others until week 10 | √ |  |
| Physical exam | √ | √ | √ | √ | √ |
| Blood collected | √ | √ | √ | √ | √ |
| Urine collected |  | √ |  | possibly |  |
| Pregnancy test | √ | If pregnancy is suspected | | | |
| Neurological or Functional Assessments | √ | √ | √ | √ | √ |

^1^Screening Visit: After you have read and signed the consent form, you will have several evaluations done to make sure that you meet the requirements for joining the study.

^2^Entry Visit: If you are eligible to join the study, you will enter the study and receive your treatment assignment. At this visit you will find out if you will be taking only fluconazole or if you will take an amphotericin B-based treatment followed by fluconazole.

^3^Most other study visits: Most people will be seen for a study visit 1 day, 4 days, 1 week, 4 weeks, 6 weeks, 8 weeks, 10 weeks, and 24 weeks after entering the study. If you are taking amphotericin B, you may have to stay in a hospital or clinic the entire time you are receiving it (up to 17 days); study visits or blood collections will be performed during this time.

^4^Special study visits: If you are taking high dose fluconazole and not appearing to be getting better, you will be asked to come to the clinic for an extra visit. If at any time you appear to be getting sicker rather than better, you will be asked to come to the clinic for an extra visit.

^5^Early Discontinuation: If you stop taking fluconazole before the end of the study, or if you decide you cannot complete all the study visits, you will be asked to come in for an extra visit.

B. Explanation of Evaluations

Consent and contact information collected

After you read the consent and have had a chance to ask questions about the study, you will sign the consent form if you want to continue to be evaluated for study participation. You will also be asked how to be contacted in case you miss a visit or there are problems with your tests, and whether you give the study team permission to contact you.

CM confirmed

Your CM infection will be confirmed **at the study laboratory** using fluid collected from your spine. **If it is found that you do not have CM, you will stop study treatment immediately. Your health care provider will decide what treatment you should receive. You may stay in the study for about 2 weeks so that study staff can check for any side effects from the study treatment you took.**

HIV infection confirmed

If an HIV test has to be done, you may have to sign a separate consent form before this is done. You will be told the results of the HIV test as soon as it is available.

Electrocardiogram

An electrocardiogram, or ECG, is an electrical tracing of your heart that can show how hard it is working. You will have to lie very still for up to 10 minutes while the ECG is being done.

Lumbar Puncture

Spinal fluid will be collected through a needle placed into your spine in your lower back. This procedure is called a lumbar puncture, or LP. It may take about 45 minutes for the LP to be done. Afterwards, you may be asked to rest for a while so that the clinic staff can make sure that there are no side effects.

Physical examination

You will have a physical exam and will be asked questions about your health and about any medicines you have taken or are taking now.

Blood collected

Blood will be collected from you for various tests during the study. These include: routine lab tests, HIV viral load (a test that shows how much HIV is in your blood), CD4 count (a test that shows how many infection-fighting cells you have in your blood), liver function tests, levels of study drugs, and a test of how your adrenal glands are working. Your adrenal glands are organs that release hormones that control some important bodily functions.

Up to 45 mL (3 tablespoons) of blood may be collected at any one visit.

Before each of any visit when blood will be collected for levels of drugs, you will be asked to keep track of the time and day that you took all doses of certain non-study-provided drugs in the previous 3 days. The study staff will tell you which drugs you will need to keep track of.

Urine collected

Urine will be collected from you to be stored for later testing.

Pregnancy test

If you are a woman who is able to become pregnant, you may be asked to give a small urine or blood sample for a pregnancy test.

Neurological and functional assessments

At each visit, a clinician will evaluate your ability to do some simple things (like open your eyes and speak) on your own. At some visits, the study staff will ask you to answer questions about your activities. You will be asked about your current abilities and about your abilities just before you became sick. If you are unable to answer, a friend or family member may be asked. At some visits, you will also be asked to perform some simple tasks like touching your nose and walking a few steps.

SECTION 2: A5225/HiFLAC Study Design

The picture below is a timeline that shows what might happen during the study. The study staff will be able to describe each part to you.

Consent process

Study entry and randomization

Receive ampho B for up to 2 weeks, followed by fluconazole until study end.

Take fluconazole at a high dose for 4-10 weeks, followed by lower doses of fluconazole until study end.

Treatment with either fluconazole or amphotericin B

Up to 3 days

Up to 3 days

24 weeks total

SECTION 3: A5225/HiFLAC Benefits and Risks

A. Benefits

You may or may not benefit from being in this study. The treatment you will receive is designed to help you recover from CM, but no guarantee can be made. Information learned from this study may improve the ability of people to get treatment for CM, by finding a treatment that costs less, is easier to take, and is as effective as more expensive drug combinations or drugs that have more side effects.

B. Listed below are the most common side effects when taking any of these drugs. The staff will be able to tell you which are the most serious side effects. They will also be able to tell you what to do if you have any of these side effects.

| Drug | Side Effect |
| --- | --- |
| Fluconazole | - skin rash NOTE: The rash may be severe and rarely may cause death. - mild liver injury - abdominal pain - low platelets (cells in the blood that help blood clotting) - seizures (movements of your muscles that you cannot control) - diarrhea - upset stomach with or without vomiting - headache - temporary hair loss - sweating - dry skin - weight loss - constipation - skin darkening - abnormal heart beat   Rarely, side effects such as serious liver damage and even death have occurred in people taking fluconazole.   - liver abnormalities including hepatitis (inflammation of the liver) - fatigue - fever - increased blood pressure - dizziness - decreased appetite - abnormal accumulation of fluid in the body - decreased sexual desire - weakness - nausea - vomiting with low blood pressure   A serious allergic reaction to this drug is unlikely, but you should seek immediate medical attention if it occurs. Symptoms of a serious allergic reaction include: [rash](http://www.medicinenet.com/script/main/art.asp?articlekey=1992), itching, swelling, dizziness, trouble breathing. |
| Amphotericin B Deoxycholate (Ampho B) | - Fever and chills may occur 1 to 2 hours after the infusion is started. This is more common with the first few doses and should subside as therapy continues - upset stomach - diarrhea - weakness - muscle aches or pains - vision change - tingling of the hands or feet - ringing in the ears - rapid or irregular heartbeat - low blood pressure or changes in blood pressure - wheezing or shortness of breath - chest pain - loss of appetite - vomiting - headache - low red blood cell counts which may cause you to feel tired and fatigued - mild kidney damage, which may lead to swelling of hands and feet and loss of minerals in the urine - redness, soreness, or local bruising where amphotericin B is put into a vein in your arm.   Many of these side effects might occur only when you are being given the ampho B. Some, like the kidney damage and bruising, will last for a while longer, but will not be permanent. |

| Drug | Side Effect |
| --- | --- |
| Flucytosine | - nausea - vomiting - [diarrhea](http://www.medicinenet.com/script/main/art.asp?articlekey=1900) - [headache](http://www.medicinenet.com/script/main/art.asp?articlekey=20628) - Itching - [dry mouth](http://www.medicinenet.com/script/main/art.asp?articlekey=43095) - Weakness - fatigue - chest pain - mental/mood changes - hearing loss - tingling of the hands or feet - uncontrollable movements - seizures - change in amount of urine   A serious allergic reaction to this drug is unlikely, but seek immediate medical attention if it occurs. Symptoms of a serious allergic reaction include: [rash](http://www.medicinenet.com/script/main/art.asp?articlekey=1992), itching, swelling, dizziness, trouble breathing. |

Fluconazole Together With Other Drugs

If you and your doctor decide that you need to take anti-HIV drugs, you can, but you should not take nevirapine (NVP) until your dose of fluconazole has been reduced to 400 mg daily or less.

Once your dose of fluconazole has been reduced to 400 mg daily or less, you may take NVP. Only fluconazole will be provided by the study. Anti-HIV drugs will not be provided by the study. When you take NVP at the same time as fluconazole, you may have some of the following side effects:

- dizziness
- mood or sleep changes
- confusion
- agitation or anxiety
- hallucinations
- depression
- abnormal thinking
- suicidal thoughts
- rash
- liver damage

There is very little information about the safety of using NVP together with fluconazole, especially with fluconazole given at high doses. The effects of high-dose fluconazole with other medications that you may be taking for AIDS are not known either.

Risks of Blood Drawing

Taking blood may cause some discomfort, bleeding, or bruising where the needle enters the body, lightheadedness, and in rare cases, fainting or infection.

Special Risks Related to Breast-feeding

Fluconazole is known to pass through the breast milk and may cause harm to your infant. You will not be able to continue taking fluconazole if you are breast-feeding.

APPENDIX II

DIVISION OF AIDS

AIDS CLINICAL TRIALS GROUP (ACTG)

SAMPLE CONCISE INFORMED CONSENT

A5225/HiFLAC

A Phase I/II Dose-Finding Study of High-Dose Fluconazole Treatment in AIDS-Associated Cryptococcal Meningitis, FINAL Version 2.0, dated 05/11/12

Short title: A5225/HiFLAC, High-Dose Fluconazole Treatment in AIDS-Associated CM, FINAL Version 2.0, dated 05/11/12

INTRODUCTION

You are being asked to take part in this research study because you have been diagnosed with cryptococcal meningitis (CM), and it appears to your doctor that you are also infected with HIV (the virus that causes AIDS). CM is a disease caused by a fungus that infects tissue around your brain.

WHY IS THIS STUDY BEING DONE?

This study is being done to see if taking fluconazole (a drug that is provided as pills to be taken by mouth) alone at a higher dose than is now approved is a tolerable, safe, and effective treatment for CM for up to 10 weeks. Depending on how effective this treatment is in individual study participants, the dose of fluconazole could be lowered before week 10. The study will also collect information about treating CM with amphotericin B (alone or with another drug, **either** flucytosine **or fluconazole**). Amphotericin B is a drug that must be given to you through a vein in your arm.

Fluconazole alone at high doses for up to 10 weeks is an experimental treatment for CM. Some studies have tested some high doses in a small number of people, but more information is needed about the side effects and effectiveness. Amphotericin B, either alone or with flucytosine **or fluconazole**, is the **recommended** CM treatment for the first 2 weeks. After 2 weeks of amphotericin B, participants in this study will receive fluconazole **alone** according to the local standard of treatment. In some cases, the dose of fluconazole that participants receive may be experimental. The study staff will be able to tell you about the dose of fluconazole that you will receive.

Up to 192 people may take part in this study in various countries.

WHAT DO I HAVE TO DO IF I AM IN THIS STUDY?

If you decide to join this study, you will need to be seen in the clinic about 10 times in 6 months. The evaluations required at most visits will take up to half a day to complete. You may need to come to the clinic for additional visits if you develop side effects, if your condition gets worse, or if you switch drugs. There are no experimental procedures. *[Details of the study procedures may be provided in A5225/HiFLAC Study Visits, in a separate document.]*

If you decide not to take part in this study or if you do not meet the eligibility requirements, we will still use some of your information. As part of this screening visit, demographic (e.g., age, gender, race), clinical (for example, disease condition, diagnosis), and laboratory (for example, HIV viral load) information is being collected from you so that ACTG researchers may determine whether there are patterns or common reasons why people do not join a study.

The study will include two separate stages, Stage 1 and Stage 2. You will participate in only one stage. *[Details about the design of each stage are in A5225/HiFLAC Study Design, in a separate document.]*

At the study entry visit, you will be assigned to one of these two initial treatment groups:

Study Entry

*Experimental Treatment:*

*Fluconazole Alone: at a dose of 1200 mg/day*, 1600mg/day, or 2000mg/day*

**As of 7 October 2011, this group is finished enrolling.*

Standard Treatment: Amphotericin B (with or without flucytosine **or fluconazole**), followed by fluconazole **alone**

Your assignment will be random, like the flip of a coin. Depending on when you join the study, you will have up to 3 chances in 4 of being assigned to the experimental treatment. *[Additional information can be provided in A5225/HiFLAC Study Design, in a separate document.]* You will not be able to choose your group, but both you and your doctor, as well as the study staff, will know which group you are in.

**Most of the f**luconazole **used in the study** will be provided through the study. **A**mphotericin B**,**  flucytosine**, and fluconazole for use in combination with Amphotericin B** will be provided through the study. *[Sites may add information about provision of these drugs here.]*

**Shortly after you enter the study, tests done at the study laboratory may show that you do not have CM. If this happens, you will stop all study treatment immediately. Your health care provider will decide what treatment you should receive. You may stay in the study for about 2 weeks so that study staff can check for any side effects from the study treatment you took.**

CM can be diagnosed by examining spinal fluid which is collected through a needle placed into your spine in your lower back. This procedure is called a lumbar puncture, or LP. You will have several LPs while on study. *[More information may be provided in A5225 Study Visits, in a separate document.]* Each time you have an LP for the study, some of your spinal fluid will be collected and tested for CM.

If you are taking amphotericin B, your treatment will be changed after about 2 weeks. If you are taking fluconazole and CM is not found in your spinal fluid after several weeks of treatment, your treatment may be changed. In most cases, this change will be to a lower dose of fluconazole (probably to 400 mg fluconazole).

At week 10, everyone will start taking 200 mg per day of fluconazole, which is the currently approved dose at this stage of treatment for CM.

New tests are being developed to identify CM. You will be asked to provide a urine sample to be used in one or more of the new tests. Some of your blood and spinal fluid will also be used in new tests. Because these tests are not approved in the US, you will not be given the results.

You will receive the results of routine laboratory tests that are performed during the study.

At some point in the study, your doctor may decide you should start taking anti-HIV drugs. The study will not provide anti-HIV drugs. Blood samples will be collected at one or more visits to check the level of fluconazole when taken alone and when taken with efavirenz (for your HIV infection) and/or rifampin (in the event you are treated for tuberculosis).

CM treatment after the study

After you complete your study visits, the study will no longer provide you with fluconazole. Efforts will be made by your doctor to find a way to continue fluconazole after the study is over. Continued treatment is expected but cannot be guaranteed by the study. *[Sites should insert information about post-trial provision of fluconazole.]*

WHY WOULD THE DOCTOR TAKE ME OFF STUDY TREATMENT OR OFF THIS STUDY EARLY?

The study doctor may need to take you off fluconazole or amphotericin B without your permission if:

- continuing one of these drugs may be harmful to you
- you become pregnant or start breastfeeding
- you need a treatment that you may not take while taking either fluconazole or amphotericin B
- you are not able to take study treatment as required
- **after you enter the study, tests show that you do not have CM.**

If you have to stop taking study treatment early, you may still remain on study and have most study evaluations. You and your doctor should discuss this option.

The study doctor may need to take you off the study early without your permission if:

- the study is stopped or canceled.
- a Study Monitoring Committee (SMC) recommends that the study be stopped early. An SMC is a group of experts who are not involved in the study, but who monitor it.
- you are not able to attend the study visits as required by the study.
- you do not start study treatment within 3 days after entering the study.
- your doctor thinks the study is no longer in your best interest.
- after you enter the study, your screening tests show that you are not infected with HIV or are do not have CM.

WHAT ARE THE RISKS OF THE STUDY?

Drugs

You may have side effects while taking fluconazole or amphotericin B or flucytosine. Some of these are listed in a separate document: A5225/HiFLAC Risks & Benefits. These lists include only the more serious or common side effects with a known or possible relationship. If you have questions concerning additional drug side effects, please ask the medical staff at your site.

Serious and/or life threatening side effects could occur when some other medications are taken with fluconazole. For your safety, you must tell the study doctor or nurse about all medications you are taking before you start the study and also before starting any new medications while on the study. Also, you must tell the study doctor or nurse before enrolling in any other clinical trials while on this study.

Risks of LPs

When the needle is first inserted, you may feel a shooting pain and/or a tingling sensation. You may develop an allergic reaction to the substances used to clean or numb the place where the needle will go in. It is possible that you could develop an infection as a result of the LP. You may have a headache that lasts more than a day. You may have pain in your lower back for a short time (several hours to a day). There may be swelling where the needle was inserted. Though it is unlikely, the doctor may need to inject some of your blood around the place where the needle was inserted to stop a spinal fluid leak.

ARE THERE RISKS RELATED TO PREGNANCY?

Fluconazole is known to harm unborn babies at daily doses of 400 mg and more. This dose is one of the doses you might take during this study. You may also be taking higher doses of fluconazole during the study.

While you are taking study-provided fluconazole, you must agree that birth control is used if you have sex that could lead to pregnancy. You may discuss the birth control methods with the study staff. You may not use an oral hormone-based contraceptive while taking study-provided fluconazole.

If you are having sex that could lead to pregnancy, you must agree not to become pregnant, or, if you are a man, you must agree not to attempt to make a woman pregnant or participate in sperm donation. If you are a woman, then you must agree that two methods of birth control are used at the same time if you have sex that could lead to pregnancy.

You may choose from the birth control methods listed below.

1. Male or female condoms with or without a cream or gel that kills sperm
2. Diaphragm or cervical cap with a cream or gel that kills sperm
3. Intrauterine device (IUD)
4. Injectable Depo Provera
5. Tubal ligation

Some birth control drugs that are given as pills, shots, or placed on or under the skin may not be safe to take with fluconazole and may not be effective if taken alone.

Birth control must be used until 6 weeks after you stop taking fluconazole. If you are a man, you must agree not to participate in sperm donation during this period of time.

If you become pregnant while on study, the study staff would like to obtain information from you about the outcome of the pregnancy (even if it is after your participation in the study ends). If you are taking anti-HIV drugs when you become pregnant, your pregnancy will be reported to an international database that collects information about pregnancies in women taking anti-HIV drugs. This report will not use your name or other information that could be used to identify you.

WHAT OTHER CHOICES DO I HAVE BESIDES THIS STUDY?

*[Sites should insert general information about local/national CM treatment availability.]*

High dose fluconazole or amphotericin B with or without flucytosine **or fluconazole**, laboratory tests to monitor the effectiveness of these drugs, and quality medical care may or may not be available to you outside the study. The clinic staff will discuss with you other treatment choices in your area and the risks and the benefits of all the choices.

WHAT ABOUT CONFIDENTIALITY?

The study team will provide you with an identification number. This identification number (not your name or other information that could be used to identify you) will be used for laboratory tests or blood work stored for testing in future studies. Your medical records and the list of names, addresses, and identification numbers will be kept in a locked room. Only the study staff will have access. Any publication of this study will not use your name or identify you personally.

Efforts will be made to keep your personal information confidential. We cannot guarantee absolute confidentiality. Your personal information may be disclosed if required by law. Your records may also be reviewed by the ACTG’s Study Monitoring Committee (SMC), US Food and Drug Administration, the local institutional review board (IRB) or ethics committee (EC) (*insert name of site IRB/EC*), the Office for Human Research Protections (OHRP), U.S. National Institutes of Health (NIH), your country’s national health agency or other regulatory authorities, industry supporter or designee, study staff, and study monitors.

*[Delete the following for non-US sites.]* In addition to the efforts of the study staff to help keep your personal information private, we have gotten a Certificate of Confidentiality from the U.S. Federal Government. This certificate means that researchers cannot be forced to tell people who are not connected with this study, such as the court system, about your participation. Also, any publication of this study will not use your name or identify you personally. Having a Certificate of Confidentiality does not prevent you from releasing information about yourself and your participation in the study. Even with the Certificate of Confidentiality, if the study staff learns of possible child abuse and/or neglect or a risk of harm to yourself or others, we will be required to tell the proper authorities.

WHAT ARE THE COSTS TO ME?

There will be no cost to you for study-related visits, physical examinations, laboratory tests or other procedures. You, your insurance company, or your health care system may need to assume the cost of drugs not provided by the study. *[Delete references to insurance company or health care system if not applicable at site.]*  In some cases, it is possible that your insurance company or health care system will not pay for these costs because you are participating in a research study.

WHAT HAPPENS IF I AM INJURED?

If you are injured as a result of being in this study, you will be given immediate treatment for your injuries, and you will be referred for further treatment, if necessary. However, you may/may not *(per site/country policy)* have to pay for this care. There is no program for compensation either through *[this institution]* or the US NIH. You will not be giving up any of your legal rights by signing this consent form.

WHAT ARE MY RIGHTS AS A VOLUNTEER IN A RESEARCH STUDY?

Taking part in this study is completely voluntary. You may choose not to take part in this study or leave this study at any time. You will be treated the same no matter what you decide.

We will tell you about new information from this or other studies that may affect your health, welfare, or willingness to stay in this study. If you want the results of the study, let the study staff know.

WHAT IF I HAVE QUESTIONS OR PROBLEMS?

For questions about this study or a research-related injury, contact:

- *name & telephone number for the investigator or other study staff*

For questions about your rights as a volunteer in a research study contact:

- *(name or title of person on the IRB/EC or other organization appropriate for the site*
- *telephone number of above)*

SIGNATURE PAGE for Concise Consent form for A5225/HiFLAC, High-Dose Fluconazole Treatment for AIDS-Associated CM

If you have read this consent form (or had it explained to you), all your questions have been answered, and you agree to take part in this study, please sign your name below.

Participant’s Name (print) Participant’s Signature and Date

Participant’s Guardian/Proxy (print) Guardian’s/Proxy’s Signature and Date

(As appropriate)

Study Staff Conducting Consent Study Staff Signature and Date Discussion (print)

Witness’s Name (print) Witness’s Signature and Date

(As appropriate)

Some of your blood, urine, and spinal fluid that is left over after all required study testing is done may be stored (with usual protectors of identity) and used for ACTG-approved HIV-related research. Please indicate below whether you agree to have your leftover blood, urine, and spinal fluid stored for such research. Your decision will not affect your participation in this study nor the care you receive from your own caregivers. Note that unless your site is not able to ship samples, they will be stored at the ACTG repository in the U.S.

Initial or sign on ONE line below:

I agree to have my blood, urine, and spinal fluid stored for ACTG-approved HIV-related research.

________________________

OR

I do not agree to have my blood or urine or spinal fluid stored for any reason.

________________________

APPENDIX III

DIVISION OF AIDS

AIDS CLINICAL TRIALS GROUP (ACTG)

SAMPLE INFORMED CONSENT

For

WOMEN WHO BECOME PREGNANT WHILE ON STUDY

For protocol:

A5225/HiFLAC: A Phase I/II Dose-Finding Study of High-Dose Fluconazole Treatment in AIDS-Associated Cryptococcal Meningitis, FINAL Version 2.0, dated 05/11/12

SHORT TITLE FOR THE STUDY: A5225/HiFLAC, High-Dose Fluconazole for AIDS-Associated CM, FINAL Version 2.0, dated 05/11/12

INTRODUCTION

Because you are now pregnant, you are being asked whether you want to continue taking part in this research study. This study was designed so that women who were pregnant could not join the study. However, because you were already in the study when you became pregnant, you will be allowed to stay in the study if you choose to. Depending on what part of the study you are in, you may be asked to stop taking study-provided drugs.

This is a consent form. It gives you more information about this study and how it may affect your pregnancy and your baby. The study staff will talk with you about this information. You may also talk with your own doctor about what is best for you and your baby, and if you should remain on anti-HIV drugs. If you agree to stay in this study, you will be asked to sign this consent form. You will get a copy to keep. You are free to ask questions of the study staff at any time.

WHAT DO I HAVE TO DO IF I STAY IN THIS STUDY?

If you choose to remain in the study, you will have to discontinue study-provided fluconazole. You and your doctor will decide what treatment you should receive. You will continue to attend the study visits listed in the main consent. You will have the evaluations described in the main consent with one exception: for the remainder of the study, you will not have blood collected to check the level of fluconazole.

This study will not provide care related to your pregnancy, the delivery of your baby or the care of your baby. You must arrange for your care and your baby’s care outside of this study.

Long-term follow-up is recommended for a baby whose mother takes anti-HIV drugs during pregnancy. The study staff will talk with you about long-term follow-up and the possibility of enrolling your baby in a long-term follow-up study.

WHAT ARE THE RISKS RELATED TO STAYING IN THE STUDY?

Fluconazole is known to harm unborn babies at doses of at least 400 mg daily. This dose is one of the doses you might take during this study. You may also be taking higher doses of fluconazole during the study.

You will continue to have LPs while pregnant and the risks related to these are the same as described in the main consent.

BREAST-FEEDING

Because this study is only about 6 months long, it is unlikely that you will still be on study after delivery.

ARE THERE BENEFITS TO STAYING IN THIS STUDY?

If you continue to take part in this study, there is not likely to be any direct benefit to you or your baby. Information learned from this study may help others who have HIV and CM.

WHAT OTHER CHOICES DO I HAVE?

You have the choice of:

- treatment with prescription drugs available to you
- treatment with experimental drugs being studied for use during pregnancy, if you qualify
- no treatment

Please talk to your doctor about these and other choices available to you. Your doctor will explain the risks and benefits of these choices.

WHY WOULD THE DOCTOR TAKE ME OFF THIS STUDY EARLY?

The study doctor may need to take you off the study early without your permission once you are pregnant if:

- the study is stopped or cancelled
- a Study Monitoring Committee (SMC) recommends that the study be stopped early (an SMC is a group of experts who monitor the study.)
- you are not able to attend the study visits as required by the study
- staying on the study no longer appears to be in your best interest

You will not be required to attend any extra visits if you leave the study early while pregnant.

WHAT ABOUT CONFIDENTIALITY?

Efforts will be made to keep your personal information confidential. We cannot guarantee absolute confidentiality. Your personal information may be disclosed if required by law. Any publication of this study will not use your name or identify you personally.

Your records may be reviewed by the ACTG’s SMC, US Food and Drug Administration (FDA), the local institutional review board (IRB) or ethics committee (EC) (*insert name of site IRB/EC)*, US National Institutes of Health (NIH), the Office for Human Research Protections (OHRP), your country’s national health agency or other regulatory authorities, industry supporter or designee, study staff, and study monitors.

*(Delete the following for non-US sites.)* The study’s main consent, which you signed before entering the study, contains information about a Certificate of Confidentiality. You may want to review this information now.

WHAT ARE THE COSTS TO ME?

In addition to any costs that are described in the study consent you already signed; this study will not cover any cost related to your pregnancy, delivery of your baby or care of your baby.

WHAT HAPPENS IF MY BABY OR I AM INJURED?

If your baby or you are injured as a result of being in this study, you will both be given immediate treatment for your injuries. The cost for this treatment will be charged to you or your insurance company. There is no program for compensation either through this institution or the US NIH. You will not be giving up any of your legal rights by signing this consent form.

WHAT ARE MY RIGHTS AS A RESEARCH SUBJECT?

Continuing to take part in this study is completely voluntary. You may choose not to continue in this study or leave this study at any time. You will be treated the same no matter what you decide.

We will tell you about new information from this or other studies that may affect your health, welfare or willingness to stay in this study. If you want the results of the study, let the study staff know.

WHAT DO I DO IF I HAVE QUESTIONS OR PROBLEMS?

For questions about this study or a research-related injury, contact:

- *insert name of the investigator or other study staff*
- *insert telephone number of above*

For questions about your rights as a research subject, contact:

- *insert name or title of person on the IRB/EC or other organization appropriate for the site*
- *insert telephone number of above*

SIGNATURE PAGE for Pregnancy Consent for A5225/HiFLAC, High-Dose Fluconazole Treatment for AIDS-Associated CM

If you have read this consent form (or had it explained to you), all your questions have been answered and you agree to take part in this study, please sign your name below.

____________________________ __________________________________________

Participant’s Name (print) Participant’s Signature and Date

____________________________ __________________________________________

Participant’s Guardian (print) Guardian’s Signature and Date

(As appropriate)

____________________________ _________________________________________

Study Staff Conducting Study Staff Signature and Date

Consent Discussion (print)

____________________________ _________________________________________

Witness’s Name (print) Witness’s Signature and Date

(As appropriate)
